# A cosmopolitan inversion facilitates seasonal adaptation in overwintering *Drosophila*

**DOI:** 10.1093/genetics/iyad207

**Published:** 2023-12-05

**Authors:** Joaquin C B Nunez, Benedict A Lenhart, Alyssa Bangerter, Connor S Murray, Giovanni R Mazzeo, Yang Yu, Taylor L Nystrom, Courtney Tern, Priscilla A Erickson, Alan O Bergland

**Affiliations:** Department of Biology, University of Virginia, 90 Geldard Drive, Charlottesville, VA 22901, USA; Department of Biology, University of Vermont, 109 Carrigan Drive, Burlington, VT 05405, USA; Department of Biology, University of Virginia, 90 Geldard Drive, Charlottesville, VA 22901, USA; Department of Biology, University of Virginia, 90 Geldard Drive, Charlottesville, VA 22901, USA; Department of Biology, University of Virginia, 90 Geldard Drive, Charlottesville, VA 22901, USA; Department of Biology, University of Virginia, 90 Geldard Drive, Charlottesville, VA 22901, USA; Department of Biology, University of Virginia, 90 Geldard Drive, Charlottesville, VA 22901, USA; Department of Biology, University of Virginia, 90 Geldard Drive, Charlottesville, VA 22901, USA; Department of Biology, University of Virginia, 90 Geldard Drive, Charlottesville, VA 22901, USA; Department of Biology, University of Virginia, 90 Geldard Drive, Charlottesville, VA 22901, USA; Department of Biology, University of Richmond, 138 UR Drive, Richmond, VA 23173, USA; Department of Biology, University of Virginia, 90 Geldard Drive, Charlottesville, VA 22901, USA

**Keywords:** adaptive tracking, inversions, seasonality, balancing selection, *Drosophila melanogaster*

## Abstract

Fluctuations in the strength and direction of natural selection through time are a ubiquitous feature of life on Earth. One evolutionary outcome of such fluctuations is adaptive tracking, wherein populations rapidly adapt from standing genetic variation. In certain circumstances, adaptive tracking can lead to the long-term maintenance of functional polymorphism despite allele frequency change due to selection. Although adaptive tracking is likely a common process, we still have a limited understanding of aspects of its genetic architecture and its strength relative to other evolutionary forces such as drift. *Drosophila melanogaster* living in temperate regions evolve to track seasonal fluctuations and are an excellent system to tackle these gaps in knowledge. By sequencing orchard populations collected across multiple years, we characterized the genomic signal of seasonal demography and identified that the cosmopolitan inversion In(2L)t facilitates seasonal adaptive tracking and shows molecular footprints of selection. A meta-analysis of phenotypic studies shows that seasonal loci within In(2L)t are associated with behavior, life history, physiology, and morphological traits. We identify candidate loci and experimentally link them to phenotype. Our work contributes to our general understanding of fluctuating selection and highlights the evolutionary outcome and dynamics of contemporary selection on inversions.

## Introduction

Species living in rapidly fluctuating environments are exposed to spatially and temporally varying selection (Bell 2010). If species harbor polymorphisms that are beneficial in 1 selective environment but not the other, local adaptation will be evident from shifts in allele frequency across space and time, a process known as adaptive tracking (Botero *et al.* 2015). Context-dependent fitness effects can result in the long-term maintenance of functional genetic variation in populations and between species (Hedrick 1976), and can also drive the rapid turnover of new, transiently balanced polymorphisms (Bürger and Gimelfarb 2002). Recent theoretical work demonstrates that multilocus adaptive tracking is possible (Wittmann *et al.* 2017), leaves distinct molecular signatures at linked sites (Wittmann *et al.* 2023), and can be facilitated by ecological factors such as seasonal population booms and busts (Bertram and Masel 2019). Moreover, empirical studies have provided evidence that adaptive tracking can be quantified in natural and experimental populations (reviewed in Johnson *et al.* 2023). Yet, we still have a limited understanding of the ecological drivers that underlie adaptive tracking, its effects on genetic diversity, and its genetic architecture.

Fruit flies (*Drosophila melanogaster*) living in temperate habitats are a premier system for understanding the evolutionary dynamics of adaptive tracking. Fruit flies have short generation times (∼10 to 15 days), produce many generations per year (∼15 generations; Pool 2015), and experience fluctuating selection across the changing seasons (Behrman *et al.* 2015). For example, variations in stress tolerance and life history enable some individuals to better survive the winter months while others more effectively exploit resources in the growing season (Behrman *et al.* 2015; Rajpurohit *et al.* 2018; Erickson *et al.* 2020; *cf.* Yu and Bergland 2022). These observations suggest that seasonal adaptation operates through a resource-allocation tradeoff between reproduction and survival that is also mirrored across latitudinal gradients (Schmidt and Conde 2006). Genomic analyses have supported this hypothesis and identified thousands of loci whose allele frequencies (AF) change between seasons across multiple localities and display parallel shifts in allele frequency across spatial gradients (Bergland *et al.* 2014; Machado *et al.* 2021; Rodrigues *et al.* 2021). While these findings have highlighted that seasonal adaptive tracking is a quantifiable phenomenon across fly genomes, identifying candidate genes of interest underlying seasonal adaptation has remained a challenge. An issue-driven in part by a lack of dense temporal resolution across these seasonal datasets that have primarily focused on paired spring-fall sampling (Machado *et al.* 2021). Nonetheless, in a recent analysis of seasonal sampling across 2 continents, Machado *et al.* (2021) identified the breakpoints of cosmopolitan inversions, particularly of the 10 Mb In(2L)t inversion, as regions enriched for loci that evolve by seasonal adaptive tracking. This is a notable finding given that adaptive loci that exist as chromosomal inversions have been extensively studied for decades and were among the first examples of loci underlying adaptations to a fluctuating environment (Dobzhansky and Wright 1943; Charlesworth 2016; Kapun *et al.* 2023).

In this paper, we use a combination of population and quantitative genetics to study short-term demography and seasonal evolution in *D. melanogaster*. We address 3 basic questions: (1) What are the impacts of seasonal population booms and busts on patterns of standing genetic variation in fruit flies? (2) Are inversions enriched for signals of seasonal adaptive tracking, compared to the rest of the genome? And, (3) what are the candidate loci and candidate phenotypes associated with seasonal selection in overwintering *Drosophila*? To answer these questions, we combined a densely sampled genomic time-series collected in Charlottesville, VA (USA) with previously published fly genomic datasets, including the *Drosophila* Evolution over Space and Time (DEST) dataset that contains samples from multiple temperate populations worldwide (Kapun *et al.* 2021). This new dataset from Charlottesville, VA, represents a valuable addition to existing panels of temporal variation in this species (e.g. Bergland *et al.* 2014; Machado *et al.* 2021), as it is composed of samples collected every 2 weeks from late spring to late fall across 3 years. This dataset allows for seasonal analyses of adaptation and demography with much greater levels of granularity beyond the paired spring-fall scheme of previous studies. Using these data, we characterized genomic signatures of seasonal population expansions and contractions across the genome (i.e. “boom-and-bust” demography; Ives 1970; Biémont 1985). Then, we identified regions of the genome associated with seasonal changes that exceed expectations based on chance and demographic history, paying special attention to inversions including In(2L)t. Finally, using a meta-analysis of the *Drosophila* Genetics Reference Panel (DGRP, Mackay *et al.* 2012), we show that dozens of phenotypes are affected by In(2L)t and experimentally validate the association between In(2L)t inversion status and 1 ecologically important phenotype.

Overall, our data reveals rapid evolutionary changes in response to seasonally varying selection and suggests connections between phenotype, genotype, and the environment at In(2L)t. We show that *Drosophila* populations experience strong bouts of drift resulting from annual cycles of boom-and-bust demography. Allele frequency shifts through time are correlated with variation in aspects of temperature weeks prior to collection, and the inversion is associated with a host of ecologically important phenotypes. These results suggest that we can differentiate the footprints of natural selection from the background signal of boom-and-bust demography. Moreover, our work also provides insight into the evolution of adaptive inversions more generally by showing that adaptive alleles within the inversion are both old trans-species and trans-continental polymorphisms, as well as young and population-specific. This finding suggests that 2 types of selection may have occurred at In(2L)t: balancing selection, operating at the level of the inversion across continents, and directional selection operating at the levels of specific populations that may drive adaptive fine-tuning in response to local conditions.

## Materials and methods

### Fly sampling

New samples for this study were collected at an orchard in Charlottesville, VA (Carter Mountain Orchard, 37.99N, 78.47W) from 2016 to 2019. Collections from 2016 to 2018 were done using aspirators and netting every 2 weeks starting in mid-June when peaches come into season in central VA and ending in mid-December at the end of the fall apple season. The collection in 2019 was done at the beginning of the growing season in June. Because *D. melanogaster* is phenotypically similar to its sister taxa *D. simulans*, we determined species identity using the male offspring produced from isofemale lines set from wild-caught flies. *D. melanogaster* isofemale offspring were frozen in ethanol and stored at −20°C prior to sequencing.

### DNA extraction, sample preparation, and sequencing

We prepared 2 sets of samples: Pool-seq samples, and individual DNA-seq libraries. All libraries were made using G1 male offspring from wild-caught isofemale lines. For pool-seq, we prepared 37 libraries (see the number of pooled flies in Supplementary Table 1). Pool-seq sequencing, filtering, and mapping were done following the protocols outlined in (Kapun *et al.* 2021) using the DEST dockerized pipeline (https://github.com/DEST-bio/DEST_freeze1). Individual DNA libraries were made from samples collected in 2016, 2018, and 2019. For 2016, we prepared 119 individual samples collected across the growing season. For 2018, we prepared libraries from 43 individuals collected in the fall (2018 November 29). For 2019, we prepared libraries collected from 41 samples in the spring (2019 June 14). Both 2018 and 2019 libraries were built using a Nextera reduced-volume protocol (Baym *et al.* 2015). Sequencing of the 2016 individuals was done on an Illumina HiSeq X (2 × 150 bp; paired-end configuration). Sequencing for the 2018 and 2019 individuals was done on an Illumina Novaseq (2 × 150 bp). Reads were mapped to the *D. melanogaste*r genome (NCBI acc. GCA_000001215.4). For the individual sequences, data were processed using a bioinformatics pipeline that includes samtools/bcftools (Li *et al.* 2009), Picard, and the Genome Analysis Toolkit (GATK; Van der Auwera and O’Connor 2020). Additional bioinformatic details can be found in Supplementary Methods 1, and in our GitHub repository (https://github.com/Jcbnunez/Cville-Seasonality-2016-2019).

### Other *D. melanogaster* datasets used

*D. melanogaster* data was downloaded from 4 public repositories: DEST (Kapun *et al.* 2021), *Drosophila* Genetic Reference Panel (DGRP v2; Mackay *et al.* 2012), Global Diversity Lines (GDLs; Grenier *et al.* 2015), and *Drosophila* Population Genomics Project (DPGP v3; Pool *et al.* 2012; Lack *et al.* 2015). In addition, we used resequenced inbred line data from Maine and Pennsylvania (PA) (Behrman *et al.* 2018). We used FlyBase (Gramates et al. 2022; release FB2023_06) to find information on gene functions and phenotypes.

### Temporal analysis using principal component analyses

**Fig. 1.**
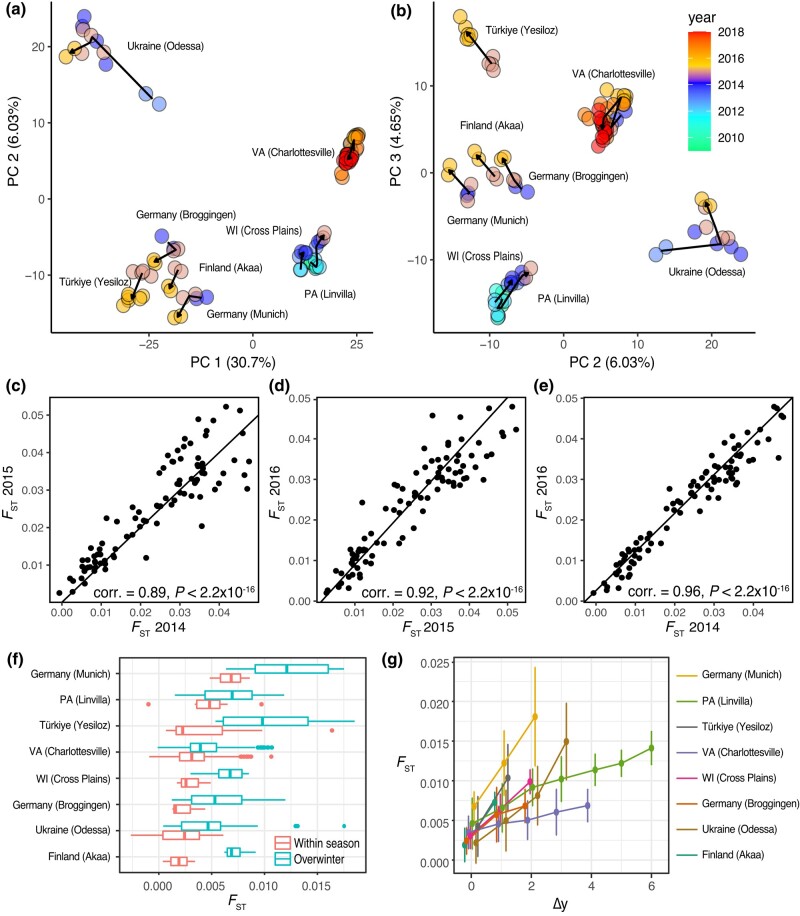
Population structure and signatures of overwintering in temperate flies. a) PCA of temporal samples (PCs 1, 2). The arrow path indicates the temporal identity of the samples (arrowheads show the most recent samples, and origins show the oldest sample). Percent variance explained (PVE) is shown in parentheses on each axis. b) Same as A, but PCs 2 and 3. c, d, e) Pairwise *F*_ST_ for samples collected at the same localities across 3 years (2014–2016) in DEST. Correlation values are shown as an inset. Each point represents a pairwise comparison between 2 samples collected at the exact same locality across pairs of years (2014 vs 2015 for A; 2015 vs 2016 for B; 2014 vs 2016 for c). The diagonal line represents the 1-to-1 expectation for a perfect correlation. f) Genome-wide average *F*_ST_ across all within the growing season comparisons and overwinter. In this context, the growing season is defined as the period occurring between the late spring to late fall seasons and the overwintering period is defined as the time frame between early winter and early spring. g) *F*_ST_ values (±1 standard deviation) across multiple years of collection (Δy is the difference in years of the collections).

**Fig. 2.**
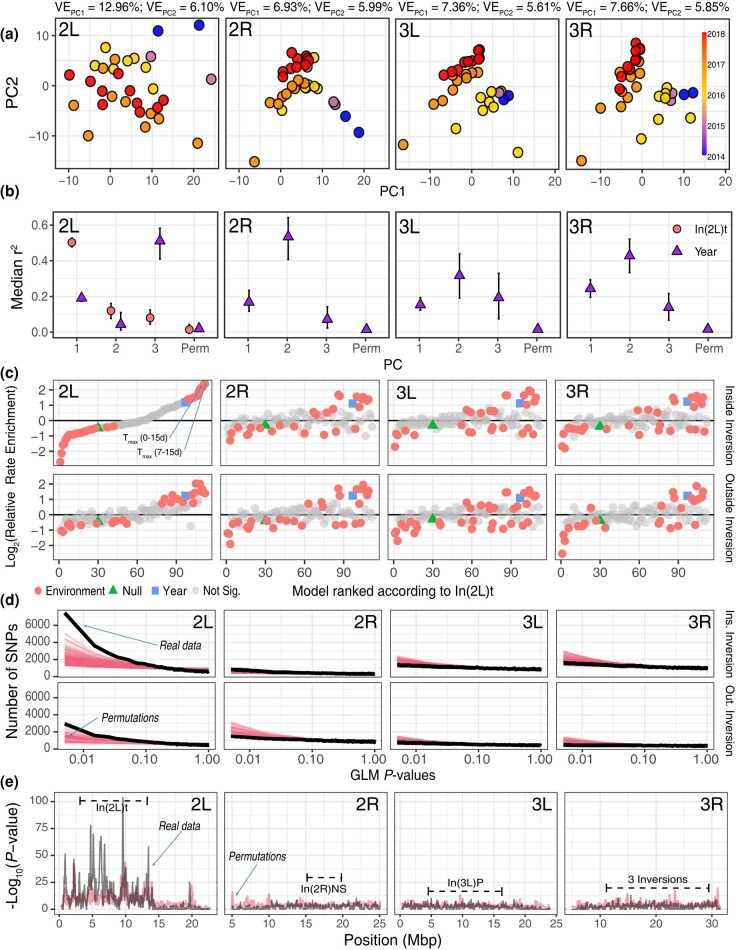
Drivers of temporal structure in Charlottesville flies. a) PCAs separated by chromosome arm. Each point represents 1 sample, and the color indicates the year of collection. The amount of variance explained by each PC is shown at the top of the panel. b) Median correlation between PCs 1–3, built using 1,000 randomly sampled SNPs 500 times, and the frequency of In(2L)t as well as the year of collection. Permutations are indicated as “Perm”. c) Environmental models for Charlottesville samples. Each point represents a model relating a different environmental variable to AF inside inversions (top row) or outside inversions (bottom row). The x-axis shows models ranked according to the best model for SNPs inside In(2L)t (top left facet). The y-axis shows the relative rate of enrichment compared to permutations of the environmental Generalized Linear Models (GLMs). Gray circles mean that the confidence intervals contain the null hypothesis of 1. Red circles indicate that the model is statistically significant. Blue squares are the year-only model. Green triangles are the null model. d) *P*-value distribution of the T_max_0–15d GLM model (black line), T_max_0–15d GLM permutation (pink lines), across chromosomes inside (top) and outside (bottom) inversions. e) *P*-value of the signal enrichment test across the genome of *D. melanogaster* (Window Size = 0.1 Mb, step = 50 Kb). Major cosmopolitan inversions are highlighted with horizontal dashes. The 3 inversions in 3R include: In(3R)K, In(3R)Mo, and In(3R)P. The pink line shows the 99th quantile of all T_max_0–15d GLM permutation.

To characterize patterns of spatial and temporal genetic variation across the temperate range of *D. melanogaster,* we performed principal component analyses (PCA) as implemented in R's FactoMiner v2.6 package (Lê *et al.* 2008). Principal Component Analyses (PCAs) were conducted on the pool-seq time series data combining Charlottesville data with that of DEST (Fig. 1a and b; Supplementary Fig. 1). For these analyses, we applied a minimum allele frequency filter of 1% across populations. We also applied a mean effective coverage (*N*_eff_) filter of 28 (see explanation below). *N*_eff_ was calculated as in (Kolaczkowski *et al.* 2011; Feder *et al.* 2012):


(1) $$N_{\mathrm{eff}}=(n_{\mathrm{reads}}\times n_{\mathrm{chrs}}-1)/(n_{\mathrm{reads}}+n_{\mathrm{chrs}})$$

where *n*_reads_ is the read depth, and *n*_chrs_ is the number of pooled chromosomes. *N*_eff_ is calculated in a single nucleotide polymorphism (SNP)-wise manner, and the mean *N*_eff_ for a given sample is used in our filtering. The *N*_eff_ filter of 28 was determined empirically by running the PCA analysis at various *N*_eff_ thresholds. When samples with *N*_eff_ < 28 are included in the analyses these samples create outliers in PCA driven by *N*_eff_. When PCA is done with samples *N*_eff_ > 28, *N*_eff_ no longer influenced clustering across major Principal Components (PCs; results not shown). We randomly sampled SNPs in increments of 100, from 100 to 1,000 SNPs, and in increments of 1000, from 1,000 to 20,000 SNPs, performed PCA, and calculated correlations of PC 1, 2, and 3 with the year of collection, frequency of In(2L)t, and *N*_eff_. In parallel, we ran an identical analysis but with the sample labels permuted. We repeated this process 500 times each and compared correlation values for the real ordering of the data relative to permutations (Figs. 2; Supplementary Figs. 2 and 3).

### Forward genetic demographic simulations

To test if overwintering bottlenecks influence patterns of genetic differentiation through time, and to infer minimum and maximum population sizes during boom-and-bust cycles that are consistent with our data, we performed genetic simulations. First, we performed a coalescent-based neutral simulation of a single population with θ_π_ = 0.001 using *msprime* (Baumdicker *et al.* 2022) in Python 3.8. This neutral background was used as a burn-in within the forward genetics software, SLiM 3 (Haller and Messer 2019). SLiM 3 was used to simulate cyclic population crashes while varying the population size maximum (N_Max_) and the population size minimum (N_Min_) under a model of the instantaneous change in population size (Supplementary Fig. 4a). For each parameter combination, the simulated population had a constant size at N_Max_ from generations 1–16, 19–33, and 36–50 and the bottlenecks occurred at generations 17–18 and 34–35 where the population size was set to N_Min_ (Supplementary Fig. 4b). A Variant Call Format (VCF) file of 50 simulated diploid individuals was output at the end of each generation to track allele frequency changes. AF were simulated to mimic pooled sequencing using *poolSeq* v0.3.5 (Taus *et al.* 2017) with a mean coverage of 60. Pairwise *F*_ST_ was calculated using *poolfstat* v2.1.1 (Gautier *et al.* 2022). Every parameter combination was simulated 100 independent times with different seeds. Parameter estimation was performed using Approximate Bayesian Computation (ABC) using the local linear regression method (*loclinear*) with a tolerance threshold of 5% using *abc* v2.1 (Csilléry *et al.* 2012) in R. The summary statistics used were the medians of within year *F*_ST_, between year *F*_ST_, and the correlation (*R*^2^) of PC1, LD1, and LD2 values relative to the simulation year (Supplementary Fig. 4c and d). These later 3 statistics are, respectively, the principal component (PC) projections of dimensions 1 (i.e. PC1), and the first and second linear discriminants (i.e. LD1–2) of a discriminant analysis of principal components (DAPC; Jombart *et al.* 2010), using the simulated year as a grouping prior. For PCA, we used a matrix of AF (columns) and samples (rows) in the *PCA()* function from *FactoMineR*. The first and second PC values from each sample were extracted and used in a simple linear regression with simulation year (Years 1–3) to calculate correlations (i.e. PC1 ∼ Year, PC2 ∼ Year). We repeated this step for both the first and second linear discriminant (LD) axes as well. First, a matrix of AF and samples was used in the *dapc* function in *adegenet* v2.1.10 (Jombart *et al.* 2010) with simulation year as a grouping prior. After extracting LD1 and LD2 values, we ran a linear regression with the LD values and simulation year. In this way, we were able to measure how the severity of yearly bottlenecks affects both PC and LD space due to shifts in AF across samples. A leave-one-out analysis was performed on the input summary statistics to understand how each contributes to the estimates of N_Max_ and N_Min_. Our analyses show that the LD1 and LD2 statistics are most strongly affecting the estimates of N_Max_ and N_Min_ from ABC (Supplementary Fig. 4e).

### Identification and inference of In(2L)t markers

We assessed the frequency of In(2L)t using 2 separate procedures. For the pool-seq dataset, we used the method outlined by (Kapun *et al.* 2020, 2021). For the individually sequenced flies, we developed a predictive framework. We used the DGRP to identify a panel of SNPs associated with inversion breakpoints for In(2L)t using only lines karyotyped as standard or inverted homozygotes (Huang *et al.* 2014). We identified putative inversion markers on 2L using PCA and by estimating levels of linkage disequilibrium (LD) using Plink v1.9 (Purcell *et al.* 2007). We only kept SNPs with the highest loadings to PC1 and mean LD > 0.99 relative to the inversion karyotype, representing 36,283 SNPs out of 901,524 (4.0%). We refined this list by identifying a subset of SNPs that are also in strong LD with each other in the Charlottesville data. We kept 47 SNPs with the highest linkage disequilibrium relative to each other (*r*^2^ > 0.8) in the Charlottesville data as a list of final inversion markers (Supplementary File 1). We trained a linear support vector machine model (SVM) using the 47 markers and the DGRP data using the R package “e1071” v1.7-11 (Meyer *et al.* 2023), and used this SVM to perform *in-silico* karyotyping of the individually sequenced samples (Supplementary Fig. 5).

### Environmental association tests using generalized linear models

To characterize the association between allele frequency change and seasonal environmental change, we fit a binomial generalized linear model (GLM) using *fastglm* v0.0.3 (Marschner 2011). We modeled allele frequency change separately in 4 phylogeographic regions using the Charlottesville and DEST samples. For each SNP and each region, we fit 112 models. In these models, we used *N*_eff_ (1) as the observed sample size for each population sample and SNP. First, we fit a “null model” in which AF were regressed onto their means:


(2) $$AF=\beta_{0}+\varepsilon$$

and a second “time model” where AF were regressed onto the collection year, or year nested inside the collection locale, as an unordered factor:


(3.1) $$\mathrm{AF}=\beta_{0}+\beta_{1}(\mathrm{yea}r_{\mathrm{factor}})+\varepsilon$$

(3.2) $$\mathrm{AF}=\beta_{0}+\beta_{1}(\mathrm{yea}r_{\mathrm{factor}}:\;\mathrm{localit}y_{\mathrm{factor}})+\varepsilon$$

This time model was designed to capture changes in AF driven by boom-and-bust population dynamics. We used equation 3.1 to model the Charlottesville data, which came from a single locality, and equation 3.2 to model 3 phylogeographic partitions of the DEST data (Europe-East, Europe-West (EU-W), North America; Supplementary Table 1).

Next, we constructed 110 “environmental models”:


(4.1) $$\mathrm{AF}=\beta_{0}+\beta_{1}(\mathrm{yea}r_{\mathrm{factor}})+\beta_{2}(\gamma_{i})+\varepsilon$$

(4.2) $$\mathrm{AF}=\beta_{0}+\beta_{1}(\mathrm{yea}r_{\mathrm{factor}}:\;\mathrm{localit}y_{\mathrm{factor}})+\beta_{2}(\gamma_{i})+\varepsilon$$

where γ_i_ is an environmental covariate. For any model, the environmental covariate is a summary (mean or variance) of temperature, precipitation, or humidity across 11-time windows (see Supplementary Fig. 6). These windows encompass time frames ranging from 0–7 days (∼½ the generation time of *Drosophila*), to 0–90 days (∼6 generations). For temperature, we also calculated the maximum and minimum temperature in the selected window of time and the proportion of days where the daily maximum was above 32°C or the minimum daily temperature was below 5°C, per the thermal limit model of Machado *et al.* (2021; these variables are indicated in Supplementary Fig. 6 as “prop. max” and “prop. min”, respectively). Hourly estimates of environmental variables were obtained from the National Aeronautics and Space Administration (NASA)-power dataset (Sparks 2018). We performed likelihood ratio tests (LRT) between each environmental model and the year-only model as well as between the year-only model and the null model (Supplementary Data 1). For each SNP, we identified the best model using the Akaike Information Criterion (AIC).

### Permutations

**Fig. 3.**
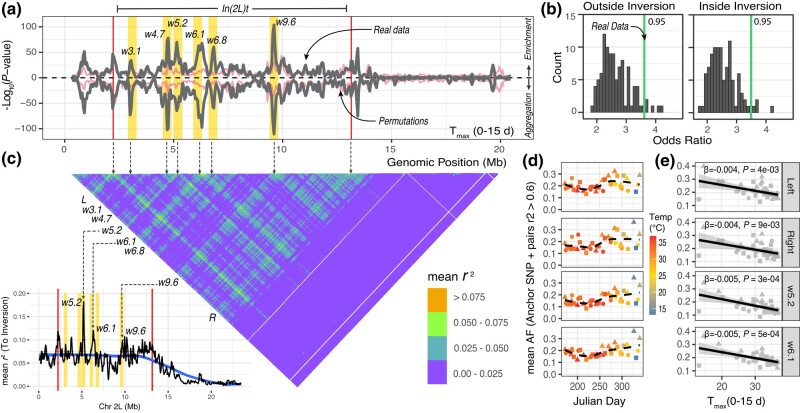
Signals of seasonal selection and high linkage in In(2L)t. a) Two tests of enrichment for the T_max_0–15d model. The top portion of the panel shows the *P*-value of the signal enrichment test. The bottom portion shows the *P*-value of the aggregation test. The pink line shows the 99th quantile of all permutations of the T_max_0–15d GLM. The black line shows the real data. The windows of interest are highlighted in yellow. In(2L)t is demarcated by the vertical lines. b) Enrichment between the top 5% T_max_0–15d SNPs and the top 5% XtX_ST_ values in chromosome 2L is represented as an odds ratio. The histogram is the distribution with respect to the T_max_0–15d GLM permutations. The green line is the real data. The number in the corner is the percentile of T_max_0–15d GLM permutations where the real data lie. c) Mean pairwise LD across chromosome 2L. LD values are reported in bins as described in the legend. Windows of interest are matched to panel a and indicated with arrows. White lines indicate windows with no SNPs (size = 50 Kbp, step = 10 Kbp). (C-inset) Mean linkage disequilibrium of SNPs across 2L to the inversion. The red lines indicate the breakpoints. The black lines indicate the mean *r*^2^ across windows of 0.1 Mb. The blue line is the moving average across windows. Windows of interest are highlighted in yellow. d) Mean allele frequency plots of the anchor loci relative to time (Julian day) for selected example windows (Left [5′ breakpoint], Right [3′ breakpoint], w5.2, w6.1). e) Regression analyses on mean AF of anchor SNPs as a function of temperature. In panels d and e, the circles represent samples from 2016, triangles from 2017, and squares from 2018.

We used permutations to develop null expectations for signals of environmental association for the GLM analysis. First, we shuffled the year (or year: locality) term across samples and performed an LRT between the “time model” and the “null model.” This permutation allows us to test if the *year-*only model or *year: locality* model is observed as the best model more than expected relative to the permutations. Next, we shuffled the environmental variable across samples but kept the *year* (or *year: locality*) term as in the real ordering of the data. This permutation allowed us to test if any environmental factor was associated with allele frequency change more than expected from chance. We performed an LRT between the permuted “environmental model” and the real “time model.” For each SNP, in each of the 4 population clusters, we ran 100 permutations for each model. For each permutation, and for each SNP in each cluster, we identified the best model by AIC. The specific reordering of an environment for the *i*th permutation was the same for each SNP, therefore this routine preserves linkage. Because the permutations preserve linkage, they are not only useful for generating a null distribution of gene-environment association (Fig. 2c and d) but also for sliding window tests of signal enrichment and aggregation (Figs. 2e and 3a). Variation in the test-statistics across the genome reflects differences in power due to the variable number of SNPs under consideration and differences in linkage along the chromosome. See Supplementary Fig. 6c for more details on the permutation scheme.

### Model enrichment

We tested whether some models were found as the best model more often than expected relative to the permuted time and environmental GLMs. To demonstrate this, we partitioned the genome into 8 regions: 4 autosomal arms and partitioning inside or outside inversions. For the real ordering and the permutations, we counted the number of SNPs where each model was found as the best model. For the *i^th^* model under consideration, in each of the 8 regions, we calculated the relative rate (*rr*) of model enrichment, across all *n* permutations for model *i*, as:


(5) $${rr}_{i,n}=log_{2}\left(\frac{N_{real_{i}}}{N_{perm\;i,n}}\right)$$

with mean:


(6) $${\bar{rr}}_{i}=\frac{1}{n}\;\overset{100}{\underset{n=1}{\sum }}\;log_{2}\left(\frac{N_{real_{i}}}{N_{perm\;i,n}}\right)\;\;$$

and standard deviation:


(7) $$\sigma_{rr,i}=\sqrt{\frac{\sum_{n=1}^{100}\;{\left({rr}_{i}-{\bar{rr}}_{i}\right)}^{2}}{n}}$$

where *N_real i_* is the number of SNPs for which a given model was found to be the best model by AIC in the analysis. *N_perm i_* is the number of SNPs for which a permuted model was found to be the best model in the *n^th^* permutation. We calculated the mean (6) standard deviation (7) of *rr* across all *n* permutations. If, for any given model, the mean-log_2_ transformed rate of enrichment ±2 times the standard deviation did not include 0, the model was considered significantly enriched (${\bar{rr}}_{i}$ ± 2**σ***_rr, i_* > 0) or under-enriched (${\bar{rr}}_{i}$ ± 2**σ***_rr, i_* < 0) relative to a null distribution generated by the GLM permutations. Once the “best model” was found in Charlottesville (i.e. the temperature maximum, 0–15 days prior to collection; T_max_0–15d; see Results section: *In(2L)t shows signatures of adaptive tracking…*), we sought to identify regions of the genome that harbored a localized enrichment of this environmental association using a signal enrichment test and a *P*-value aggregation test.

### Signal enrichment and *P*-value aggregation tests

To test the hypothesis that SNPs with low *P*-values were evenly distributed through the genome, we ranked and normalized *P*-values such that the distribution is transformed into a uniform distribution bounded between 1 and 1/L (where L is the number of SNPs studied; Lotterhos *et al.* 2017). By using these rank-normalized *P*-values and dividing the genome into 0.1 Mb windows with a 50 Kb step, we identified genomic windows that harbor an excess of SNPs with the smallest 5% of the GLM *P*-values. We calculated a *P*-value of “signal enrichment” for each window under the null hypothesis that 5% of SNPs in the window will be amongst the most significant 5% genome-wide using binomial tests and report the *P*-value from that test (Figs. 2e and 3a).

To assess the strength of the GLM signal across the genome, we aggregated *P*-values using the Weighted-Z Analysis (WZA) metric (Booker *et al.* 2023), which is based on Stouffer's method for combining *P*-values (Stouffer *et al.* 1949) (Figs. 2e and 3a). We ran the WZA test on the real data as well as the permuted GLM results (pink line in Figs. 2d, e and 3a).

We used the *P*-values from the signal enrichment and aggregation tests as test-statistics and compared them to an empirical null distribution generated from the permutations. We ran the signal enrichment and *P*-value aggregation tests for permuted GLM results of the best environmental model for any phylogeographic cluster. For each window, we calculated the distribution of test statistics and generated the upper 1.0% as a critical value for identifying windows where the real data beat permutations.

### BayPass analysis

We used *BayPass* v2.4 (Gautier 2015; Olazcuaga *et al.* 2022) to identify loci that are strongly differentiated through time and whose AF are strongly correlated with T_max_0–15d. We used *poolfstat* v2.2 (Gautier *et al.* 2022) to create the input files for *BayPass*. To control for population structure in the data, *BayPass* uses the Ω relatedness matrix. To ensure computational efficiency for our analyses, the Ω matrix was constructed using genetic data thinned such that only 1 randomly selected SNP in every 2,000 bp window was retained across chromosomes 2L, 2R, 3L, and 3R. We performed 5 replicate runs of the *core* model to estimate XtX_ST_ and took the mean of the XtX_ST_ values across independent runs per SNP. Under neutrality, XtX_ST_ values follow a ***χ***^2^ distribution with degrees of freedom equal to the number of populations sampled (Olazcuaga *et al.* 2022), and we corrected *P*-values for multiple testing using the *qvalue* package (Storey *et al.* 2010). We performed gene-environment association analysis using the standard covariate model with T_max_0–15d as the covariate. We performed 5 independent runs of the standard covariate model and averaged the Bayes Factor (BF) terms across replicate runs. To construct null distributions of the BF values, we performed simulations of pseudo-observed data (POD) using the *simulate.baypass()* function. We performed 10 separate simulations of ∼570,000 SNPs, using the observed Ω matrix. Using these simulated values, we show that BF values of ∼6 correspond to a False Discovery Rate (FDR) of 0.001 and we use this as a critical threshold for assessing significant associations with T_max_0–15d. For all analyses, we used default MCMC options.

### Inferring haplotype trajectories

We inferred haplotype trajectories associated with loci of interest by combining information from our pooled dataset and our individually sequenced dataset. For each window of interest (see *Model enrichment*), we identified a set of “anchor markers.” These markers are strongly associated with T_max_0–15d (Benjamini-Hochberg FDR *q*-value < 0.05) in the pooled data. Then, using the LD estimates from the individual data, we identified all loci pairs (±0.2 Mb) with *r*^2^ > 0.6 in Virginia to the anchor locus. We used the averaged frequency of the anchor loci and its high LD pairs, within any given window, as estimators of haplotype frequency in the pooled data (see Supplementary Data 3 and Fig. 3d and e).

### Population genetic analyses for *D. melanogaster*

**Fig. 4.**
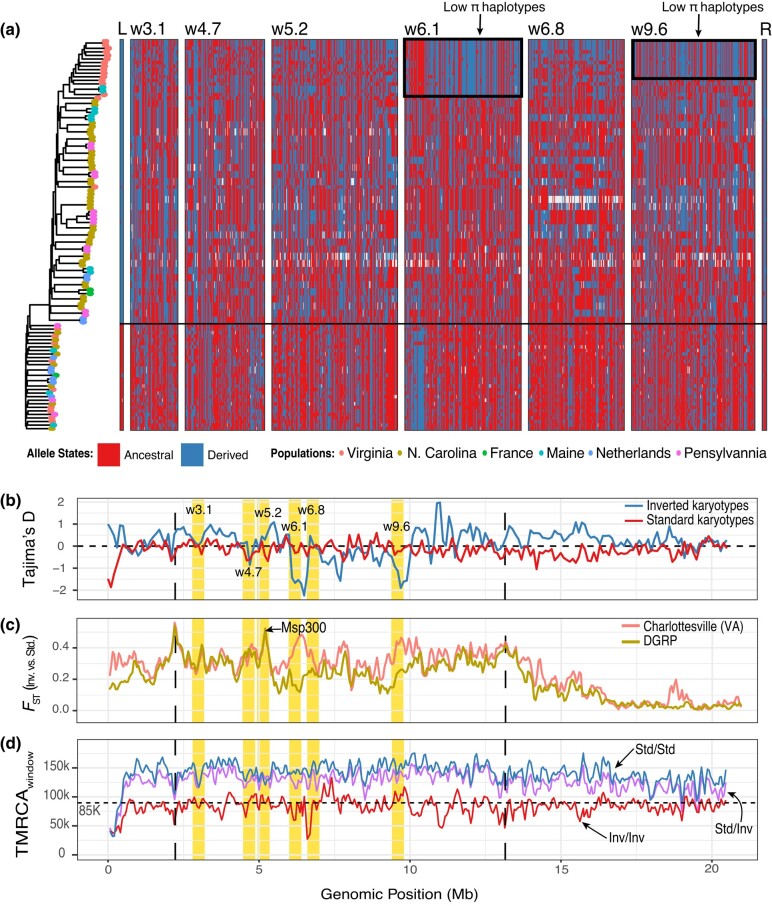
Signatures of selection on outlier windows. a) Haplotype structure plot for inversion breakpoints and windows of interest. Only samples homozygous for the inversion and for the standard karyotype are shown. Samples are sorted and grouped according to the phylogeny built using candidate SNPs across windows shown to the left. The ancestral state was determined relative to *D. simulans*. The horizontal line divides inverted (top) vs standard (bottom) karyotypes. Sequences with low levels of genetic diversity (which create lower π estimates within the inverted karyotypes) are indicated in a black frame. b) Tajima's D across 2L for inverted and standard karyotypes. c) *F*_ST_ between the inverted and standard karyotypes in 2L for Charlottesville and the DGRP. d) Allele ages across 2L. In(2L)t breakpoints are indicated as black lines. The estimated age of the inversion breakpoints, ∼85,000 y, is marked as a dashed horizontal line. Windows of interest are annotated with yellow bands. Dashed vertical lines indicate the boundaries of In(2L)t.

For individually sequenced flies, we calculated *F*_ST_, π, Tajima's D, and haplotype numbers in *vcftools* v0.1.16 (Danecek *et al.* 2011; e.g. Fig. 4b and c, Supplementary Figs. 9 and 10). We estimated 2 types of LD metrics. First, we estimated SNP-to-SNP (i.e. pairwise) LD using *plink* v1.9 (Purcell *et al.* 2007; Fig. 3c). Second, we calculated inversion-to-SNP levels of LD (see Fig. 3c-inset). This was done using a similar framework as pairwise SNP, yet instead of using a second locus in the formula, we used the inversion status. Time to the most recent common ancestor (TMRCA) was calculated using *GEVA* v1.0 (Albers and McVean 2020; Fig. 4d). For pool-seq data, *F_ST_* was calculated using *poolfstat* v2.1.1 (Gautier *et al.* 2022). Temporal *F_ST_* was calculated among populations sampled across time points in Charlottesville and selected DEST populations (Germany, Munich, and Broggingen; Türkiye, Yesiloz; Ukraine, Odessa; Finland, Akaa; USA, PA, Linvilla and Wisconsin [WI], Cross Plains; Fig. 1f-g). Spatial *F_ST_* in Europe was calculated on samples collected during the fall of 2015 to ensure temporal homogeneity across comparisons (Fig. 6c).

### Population genetic analyses for other drosophilids

Genomic information for other drosophilids was obtained as follows: *D. simulans* was obtained as a VCF file from a Zenodo repository (Signor *et al.* 2018). Data for *D. yakuba* was obtained from (Turissini and Matute 2017) and mapped to its corresponding genome (NCBI acc. GCA_016746365.2). Data for *D. sechellia* was obtained from (Schrider *et al.* 2018) and mapped to its corresponding genome (NCBI acc. GCF_004382195.1). Data for *D. mauritiana* was obtained from (Garrigan *et al.* 2014) and mapped to its corresponding genome (NCBI acc. GCA_004382145.1). The *Msp300* gene in other *Drosophila* species was identified using pairwise sequence homology relative to *D. melanogaster*, using Exonerate v2.2.0 (Slater and Birney 2005).

### Cross-model enrichment and directionality scores

We used Fisher's Exact Test (FET) to assess whether candidate loci that show strong signals of enrichment for environmental association in Charlottesville are enriched for those SNPs in the top 5% identified in the best environmental association models in other regions of the world. We conducted this comparison separately for 3 phylogeographic clusters: EU-W, Europe-East (EU-E), and North America-East (NoA-E) (Fig. 6a; Supplementary Figs. 11 and 12). We also assessed if allele frequency changes are consistent between population sets by calculating the proportion of SNPs that have the same sign of allele frequency change with respect to the population cluster's best-fit model, conditional on SNPs having strong allele frequency change (top 5% in both population clusters; Fig. 6b). We refer to this statistic as the “directionality” statistic following Erickson *et al.* (2020), Machado *et al.* (2021), and Yu and Bergland (2022). Directionality scores are calculated by comparing the sign of the regression coefficients for the 2 models. For any given SNP, the sign of the beta terms from these models can be identical (positive-positive, negative-negative), or it can be opposite (positive-negative, negative-positive). For any comparison, we calculate directionality as the proportion of SNPs under consideration that have identical signs. Directionality values of either 0% or 100% indicate that alleles at a candidate window are changing in frequency as a haplotype block in Charlottesville relative to another cluster (i.e. 100% means that blocks always move in the same direction whereas 0% means that they always move in opposite directions). The null expectation is 50%.

### Matched controls analysis

Matched controls are loci with similar recombination rates (±0.20 cm/Mb) and global allele frequency (±0.030) compared to candidate SNPs. For every SNP of interest, we aimed to identify up to 100 matched controls. To avoid the impact of genetic draft as well as linkage disequilibrium to major cosmopolitan inversions, we sampled matched controls from chromosomes different from the 1 containing the SNP (Fig. 6c).

### Phenotypic association with inversion status

To infer the phenotypic consequences of In(2L)t and candidate loci associated with the best model in Charlottesville (T_max_0–15d), we conducted a Genome-wide association study (GWAS) meta-analysis using 225 published phenotypic measurements of the DGRP (see Supplementary Tables 8 and 9). We annotated these phenotypes by classifying each 1 into 4 general groups: “Behavior”, “Life-History”, “Morphology”, and “Stress-resistance”. We used this dataset to establish the effect of the cosmopolitan inversions In(2L)t, In(2R)Ns, In(3L)P, In(3R)K, In(3R)Payne, In(3R)Mo on phenotype using linear models designating inversion presence focusing on DGRP strains reported to be homozygous inverted, or homozygous standard (Fig. 7a, Supplementary Fig. 13a). In the case of 3R, the analysis was implemented to identify traits associated with any inversion inside the chromosome. To test for the association between inversion status and phenotype, we used a linear model and recorded the number of times that each inversion had a significant effect on phenotype with a nominal *P-*value < 0.05. We performed 1,000 permutations by shuffling the inversion genotype status of the DGRP lines and repeating the analysis. We tested whether the number of significant phenotype associations with an inversion in the real data was greater than these permutations.

We performed GWAS for each phenotype using the DGRP2 genomic dataset with GMMAT v1.3.2 (Chen *et al.* 2019). In this analysis our “null model” is described by the formula:


(8) $$\mathrm{Phenotyp}e_{i}=\beta_{0}+\beta_{1}(\mathrm{Wolbachia})+\mathrm{GRM}$$

The null model is compared to a “full model” defined as:


(9) $$\mathrm{Phenotyp}e_{i}=\beta_{0}+\beta_{1}(\mathrm{Wolbachia})+\beta_{2}(\mathrm{SNP}\;\mathrm{dosage})+\mathrm{GRM}$$

where β_1_(Wolbachia) is a fixed effect corresponding to the Wolbachia infection status, and GRM is a random effect genetic relatedness matrix. To generate a GRM, we first performed LD pruning using the *snpgdsLDpruning()* function in SNPRelate version 3.17 (Zheng *et al.* 2012) with the *slide.max.bp* parameter set to 5000. Next, we used the *snpgdsGRM()* function to calculate a GRM based on the Genome-wide Complex Trait Analysis (GCTA) method (Yang *et al.* 2011).

### GWAS-GLM enrichment and directionality

We identified regions of the genome that are enriched for SNPs identified as top hits in the GWAS analysis and the T_max_0–15d model for Charlottesville (Fig. 7b, Supplementary Fig. 13b). First, we partitioned the genome into 8 bins (4 autosomal arms, and partitioning inside or outside inversions) and calculated enrichment and directionality. We report enrichment as the log_2_(odds-ratio) from a FET tabulating the number of SNPs that are among the most significant 5% for each GWAS and the T_max_0–15d environmental GLM model. Next, we calculated the directionality score between our GWAS and GLM models. The directionality score was calculated by comparing the sign of the regression coefficients between the T_max_0–15d GLM model (β_2_(γ); equations 4.1 or 4.2) and the GWAS model (β_2_; equations 8 and 9).

The directionality score was calculated as the proportion of times that the sign of these β terms was equal (conditional on both GLM and GWAS being significant; ranked *P*-value < 5%). The null expectation is 50%. A directionality score of 100% indicates that the sign of allele frequency change with respect to temperature at every SNP under investigation is the same as the sign of allelic effect on trait value. A value of 0% indicates that all SNPs have opposing signs of effect in the GLM and GWAS analysis. Therefore, values of 100 and 0% are equivalent but reflect different predictions of the change in trait value as a function of the environment. We repeated this analysis with 100 permutations of the T_max_0–15d environmental GLM to develop an empirical null distribution for the enrichment and directionality tests.

Next, we sought to identify chromosomal windows that are enriched for SNPs ranked as the top 5% most significant (genome-wide) for both the GLM and GWAS analysis. We did this by conducting a sliding window analysis using a window size of 0.1 Mb and a step size of 50 kb. For each window, and for each phenotype, we tabulated the number of SNPs that are among the most significant (i.e. in the top 5%) for the GWAS and the T_max_0–15d environmental model and conducted a FET (Fig. 7c, Supplementary Fig. 13c). For each window, we counted the number of phenotypes with significant enrichment of SNPs that are both GWAS and GLM outliers (Bonferroni corrected *P-value* < 0.05; see y-axis in Fig. 7c). We performed this same analysis using the 100 sets of permuted T_max_0–15d environmental GLMs. For each window, we tabulated the distribution of the number of phenotypes significantly enriched between the GWAS and the permuted T_max_0–15d GLM. We identified candidate subregions as those where the number of phenotypes that are enriched in the real data exceeds the 95% largest value across the GLM permutations for that window. Because we calculated critical thresholds for each window separately, the significance threshold varies across the genome.

### Startle response quantitative complementation tests

To validate the phenotypic effect of candidate regions linked to the inversion, we focused on 1 candidate phenotype—startle response (Fig. 7e-h, Supplementary Fig. 14). We selected a set of 5 deficiency lines covering regions of interest (see results and Supplementary Table 10). The deficiencies are presumed to be on the standard karyotype, and the deficiency stocks also segregate a multiply inverted balancer chromosome on chromosome 2. We selected DGRP lines that were homozygous for inverted or standard karyotypes of In(2L)t. We constructed 25 F1 crosses between the deficiency stocks and the DGRP lines (see Supplementary Table 10: Crossing Scheme). For example, the Df(2L)BSC37, dpp[EP2232]/CyO deficiency (Bloomington #7144), which spans 2.1 Mb to 2.5 Mb of 2L and covers the distal breakpoint of In(2L)t, was crossed with 3 inverted and 2 standard DGRP lines. For each F1 cross, we sorted 3–5 day-old females into balancer and deficiency F1 backgrounds based on the curly wings phenotype. For these crosses, we assessed startle response phenotypes using a Trikinetic monitor (DAM2 *Drosophila* Activity Monitor) assay. Additional details about this assay and analysis are in Supplementary Methods 2.

## Results

### An expanding resource for *Drosophila* population genomics

To study the temporal dynamics of drift and selection, we generated pool-seq (Schlötterer *et al.* 2014) and individual resequencing data from a dense temporal sampling of *D. melanogaster* in Charlottesville, VA. We combined the Charlottesville data with the DEST dataset. This dataset (Supplementary Table 1) is a growing resource of *Drosophila* population genomic data from flies collected throughout the year over multiple years in over 100 localities, across various continents. Using the combined pool-seq DEST dataset, we identified 3,866,555 autosomal SNPs that passed filtering. For the individual-based sequencing, we identified 6,689,236 autosomal SNPs that passed filtering.

### Fly populations are structured in both space and time

We used PCA to identify patterns of population structure in our samples. We used common SNPs with a minimum allele frequency of 1% across populations (432,407 SNPs for the PCA; 2L = 117,076; 2R = 97002; 3L = 104,582; 3R = 113,747). We focused on localities where flies were sampled at multiple points in time over multiple years from Charlottesville and DEST (i.e. Germany, Munich, and Broggingen; Türkiye, Yesiloz; Ukraine, Odessa; Finland, Akaa; USA, PA, Linvilla and WI, Cross Plains). Consistent with previous analyses (Kapun *et al.* 2020, 2021), PC1 separates samples from Europe and North America (Fig. 1a; Latitude: *F_1,86_* = 586.13, *P*-value = 3.67 × 10^−40^; Longitude: *F_1,86_* = 605.47, *P*-value = 1.08 × 10^−40^) whereas PC2 separates the eastern and western phylogeographic clusters in Europe (Latitude: *F_1,35_* = 0.019, *P*-value = 0.89; Longitude: *F_1,35_* = 8.32, *P*-value = 0.0066). Samples that were collected at the same locality cluster together, yet these populations also show signals of genetic change from 1 year to the next (Supplementary Table 2). The overall pattern of year-to-year temporal structure can be visualized as a vector formed among samples collected in subsequent years (Fig. 1a and b and Supplementary Fig. 1). To determine whether this ordination is driven by the influence of chromosomal inversions and coding regions, we repeated the PCA by excluding all SNPs inside inversions as well as those in protein-coding regions. This additional filtering step reduces our SNP count from 432,407 to 60,940 (2L = 17,311; 2R = 21,955; 3L = 11,756; 3R = 9,918). The analysis shows a strong correlation between the ordination patterns (i.e. sample coordinates in PC space) between the filtered and unfiltered PCA (PC1 corr. = −0.998, *P*-value = 5.53 × 10^−117^; PC2 corr. = 0.962, *P*-value = 5.539 × 10^−51^; PC3 corr. = 0.924, *P*-value = 3.97 × 10^−38^). These results suggest that the patterns of spatiotemporal population structure capture in the 3 main PCs are robust to inversions and coding-region-SNPs across the genome.

Next, we assessed the stability of spatial structure through time. We did this by comparing the relationship between genetic differentiation (*F*_ST_) and spatial distance measured as the haversine distance between 2 localities (*d*_ha_) for populations sampled, at least once, in years 2014, 2015, and 2016 in the DEST dataset (European populations only). These years and samples were chosen as they have the highest number of comparisons arising from the exact same place across those 3 years (273 comparisons; samples from Germany, France, Switzerland, Ukraine, and the United Kingdom). Under a model where populations are stable over time, we expect to see a correlation among pairwise *F*_ST_ across years. This is because demes do not go locally extinct over the winter and the specific patterns of genetic differentiation among localities are preserved from 1 year to the next. Under an alternative scenario where populations are locally extirpated, we expect uncorrelated patterns of *F*_ST_ from year to year. This is because demes that go locally extinct will be recolonized from far away refugia that are likely to be different from 1 year to the next. Calculating the pairwise correlation of *F*_ST_ among years suggests that spatial structure is temporally stable in *D. melanogaster* (Fig. 1c-e; 2014–2015 corr. = 0.89, *P*-value = 2.20 × 10^−16^; 2015–2016 corr. = 0.92, *P*-value = 2.20 × 10^−16^; 2014–2016 corr. = 0.96, *P*-value = 2.20 × 10^−16^).

### Temporal structure is driven by seasonal boom-bust demography

To test the hypothesis that seasonal fluctuations in population size influence the genetic composition of populations, we compared patterns of *F*_ST_ between samples collected within a growing season relative to *F*_ST_ between samples separated by winter. We conducted this analysis on localities where flies were sampled at multiple points in time over multiple years, i.e. those used in the PCA of Fig. 1a and b. The amount of genetic differentiation accrued within the growing season is smaller than that accrued overwinter (Fig. 1f; Supplementary Table 3; median within-year *F*_ST_ = 0.0031, median overwinter *F*_ST_ = 0.0045). Genetic differentiation within localities through time is observed across multiple years and the rate of increase of genetic differentiation varies among populations (Fig. 1g; Analysis of Covariance [ANCOVA], year effect: *F_1,720_* = 5.40, *P*-value = 0.02; year × pop effect: *F_7,720_* = 16.71, *P* = 2.10 × 10^−16^). The signal of year-to-year allele frequency change is distributed across the genome, consistent with a demographic explanation. To show this, we repeated the PCA using random subsamples of SNPs across autosomes. We then ran correlation analyses of PC projections relative to the year of collection. Our results show that the correlation of PCs 1–3 with year is robust in sample sets larger than 1,000 loci demonstrating that the demographic signal is spread throughout the genome (Supplementary Figs. 2 and 3; Supplementary Table 4).

To quantify the general strength of a winter bottleneck, we conducted forward genetic simulations designed to emulate the boom-bust cycle and sampling scheme for the Charlottesville samples (see Supplementary Fig. 4). We simulated 50 generations (∼3 years) of a population with similar genetic properties as *D. melanogaster.* We subjected these populations to yearly cycles of population size change of variable magnitude (booms-and-busts), as well as a null model of constant population size. We calculated a variety of summary statistics (see Materials and Methods; Supplementary Fig. 4) and used ABC to determine the set of parameters that most closely fit the real data. Our results provide support for the hypothesis of yearly population expansions and contractions and suggest that the magnitude of winter collapse, in Charlottesville, is on the order of 98% of the maximum summer size (median *N_Min_* = 283 [97.5% CI: 260; 406], median *N_Max_* = 27,584 [13,217; 46,746], median *N*_e_ [effective population size, i.e. the harmonic mean of *N*] = 2,234 [1,926; 3,240]).

### In(2L)t shows signatures of adaptive tracking and footprints of natural selection

While projections at PCs 1 and 2 of most chromosome arms are primarily explained by year of collection (Fig. 2a), projections at 2L appear to be driven by the frequencies of the cosmopolitan inversion In(2L)t (Figs. 2b, Supplementary Data S2 and S3; also Supplementary Fig. 5 and Table 4). This observation suggests that natural selection may be acting on the 10Mb inversion.

To identify signatures of adaptive tracking, we modeled allele frequency change through time in Charlottesville using a GLM. We modeled allele frequency at each SNP in the genome as a function of the year of collection and an aspect of the environment prior to collection. We tested 100 environmental variables that summarize temperature, precipitation, and humidity in the weeks prior to sampling (Supplementary Fig. 6). For each SNP, we assessed which of these environmental models is the best model more often than expected from the permutation of the environmental labels. For SNPs inside In(2L)t, 2 models emerge as the best-fit models: the maximum temperature 7–15 days prior to collection and the maximum temperature 0–15 days prior to collection. These models are, respectively, 5.2 and 5.1 times more likely to be observed as the best model in the real data relative to the permutations of the environmental GLM models (Fig. 2c). The SNP-wise *P*-values of these models are highly correlated (corr = 0.8, *P*-value = 2.10 × 10^−16^). Given that they are strongly correlated and proxies for each other, we decided to focus downstream analyses on the maximum temperature 0–15d model as this model encompasses a broader time window and represents a full generation in flies (see info for all models in Supplementary Data 1, and 2). For simplicity, we refer to this model as “T_max_0–15d.”

We summarized the output of the T_max_0–15d model using sliding window approaches that test if SNPs whose frequency is strongly correlated with T_max_0–15d are randomly distributed throughout the genome. We calculated 2 summary statistics on our pooled data. First, we tested whether windows across the genome are enriched for the smallest 5% of the GLM *P*-values (signal enrichment test, Fig. 2c). Second, we implemented the WZA *P*-value aggregation test (aggregation test, Fig. 3a). In both cases, we assessed statistical significance by comparing sliding window results from the real data to the sliding window analysis of the 100 random permutations of the T_max_0–15d variable (Fig. 2d). We show that there is an enrichment of SNPs whose AF are correlated to T_max_0–15d within and around In(2L)t but not for other regions of the genome (Fig. 2e). By comparing the results of these tests with the T_max_0–15d environmental GLM permutations, we highlight 6 candidate loci within In(2L)t centered at 3.1, 4.7, 5.2, 6.1, 6.8, and 9.6 Mbs that outperform permutations and are enriched for SNPs whose frequency is strongly correlated with recent maximum temperature (Fig. 3a). We used these peaks to define windows (“w”; e.g. w3.1, w4.7, *etc*.) of interest that spans +/− 0.2Mb to the left and right of the maximum peak of the signal.

To test whether SNPs associated with T_max_0–15d are more differentiated than expected based on short-term demographic fluctuations, we compared our GLM result for the T_max_0–15d GLM model to the output of *BayPass* (Gautier 2015; Olazcuaga *et al.* 2022). First, we asked whether the standardized measure of genetic differentiation that corrects for population structure, XtX_ST_, is elevated on chromosome 2L, and inside the inversion relative to the rest of the genome. We find that the In(2L)t region has the highest density of XtX_ST_ outliers (*q* < 0.05) across the genome: 23.7% of XtX_ST_ outliers are within In(2L)t, a locus that occupies roughly 10% of the genome, whereas XtX_ST_ outliers are less abundant in other regions of the genome (Supplementary Fig. 7a). We also assessed whether the top 5% T_max_0–15d GLM sites are enriched for XtX_ST_ outliers, compared to the top 5% of SNPs from the GLM permutations. We find that top GLM outliers are only enriched for XtX_ST_ outliers on chromosome 2L (Fig. 3b, Supplementary Fig. 7b), and that this enrichment beats 95% of enrichment statistics calculated from the permuted GLM. This result shows that GLM SNPs are more differentiated through time than expected after accounting for population demography. Next, we used the T_max_0–15d environmental values to conduct a gene-environment association analysis using the standard covariate model in *BayPass*. We generated a simulated neutral distribution of the environmental association using the POD method. The upper 99.9% value of the BF value (on a decibel scale) from the simulations is 6, and we use this value as a threshold for considering a site as significantly associated with T_max_0–15d using the *BayPass* analysis. We find that 57.8% BF outliers are found inside In(2L)t, and that this genomic region contains the highest density of BF outliers across the genome (Supplementary Fig. 7c). Notably, 100% of BF outliers are in the top 5% of T_max_0–15d GLM, far exceeding enrichment values calculated from GLM permutations (Supplementary Fig. 7b).

The localized enrichment of outlier loci suggests a complex haplotype structure at In(2L)t. To examine this haplotype structure, we calculated pairwise LD among SNPs using individually sequenced and phased fly genome data. We estimated the mean linkage (measured as *r*^2^) among all pairwise SNPs in 2L using a sliding window approach (size = 50 Kbp, step = 10 Kbp). SNPs within the windows of interest show high LD across long distances (Fig. 3c). The highest mean pairwise *r*^2^ observed occurs among the windows that contain both left and right In(2L)t breakpoints (mean *r*^2^ = 0.075). The mean *r*^2^ observed between the breakpoints and the windows of interest is 0.057 and the mean *r*^2^ observed among the windows themselves is 0.050. For comparison, the mean *r*^2^ for other SNPs is 0.022 and 0.013 for loci inside and outside the inversion, respectively. To further explore this signal, we also estimated *r*^2^ between individual SNPs and inverted/standard karyotype as inferred by the SVM model (see Supplementary Fig. 5). Windows of interest have elevated mean LD to the inversion (Fig. 3c-inset; e.g. mean *r*^2^ of w5.2 = 0.11, w6.1 = 0.09, w9.6 = 0.07; mean outside of windows = 0.04) even compared with regions immediately adjacent to the inversion breakpoints (Left = 0.09, Right = 0.08). We also estimated the number of SNPs across 2L with “perfect” and “high” levels of LD to the inversion. In this context, perfect LD is *r*^2^ > 0.99 and high LD is *r*^2^ > 0.70. Other than the breakpoints, few regions have SNPs in perfect LD to the inversion (Supplementary Fig. 8a). Of the windows of interest, only w5.2 harbors just 1 SNP with perfect LD (2L:5,155,959; an intronic variant at the *Msp300* gene). When assessing SNPs in high LD, we observe that the windows of interest, particularly w5.2, w6.1, and w9.6, harbor large numbers of SNPs with *r*^2^ > 0.70 (Supplementary Fig. 8b; 119, 357, and 109, respectively). For reference, the mean number of high LD SNPs in comparably sized windows is 24. These findings showcase that while there is high LD among the windows of interest and the inversion breakpoints, In(2L)t is not behaving like a fully linked, 12 Mb locus.

We leverage these regions of high LD to characterize the seasonal change in haplotype frequency. We identified sets of “anchor loci” based on LD and GLM scores (Supplementary Data 3) to represent the major haplotypes at each of our regions of interest, including loci with low GLM *P-*values and high LD with inversion breakpoints. Using these data, we show that the standard karyotype has its lowest frequency in midsummer and highest frequency in the spring and late fall (Fig. 3d). Regressing the average allele frequency at the anchor SNPs against T_max_0–15d shows a significant negative relationship between In(2L)t haplotype frequency and maximum temperature across 2016–2018 (Fig. 3e). These analyses also show that the inverted haplotype is at a higher frequency in spring and late winter compared to summer and fall.

### Footprints of selection at outlier windows

Taken together, the patterns of LD, temporal *F*_ST_, and association with T_max_0–15d, suggest that the outlier windows identified here represent candidate loci under seasonal selection. To further evaluate these signals of selection, we calculated a variety of population genetic statistics at each window to assess the types of evolutionary processes that may be at play. First, we used the individual-based sequencing data to visualize these patterns of haplotype diversity within each window of interest. For this analysis, we combined our individual Charlottesville data with genome sequence data from inbred lines or haploid embryos established from worldwide collections (Supplementary Tables 6 and 7) and show reduced genetic diversity (π), Tajima's D, and haplotype diversity at w6.1 and w9.6 (Fig. 4a and b, Supplementary Figs. 9a-e, 10a and b, and Table 6) consistent with partial selective sweeps (Simonsen *et al.* 1995). Of these, w9.6 is interesting because it colocalizes with a soft sweep identified by Garud *et al.* (2015) in a North American population. We estimated levels of *F_ST_* between standard and inverted karyotypes using our individual sequencing data of flies from Charlottesville. We observe high levels of differentiation around the breakpoints, a pattern that is expected of inversions (Kapun and Flatt 2019). We also observe 2 regions within the inversion that show elevated differentiation (*F*_ST_ > 0.4). One of these regions corresponds to w5.2, a window that primarily encodes the *Msp300* gene (Fig. 4c). Elevated *F_ST_* at this region is also observed in the DGRP, but not in Africa (Supplementary Fig. 10c). The second region of elevated differentiation occurs in w6.1-w6.8. This pattern is only seen in Virginia but not in the DGRP. Lastly, we estimated allele ages of SNPs in the inversion as well as within each inversion haplotype (standard and inverted). Consistent with previous literature estimates, our allele age estimates showcase that In(2L)t is a relatively young inversion that arose ∼85,000 y. ago (Fig. 4d; median TMRCA; current estimates = 75,000–160,000 y; Andolfatto *et al.* 1999; Corbett-Detig and Hartl 2012) and that the mean age of loci inside windows of interest predates this estimate (Supplementary Fig. 8e). We also note that w6.8 harbors young alleles within the inverted karyotype, a signature that is consistent with an intrakaryotype incomplete sweep.

### A trans-species SNP in *Msp300* is highly differentiated in In(2L)t

**Fig. 5.**
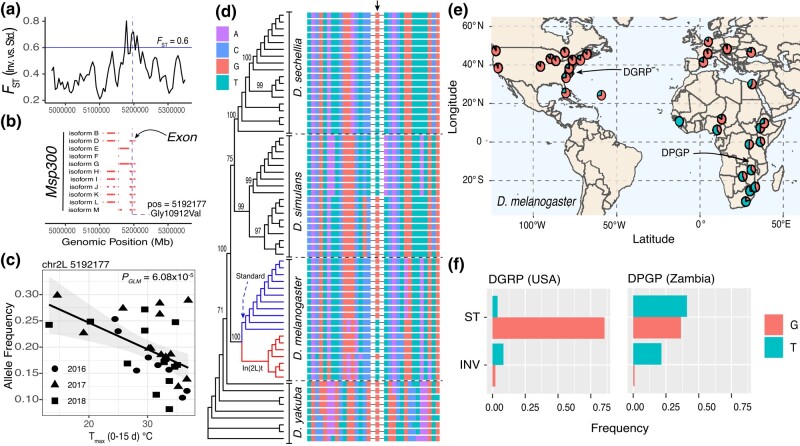
Trans-species polymorphism in *Msp300*. a) Mean *F*_ST_ between inverted and standard karyotype classes, at the *Msp300* region in 2L (Window = 5,000 bp, Step = 1,000 bp; a ∼0.4 Mb region around 32735G > T is shown in chromosome 2L). b) Gene structure and isoforms of *Msp300.* The vertical line indicates the focal mutation (32735G > T, Gly10912Val). c) Correlation between T_max_ (0–15 d) and the frequency of 32735G > T. d) Phylogenetic tree showing the trans-species polymorphism at 32735G > T (*D. melanogaster* samples from North America are shown). Bootstrap values are shown in the nodes. e) AF of 32735G > T across populations of *D. melanogaster*. The location of 2 reference panels, DGRP and DPGP, are shown. f) Frequency of inverted or standard karyotypes carrying 32735G > T in the DGRP and DPGP.

Within w5.2, 1 seasonal SNP in the *Msp300* gene is a trans-specific polymorphism (Fig. 5a; 2L:5192177; c.32735G > T) observed in related *Drosophila* species such as *D. simulans* and *D. sechellia,* but not *D. mauritiana* nor *D. yakuba* (Fig. 5d). This mutation causes a nonsynonymous change (p.Gly10912Val) in the protein (Fig. 5b). The locus is in strong linkage with In(2L)t in Virginia (LD of 2L:5192177 to the inversion is *r*^2^ = 0.68). AF at this locus are strongly correlated with the T_max_0–15d model (*P-*value = 6.05 × 10^−5^; Fig. 5c). To understand how linkage patterns vary across space, we compared the levels of association of each allele of 2L:5192177 (i.e. G and T) with inverted and standard karyotypes in African and European populations. In Europe and North America, G is more abundant on standard backgrounds whereas T is more abundant on inverted backgrounds (FET, odds ratio = 0.013 [95% Confidence Interval (C.I.) = 0.002–0.053]; *P-*value = 2.16 × 10^−13^). In an ancestral African population, the T allele is more abundant relative to G (Fig. 5e; odds ratio = 18.43 [95% C.I. = 4.51–162.6]; *P-*value = 3.288 × 10^−8^) and, unlike temperate populations, both alleles are abundant in the standard karyotype (Fig. 5f).

### Signals of adaptive tracking within In(2L)t are generalizable to other populations

**Fig. 6.**
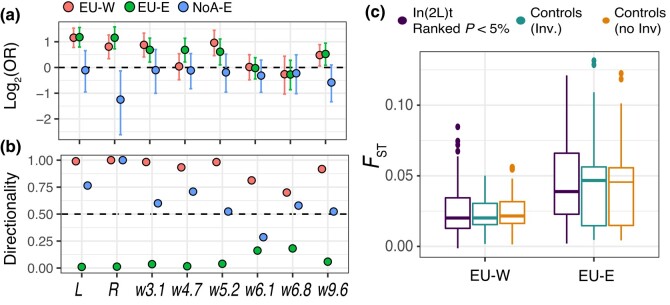
Enrichment, directionality, and genetic differentiation in In(2L)t. a) Enrichment scores (odds ratio [OR]) between top SNPs the T_max_0–15d model in Charlottesville and the best models across other populations at inversion breakpoints and windows in In(2L)t. 95% confidence intervals of the odds ratio are shown. The horizontal line is the null expectation of no enrichment. b) Directionality scores between loci in the T_max_0–15d model in Charlottesville and the best models across other populations at windows of interest and In(2L)t. Directionality is the probability that frequencies change in the same direction across both models. c) Spatial *F*_ST_ for 3 types of markers: candidate SNPs in In(2L)t (ranked *P* < 5% in their respective best models), and controls inside and outside inversions in EU-W and EU-E. The comparisons were done within the EU-W and EU-E clusters separately.

We tested if the associations between environmental variables and In(2L)t observed in Charlottesville are generalizable to other localities. We used linear modeling to determine the most likely environmental correlates of allele frequency change using temporal samples from localities in 3 distinct phylogeographic regions: Europe West, Europe East, and the East Coast of North America. The best-fit models for these regions are distinct from those of Charlottesville (see Supplementary Fig. 11): the variance of humidity in the 0–30 days prior to sampling for EU-E (H_var_0–30d; 22.3 times higher than H_var_0–30d GLM permutations), average humidity 0–60 days prior for EU-W (H_ave_0–60d; 38.2 times higher than H_ave_0–60d GLM permutations), and variance of temperature 30–60 days prior for NoA-E (T_ave_30–60d; 2.28 times higher than T_ave_30–60d GLM permutations). Although the best-fit environmental models identified in these other regions differ from what we identified in Charlottesville, the loci underlying allele frequency change in these regions could be the same. Indeed, the strongest signals of environmental enrichment that we observe in these other phylogeographic clusters are on 2L, and for loci inside In(2L)t (Supplementary Fig. 12). To test if the same loci change in frequency among these regions, and to test if the direction of allele frequency change is consistent among these regions, we conducted enrichment and directionality tests. Candidate loci at w3.1, w5.2, w9.6 and the inversion breakpoints are enriched for SNPs strongly correlated with the weather in both Charlottesville and either EU-E or EU-W, but not NoA-E (FET, *P*-value < 0.05; Fig. 6a). We observe a lack of enrichment at w6.1 and w6.8 when contrasting Charlottesville to EU-W and EU-E, suggesting that the areas of reduced variation in this region may be private to North American populations. The directionality test shows that nearly all top SNPs that are changing in frequency in Charlottesville also change in frequency in the same direction in EU-E (directionality scores > 90%). The changes are also observed in EU-W, but the direction is anticorrelated (Fig. 6b). This anticorrelation may be driven by the fact that the best model in EU-W is driven by weather occurring 3 months in the past and thus alleles are correlated to changes in entirely different seasons. Notably, no significant directionality relationships are observed in NoA-E. Finally, to assess if In(2L)t or candidate loci are spatially differentiated, we calculated *F*_ST_ between locales as a function of their phylogeographic cluster relative to matched controls. Pairwise *F*_ST_ shows no difference among In(2L)t outliers and control loci across space (Kruskal-Wallis tests, *P*-value _EU-E_ = 0.47, *P*-value _EU-E_ = 0.46; Fig. 6c).

### In(2L)t SNPs are associated with ecologically important traits

**Fig. 7.**
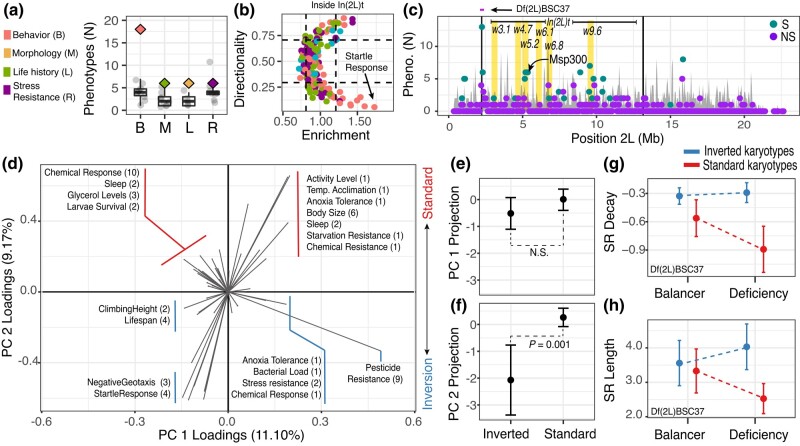
Phenotypes associated with candidate loci on chromosome arm 2l. a) The number of GWAS phenotypes associated with inversion status, In(2L)t, in the DGRP. Traits are divided into 4 phenotypic categories. The real data are shown as diamonds, GWAS permutations are shown as black points and boxplots. b) Directionality and enrichment analysis between the DGRP-GWAS and the best environmental model in Charlottesville along for In(2L)t 2L. Black dashed lines indicate null expectations estimated as the 5 and 95% percentiles calculated using the T_max_0–15d GLM permutations. Each point is a phenotype and colored as in a. c) Window level enrichment analysis across 2L. The y-axis shows the number of phenotypic measurements associated with SNPs that are both outliers in the GWAS and the GLM analysis. Windows that exceed 100 permutations of the T_max_0–15d GLM are shown in turquoise, otherwise in purple. Vertical lines show the boundaries of In(2L)t. d) PCA constructed using phenotypic values of traits that show enrichment of GWAS and GLM SNPs in the sliding window analysis. Each arrow represents a phenotype characterized in a GWAS study. The number of studies measuring the same phenotype is shown in parentheses. e) Inversion status is significantly associated with the phenotype PC1. f) Inversion status is not associated with PC2. For e and f, the 95% confidence intervals are shown. g) Quantitative complementation tests using deficiencies show that the inverted and standard karyotypes have significantly different effects in the deficiency background but not the balancer background for the decay rate of startle response. h) Same as g but for the startle response length.

To elucidate the phenotypic consequences of the candidate loci we identified, we aggregated line mean estimates of the DGRP for 225 phenotypic measurements collected by dozens of labs (Supplementary Tables 8 and 9). The phenotypic variation of 36 traits is correlated with In(2L)t inversion status, and these traits span all phenotypic classifications (Fig. 7a). However, this signal of association is not observed in other chromosomes or other inverted regions (Supplementary Fig. 13a).

We performed GWAS for each of the 225 phenotypic measurements and assessed the level of enrichment between loci that are associated with each measurement and loci that are strongly associated with the T_max_0–15d model in Charlottesville. We show that only In(2L)t is enriched for loci that are both associated with phenotypic variation in the DGRP and also correlated with T_max_0–15d in Charlottesville (Fig. 7b and Supplementary Fig. 13b). We also investigated the proportion of SNPs that have the same sign of allele frequency change conditional on those SNPs being in the top 5% of both the GWAS and the GLM models (i.e. “directionality”; Fig. 7b). For each SNP under investigation, we used the estimated allelic effect from the GWAS and the slope of allele frequency change with respect to the T_max_0–15d model. Like our previous directionality analysis, our null hypothesis is 50%. Values different from 50% show evidence of consistent alignment of effect directions between the GWAS and GLM analyses. SNPs on 2L that are associated with phenotypes and T_max_0–15d show levels of directionality greater than we expect compared to the T_max_0–15d GLM permutations (Fig. 7b and Supplementary Fig. 13b).

We performed a sliding window analysis across 2L to identify subregions that are especially enriched for SNPs that are top hits for both GWAS of each phenotypic measurement and the T_max_0–15d GLM relative to the T_max_0–15d GLM permutations (Fig. 7c). For any window, and for any phenotypic measurement, we calculated the odds ratio that SNPs inside the window are in the top 5% for GWAS and GLM, ranked genome-wide. We identified windows with significant enrichment after correcting for multiple testing and tabulated the number of phenotypic measurements with significant enrichment for each window. We find that regions inside the inversion are significantly enriched for GWAS and GLM outliers for 57 phenotypic measurements.

The inverted and standard alleles impact a suite of traits, demonstrating pleiotropy and suggesting covariance. To characterize patterns of trait covariation, we conducted PCA using the 57 phenotypic measurements identified in our sliding window analysis (Fig. 7d). Presence of the inversion is significantly associated with PC2 (*t-test*, *t =* 3.6365, df = 19.76, *P-*value = 0.001; Fig. 7f), but not PC1 (*t =* 1.4792, df = 37.862, *P-*value = 0.14; Fig. 7e) or PC3 (*t* = −0.14899, df = 20.776, *P-*value = 0.883). Based on these analyses, we identify groups of phenotypes associated with inversion homozygotes such as higher levels of basal and induced activity, lifespan, and resistance to various stressors including pesticides. On the other hand, the phenotypes associated with the standard homozygotes are characterized by higher values for sleep, starvation resistance, and chemical resistance.

To validate the phenotypic effect of allelic variation at the candidate regions, we focused on startle response. The startle response trait significantly varies as a function of inversion presence (Fig. 7a), is a top hit in our GLM-GWAS enrichment analyses (Fig. 7b), and inverted homozygotes have a greater startle response than standard homozygotes. We used quantitative complementation to validate the effect of candidate windows on startle response. We crossed selected DGRP lines to 5 deficiency-bearing lines for regions in In(2L)t (Supplementary Table 10 and Fig. 14). One deficiency that covers the left-inversion breakpoint (2.17–2.45 Mb; Fig. 7c, top) fails to complement the inverted and standard alleles for 2 measures of startle response: the rate of return to basal activity (Fig. 7g; *χ*^2^_SR decay_[df = 3] = 24.20, *P*-value = 2.26 × 10^−5^) and the startle-response length (Fig. 7h; *χ*^2^_SR length_[df = 1] = 3.504, *P*-value = 0.061; Supplementary Table 11). Complementation tests confirm that the inversion increases startle response, consistent with the direction of effect among inbred DGRP lines.

## Discussion

In this paper, we used genomic data to study the dynamics of allele frequency change across the growing season in *D. melanogaster* with special emphasis on patterns of genetic change in the cosmopolitan inversion In(2L)t. We show that the frequency of In(2L)t fluctuates seasonally, likely as a result of weather in the weeks prior to collection. We identified groups of phenotypes associated with the inverted and standard forms of 2L and validated phenotypic association using deficiency mapping. This work advances our understanding of natural selection in the wild because it highlights the temporal dynamics of allele frequency change in natural systems and provides functional insights into our understanding of adaptive tracking.

### How does seasonal demography influence fluctuating selection?

Our demographic analyses revealed 2 insights into the temporal dynamics of allele frequency change in *D. melanogaster*. First, we show that spatial population structure is stable over time (Fig. 1a-e) suggesting that populations overwinter locally and are not recolonized from distant refugia yearly. Second, we show that local population size contracts during overwintering bottlenecks. We observe that genetic differentiation overwinter is larger than over the growing season for several temperate populations (Fig. 1f). Some populations have much larger levels of overwintering *F*_ST_ than others, a result that may be driven by different strengths of winter bottleneck at these localities (Fig. 1f). Using forward genetic simulations, we show that the short-term effective population size (*N*_E_) of 1 deme (Charlottesville) is between ∼2,000 and 3,000 (Supplementary Fig. 4), consistent with another recent estimate of ∼10,000 (Lange *et al.* 2022). The estimates of local deme size are much smaller than the estimated global census size of 10^8^–10^20^ (Karasov *et al.* 2010; Buffalo 2021), and the long-term *N*_E_ size of 10^6^ (Kapopoulou et al. 2020). Since the efficacy of selection is proportional to 1/*N*_E_, understanding *N*_E_ differences across local, global, and historical contexts is important to contextualize the roles of selection in the wild. While the large census sizes of *D. melanogaster* ensure that it is not mutation-limited (Karasov *et al.* 2010), ultimately selection acts on individuals living and reproducing within finite populations where local demographics have an oversized role (Wright 1931).

### Is In(2L)t an adaptive inversion?

The In(2L)t locus has long been hypothesized to be an adaptive inversion in *D. melanogaster* (Lemeunier and Aulard 1992; reviewed in Kapun and Flatt 2019). Previous work has demonstrated selective sweeps near the proximal (right) breakpoint of In(2L)t (Andolfatto *et al.* 1999; Andolfatto and Kreitman 2000), and some evidence suggests epistatic selection between the proximal breakpoint and the neighboring *Adh* locus that sits just outside of the inversion region (van Delden and Kamping 1991). Yet, despite these studies, the drivers of selection on this inversion are poorly understood. For example, while inversions in *Drosophila* tend to be more common in lower latitudes and thus have been argued to be adapted to warmer environments (Stalker 1980), these predictions do not hold for In(2L)t. A meta-analysis of the inversion's worldwide frequency does not show clear and consistent correlations with latitude although it is found at intermediate frequencies in populations around the world (Kapun and Flatt 2019).

On the specific topic of seasonality, the evidence has been mixed. Early analyses of *D. melanogaster* inversions found little evidence for seasonal oscillations at In(2L)t (Stalker 1980). Later studies investigating the associations between In(2L)t and temperature variables showed positive correlations between temperature and inversion frequencies (van Delden and Kamping 1989; Kamping and Delden 1999). In these cases, the authors observed that flies with In(2L)t showed slower development time at lower temperatures. Likewise, van Delden and Kamping (1997) and van’t Land (1997) observed associations between the inversion and resistance to high temperature and posited that the inversion likely plays a role in the genetic cline of the classic *Adh* allozyme polymorphism. Despite these findings, follow-up studies surveying natural fly populations showed that the In(2L)t inversion increases in frequency in cold weather (Sanchez-Refusta *et al.* 1990). Recent genomic work on samples collected across Europe shows that genetic differentiation surrounding the In(2L)t locus is high in the fall, and low in the spring, suggesting temporally heterogeneous selection (Bogaerts-Márquez *et al.* 2020). Taken together, these results suggest that In(2L)t likely contributes to adaptation to fluctuating environments, and also highlight the gaps in our knowledge about the functional and evolutionary consequences of this cosmopolitan inversion polymorphism.

In recent years, theoretical and empirical work has provided a blueprint to characterize adaptive inversions in nature. Collectively, this work predicts that inversions may capture new variants that drive local adaptation, harbor disproportionate amounts of heritability for ecologically important traits, and contain loci that are pleiotropic (Thompson and Jiggins 2014; Charlesworth 2016; Küpper *et al.* 2016; Hager *et al.* 2022; Schaal *et al.* 2022). Empirical work has shown that many loci classified as adaptive inversions are often genome outliers when comparing among ecotypes (Kirubakaran *et al.* 2016), show clear correlations to ecological stressors (Wellenreuther and Bernatchez 2018), and contain alleles in strong but imperfect linkage disequilibrium with each other and the inversion breakpoints (Schwander *et al.* 2014).

We show that In(2L)t harbors many of these characteristics of an adaptive inversion. For instance, allele frequency fluctuations at In(2L)t in an orchard population are correlated with ecological factors related to weather in Virginia (Figs. 2c-e and 3d-e), and these loci are also enriched for SNPs that fluctuate in response to weather in European populations (Fig. 6a). In general, we observe strong long-distance LD between candidate loci inside In(2L)t (Fig. 3c), consistent with theoretical models that distinguish adaptive and neutral inversions (Kapun and Flatt 2019). We observe that while there is elevated linkage inside the inversion relative to the rest of 2L, loci inside the inversion show varying levels of association with the breakpoints (Fig. 3c, Supplementary Fig. 8a and b). This finding reveals that In(2L)t is not behaving like a single, fully linked, 12 Mb locus. Yet, we also observe that the windows of interest show localized elevation in their levels of linkage to the breakpoints (Fig. 3c-inset) and that patterns of linkage vary among populations (Fig. 5e).

Based on these observations we hypothesize that while the inversion, per se, may not have evolved as an explicitly adaptive locus, the lack of recombination may have helped the formation of a coadapted gene complex that facilitates seasonal adaptation. This hypothesis is consistent with previous work. First, Corbett-Detig and Hartl (2012) observed that In(2L)t's distal breakpoint truncates the 3′ UTR of 1 gene (CG15387), but otherwise, the breakpoints of this inversion do not appear to create null alleles of any kind. Second, the expression of many genes across the genome is associated with In(2L)t, but the effect size of the inversion on gene expression is modest and not biased toward genes near the breakpoint (Lavington and Kern 2017). And third, the generation of a transgenic version of In(2L)t did not cause strong differences in gene expression of neighboring genes (Said *et al.* 2018). These findings, in combination with our evidence, suggest that the adaptive value of In(2L)t may result from a coadapted gene complex that is shielded from recombination by the inversion.

### Does In(2L)t show footprints of adaptive tracking?

The topic of adaptive tracking, particularly in *Drosophila*, has garnered attention and controversy in the literature. While multiple papers have shown that adaptive tracking is a general and quantifiable phenomenon in various *Drosophila* species (Dobzhansky 1943; Dobzhansky 1947; Dobzhansky and Ayala 1973; Boulétreau-Merle *et al.* 1987; Rodríguez-Trelles *et al.* 1996; Rezende *et al.* 2010; Bergland *et al.* 2014; Machado *et al.* 2021; Olazcuaga *et al.* 2022; Rudman *et al.* 2022), alternative hypotheses have been presented. These alternatives include the role of population substructure (Charlesworth and Giesel 1972; Lynch and Ho 2020), microspatial heterogeneity (Barker *et al.* 1986), sampling bias (Buffalo and Coop 2019), or mass migration (Buffalo and Coop 2019, 2020) in determining temporal patterns of genetic variation. For instance, in a recent study, Buffalo and Coop (2020) suggest that signals of temporal allele frequency change reported by Bergland *et al.* (2014) could be driven by nonseasonal temporal structure. They arrived at their conclusion in part because the patterns of autocovariance of allele frequency change through time did not match their expectation, even though the autocovariance terms they calculated were significantly different from zero. However, the autocovariance analysis of Buffalo and Coop (2019, 2020), used to identify linked selection, has notable caveats in its application to temporal allele frequency data of natural populations. For example, the method assumes that the demes under study are of constant population size and isolated. While these assumptions may be reasonable for their model, and applicable to laboratory selection experiments, they are unreasonable for many wild systems including flies in orchards. Our data provides evidence that population sizes in the orchard change between summer and winter (Fig. 1f, Supplementary Fig. 4), and observations from field collections suggest that census size changes throughout the growing season too (Atkinson and Shorrocks 1977; Gleason *et al.* 2019). While we find no evidence of wholesale population turnover of *D. melanogaster* in the populations we study throughout the world (Fig. 1a and b), it is certain that migration is happening to some degree among local demes and that fly populations in orchards are not “closed.” Finally, Buffalo and Coop (2020) suggested that permutation strategies like those we have used here and elsewhere (Machado *et al.* 2021) might not be sufficient to capture the underlying demographic structure of the data, and that models that test for gene-environment association after accounting for sample covariance are more appropriate (Forester *et al.* 2018; Bourgeois and Warren 2021; Luo *et al.* 2021). Yet, there is no guarantee that such models have a low false-positive rate (Lotterhos and Whitlock 2014; Whitlock and Lotterhos 2015; Lotterhos 2023) nor has their performance been evaluated for time-series data. Nonetheless, we have implemented differentiation analysis using *BayPass* (Gautier 2015), and have shown that SNPs inside In(2L)t as well as top T_max_0–15d GLM SNPs have elevated XtX_ST_ values and signals of association with temperature maximum, compared to simulated data that conditions on the observed population covariance matrix (Supplementary Fig. 7). Therefore, we conclude that In(2L)t shows statistical evidence of strong allele frequency change associated with maximum temperatures in the weeks prior to sampling.

As a form of natural selection, adaptive tracking can be difficult to differentiate from other processes that also result in allele frequency oscillations (Grant and Grant 2002; Reimchen and Nosil 2002; Bell 2010; Morrissey and Hadfield 2012; Nosil *et al.* 2018; De Villemereuil *et al.* 2020; Chevin *et al.* 2022). For example, we show that the specific aspects of weather identified by our analysis vary by geographical region (T_max_0–15d in Charlottesville, H_var_0–30d in EU-E, H_ave_0–60d in EU-W, and T_ave_30–60d in NoA-E). Are these aspects of weather the proximate causes of temporally varying selection, or do they reflect something else? We consider 3 nonmutually exclusive hypotheses. First, AF oscillate across seasons as a direct consequence of fluctuating environmental selection. Although the specific environmental models that we identify suggest different stressors drive selection across the species range, these variables may simply be proxies for a shared seasonal stressor. Second, due to the temporal nature of our data it is plausible that AF are driven by negative frequency-dependent selection (Chevin *et al.* 2022), and because weather is seasonal, artefactual associations with environmental variables may have emerged. A third hypothesis is the joint action of genetic overdominance and boom-bust demography. In this model, the inverted and standard karyotypes are maintained via heterotic (Hedrick 2012) or associative overdominance (Ohta 1971) and are kicked out of equilibrium by yearly bottlenecks. As selection returns alleles back to equilibrium frequency, allele trajectories may resemble seasonal oscillations.

Although we cannot rule out any of these models conclusively, our data are most in line with the seasonal stressor hypotheses. To arrive at this conclusion, we first consider the overdominance-perturbation model. Under this model, we predict low spatial differentiation and lower-than-average temporal differentiation because natural selection would rapidly push populations back to a common equilibrium. To the contrary, our data show high temporal differentiation within Charlottesville (Fig. 3d and e), yet only average differentiation across spatial gradients (Fig. 6c). While evidence in favor of the seasonal stressor model over the negative frequency-dependent selection model is more limited, and differentiating these hypotheses is challenging (Chevin *et al.* 2022), several pieces of evidence point in favor of the seasonal stressor model. The first is a comparison between our results and several previous studies. In one, the seasonal frequency change of In(2L)t was documented during the 1980s in a Spanish population (Sanchez-Refusta *et al.* 1990). There, In(2L)t is high frequency in the fall and low frequency in the summer, similar to what we observe (see Supplementary Fig. 15). In(2L)t was also found to be higher frequency in the fall compared to the summer in some midlatitude North American populations (Kapun *et al.* 2016). The second are the signals of concordance that we observe across our environmental analyses in various geographical regions. Loci responding to temperature in Charlottesville are enriched among the best models in EU-W and EU-E (Fig. 6a). We are puzzled by the lack of enrichment or directionality signals with the NoA-E samples, especially since the inversion break point of In(2L)t is enriched in the analysis by Machado *et al.* (2021). Yet, it is possible that due to its low sampling density, the NoA-E dataset may not have enough power when regressed against highly granular temperature changes within a given year. Future work using higher sampling densities across the east coast of North America will allow us to test this hypothesis. Taken together, patterns of spatial and temporal allele frequency change show that In(2L)t is common across the range, weakly differentiated across spatial gradients, and highly differentiated through time, thus suggesting that In(2L)t and loci within it are affected by temporally heterogeneous selection.

### Are seasonal SNPs in In(2L)t old or new mutations?

How adaptive inversions evolve remains an open question (Kapun and Flatt 2019). Of particular interest is whether adaptive inversions emerge as a result of the divergence among old balanced polymorphisms, or whether they emerge as neutral structural variants and subsequently accumulate advantageous mutations (Schaal *et al.* 2022). Our results provide insights into this matter. While In(2L)t contains several old seasonal loci that predate the inversion, we also show that this inversion may still be accumulating beneficial alleles. In particular, we observe that the candidate window w9.6 colocalizes with a soft-sweep identified in North America that appears to be absent in an African population (Garud *et al.* 2015, 2021; Garud and Petrov 2016). Interestingly, the region corresponding to w6.1/w6.8 shows a similar reduction of genetic variation (Fig. 4b). Taken together, these signals suggest that these regions may also have experienced selective sweeps targeting alleles on the inverted karyotype. Yet, given the history of recent population expansion in *D. melanogaster* (Stephan and Li 2007), depressed values of Tajima's D and genetic variation may be due to nonequilibrium demography (Nielsen 2001). Differentiating between these adaptive and demographic hypotheses will require functional validation of the putative drivers of the sweeps (e.g. Glaser-Schmitt *et al.* 2023). Functional evidence will be important for testing whether seasonal adaptation in In(2L)t is “fine-tuned” by young, habitat-specific alleles. Nevertheless, our work suggests that: (1) both old as well as new mutations may play important roles in the evolution of In(2L)t and that, (2) both balancing and directional selection appear to be acting at different levels of genomic organization (between vs within inversion karyotypes).

### What are the candidate phenotypes and candidate loci for seasonality?

Although individual candidate loci underlying seasonal evolution in *D. melanogaster* have been identified and validated (Schmidt *et al.* 2008; Paaby *et al.* 2014; Cogni *et al.* 2015; Behrman *et al.* 2018; Glaser-Schmitt *et al.* 2021) genome-wide analysis of seasonal allele frequency change in this species has provided limited resolution to identify targets underlying adaptive tracking (Bergland *et al.* 2014; Machado *et al.* 2021). The dense temporal sampling that we employ here may have helped resolve this limitation and has identified signals surrounding a handful of candidate loci associated with In(2L)t (Fig. 3a) and linked these regions to phenotype (Fig. 7). We show that standard karyotype homozygotes are associated with higher values for sleep, starvation resistance, and chemical resistance. The inverted homozygote, on the other hand, is associated with higher levels of activity, lifespan, and negative geotaxis. It is important to emphasize that our findings do not imply that seasonal selection is acting on all of these traits independently since many traits are correlated with each other (Fig. 7d). Here, we focused our validation efforts on startle response because it shows high levels of heritability in the DGRP (∼40%; Jordan *et al.* 2012; Xue *et al.* 2017), and shows the strongest signals of enrichment between GWAS hits and GLM hits. We hypothesize that startle response may be an important phenotype for overwintering survival and recolonization. For instance, a faster startle response could increase the chance of finding shelter during the winter and patch recolonization during the summer.

Our work identifies the inversion breakpoints as well as a handful of regions inside the inversion as potential candidate loci for seasonality. One of these regions that particularly stands out is w5.2, the window with the largest *F*_ST_ differentiation among the karyotype classes other than the breakpoints (Figs. 4c and 5a). This window primarily encodes *Msp300,* a nesprin-like protein that mediates the positioning of nuclei, mitochondria, and synaptic junctions in muscle (Rosenberg-Hasson *et al.* 1996; Yu *et al.* 2006; Xie and Fischer 2008; Elhanany-Tamir *et al.* 2012). *Msp300* harbors a trans-species polymorphism (32735G > T) in high LD to the inversion (*r*^2^ = 0.68), and is also polymorphic in 2 closely related taxa (Fig. 5d). It remains unclear whether this trans-specific mutation is a case of ancient balancing selection (Gao *et al.* 2015), or recurrent mutation across the 3 species (Unckless *et al.* 2016). The functional role of 32735G > T is unknown in *D. simulans* and *D. sechellia,* however, the presence of this mutation in *D. sechellia*–a species that is endemic to a tropical island chain–suggests that it might be involved in a more general response to environmental fluctuations.

## Conclusions

### In(2L)t plays a key role in seasonal adaptation

Here we provide evidence showing that In(2L)t experiences strong seasonal selection in *Drosophila* despite strong overwintering drift. In North America, the inversion appears to evolve by adaptively tracking in response to weather weeks prior to collection. We observe the action of both balancing and directional selection at different levels of genomic organization between vs within inversion karyotypes. By showing that seasonal loci in In(2L)t are both young and old, our findings showcase that adaptive inversions that evolve by capturing old beneficial alleles often continue to accumulate adaptive mutation on existing inversion karyotypes (Kirkpatrick and Barton 2006). This exemplifies instances of the action of ancient balancing selection across large spatial scales, and fine-tuning local adaptation within spatially structured populations (also see Kapun *et al.* 2023). Furthermore, our work infers and experimentally validates the phenotypic effects of alternate alleles at a candidate locus linked to In(2L)t. Overall, this work is an example of evolution in action and provides new insights into the biology of adaptive cosmopolitan inversions.

## Supplementary Material

iyad207_Supplementary_Data

## Data Availability

This paper used multiple datasets that are available in the National Center for Biotechnology Information (NCBI; https://www.ncbi.nlm.nih.gov), as described in their corresponding citations. Data generated as part of this paper is also available in NCBI in bioprojects: PRJNA882135, PRJNA728438, and PRJNA727484. Sequence Read Archive (SRA) IDs for individual samples can be found in Supplementary Table 1. A GitHub repository with code can be found at https://github.com/Jcbnunez/Cville-Seasonality-2016-2019. Data S1, S2, and S3 can be found in Zenodo (https://zenodo.org/) at https://zenodo.org/doi/10.5281/zenodo.7305042. Supplemental material available at GENETICS online.

## References

[iyad207-B1] Albers PK, McVean G. 2020. Dating genomic variants and shared ancestry in population-scale sequencing data. PLoS Biol. 18(1):e3000586. doi:10.1371/journal.pbio.3000586. 31951611 PMC6992231

[iyad207-B2] Andolfatto P, Kreitman M. 2000. Molecular variation at the In(2L)t proximal breakpoint site in natural populations of *Drosophila melanogaster* and *D. simulans*. Genetics. 154(4):1681–1691. doi:10.1093/genetics/154.4.1681. 10747062 PMC1461028

[iyad207-B3] Andolfatto P, Wall JD, Kreitman M. 1999. Unusual haplotype structure at the proximal breakpoint of In(2L)t in a natural population of *Drosophila melanogaster*. Genetics. 153(3):1297–1311. doi:10.1093/genetics/153.3.1297. 10545460 PMC1460810

[iyad207-B4] Atkinson W, Shorrocks B. 1977. Breeding site specificity in the domestic Species of *Drosophila*. Oecologia. 29(3):223–232. doi:10.1007/BF00345697. 28309117

[iyad207-B5] Barker JSF, East PD, Weir BS. 1986. Temporal and microgeographic variation in allozyme frequencies in a natural population of *Drosophila buzzatii*. Genetics. 112(3):577–611. doi:10.1093/genetics/112.3.577. 3957005 PMC1202765

[iyad207-B6] Baumdicker F, Bisschop G, Goldstein D, Gower G, Ragsdale AP, Tsambos G, Zhu S, Eldon B, Ellerman EC, Galloway JG, et al 2022. Efficient ancestry and mutation simulation with msprime 1.0. Genetics. 220(3):iyab229. doi:10.1093/genetics/iyab229. 34897427 PMC9176297

[iyad207-B7] Baym M, Kryazhimskiy S, Lieberman TD, Chung H, Desai MM, Kishony R. 2015. Inexpensive multiplexed library preparation for megabase-sized genomes. PLoS One. 10(5):e0128036. doi:10.1371/journal.pone.0128036. 26000737 PMC4441430

[iyad207-B8] Behrman EL, Howick VM, Kapun M, Staubach F, Bergland AO, Petrov DA, Lazzaro BP, Schmidt PS. 2018. Rapid seasonal evolution in innate immunity of wild *Drosophila melanogaster*. Proc R Soc B Biol Sci. 285(1870):20172599. doi:10.1098/rspb.2017.2599. PMC5784205 29321302

[iyad207-B9] Behrman EL, Watson SS, O’Brien KR, Heschel MS, Schmidt PS. 2015. Seasonal variation in life history traits in two *Drosophila* species. J Evol Biol 28(9):1691–1704. doi:10.1111/jeb.12690. 26174167 PMC5089932

[iyad207-B10] Bell G . 2010. Fluctuating selection: the perpetual renewal of adaptation in variable environments. Philos Trans R Soc B Biol Sci. 365(1537):87–97. doi:10.1098/rstb.2009.0150. PMC2842698 20008388

[iyad207-B11] Bergland AO, Behrman EL, O’Brien KR, Schmidt PS, Petrov DA. 2014. Genomic evidence of rapid and stable adaptive oscillations over seasonal time scales in *Drosophila*. PLoS Genet. 10(11):e1004775. doi:10.1371/journal.pgen.1004775. 25375361 PMC4222749

[iyad207-B12] Bertram J, Masel J. 2019. Different mechanisms drive the maintenance of polymorphism at loci subject to strong versus weak fluctuating selection. Evolution. 73(5):883–896. doi:10.1111/evo.13719. 30883731

[iyad207-B13] Biémont C . 1985. Effects of winter on genetic structure of a natural population of *Drosophila melanogaster*. Genet Sel Evol. 17(1):25. doi:10.1186/1297-9686-17-1-25. PMC2713908 22879184

[iyad207-B14] Bogaerts-Márquez M, Guirao-Rico S, Gautier M, González J. 2020. Temperature, rainfall and wind variables underlie environmental adaptation in natural populations of *Drosophila melanogaster*. Mol Ecol. 30(4):938–954. doi:10.1111/mec.15783. PMC7986194 33350518

[iyad207-B15] Booker TR, Yeaman S, Whiting JR, Whitlock MC. 2023. The WZA : a window-based method for characterizing genotype–environment associations. Mol Ecol Resour. 1–16. doi:10.1111/1755-0998.13768. 36785926

[iyad207-B16] Botero CA, Weissing FJ, Wright J, Rubenstein DR. 2015. Evolutionary tipping points in the capacity to adapt to environmental change. Proc Natl Acad Sci. 112(1):184–189. doi:10.1073/pnas.1408589111. 25422451 PMC4291647

[iyad207-B17] Boulétreau-Merle J, Fouillet P, Terrier O. 1987. Seasonal variations and balanced polymorphisms in the reproductive potential of temperate *D. Melanogaster* populations. Entomol Exp Appl. 43(1):39–48. doi:10.1111/j.1570-7458.1987.tb02200.x.

[iyad207-B18] Bourgeois YXC, Warren BH. 2021. An overview of current population genomics methods for the analysis of whole-genome resequencing data in eukaryotes. Mol Ecol. 30(23):6036–6071. doi:10.1111/mec.15989. 34009688

[iyad207-B19] Buffalo V . 2021. Quantifying the relationship between genetic diversity and population size suggests natural selection cannot explain Lewontin's paradox. eLife. 10:e67509. doi:10.7554/eLife.67509. 34409937 PMC8486380

[iyad207-B20] Buffalo V, Coop G. 2019. The linked selection signature of rapid adaptation in temporal genomic data. Genetics. 213(3):1007–1045. doi:10.1534/genetics.119.302581. 31558582 PMC6827383

[iyad207-B21] Buffalo V, Coop G. 2020. Estimating the genome-wide contribution of selection to temporal allele frequency change. Proc Natl Acad Sci U S A. 117(34):20672–20680. doi:10.1073/pnas.1919039117. 32817464 PMC7456072

[iyad207-B22] Bürger R, Gimelfarb A. 2002. Fluctuating environments and the role of mutation in maintaining quantitative genetic variation. Genet Res. 80(1):31–46. doi:10.1017/S0016672302005682. 12448856

[iyad207-B23] Charlesworth D . 2016. The status of supergenes in the 21st century: recombination suppression in B atesian mimicry and sex chromosomes and other complex adaptations. Evol Appl. 9(1):74–90. doi:10.1111/eva.12291. 27087840 PMC4780387

[iyad207-B24] Charlesworth B, Giesel JT. 1972. Selection in populations with overlapping generations. II. Relations between gene frequency and demographic variables. Am Nat. 106(949):388–401. doi:10.1086/282778.

[iyad207-B25] Chen H, Huffman JE, Brody JA, Wang C, Lee S, Li Z, Gogarten SM, Sofer T, Bielak LF, Bis JC, et al 2019. Efficient variant set mixed model association tests for continuous and binary traits in large-scale whole-genome sequencing studies. Am J Hum Genet. 104(2):260–274. doi:10.1016/j.ajhg.2018.12.012. 30639324 PMC6372261

[iyad207-B26] Chevin L, Gompert Z, Nosil P. 2022. Frequency dependence and the predictability of evolution in a changing environment. Evol Lett. 6(1):21–33. doi:10.1002/evl3.266. 35127135 PMC8802243

[iyad207-B27] Cogni R, Kuczynski K, Lavington E, Koury S, Behrman EL, O'Brien KR, Schmidt PS, Eanes WF. 2015. Variation in Drosophila melanogaster central metabolic genes appears driven by natural selection both within and between populations. Proc Biol Sci. 282(1800):20142688. doi:10.1098/rspb.2014.2688. 25520361 PMC4298213

[iyad207-B28] Corbett-Detig RB, Hartl DL. 2012. Population genomics of inversion polymorphisms in *Drosophila melanogaster*. PLoS Genet. 8(12):e1003056. doi:10.1371/journal.pgen.1003056. 23284285 PMC3527211

[iyad207-B29] Csilléry K, François O, Blum MGB. 2012. Abc: an R package for approximate Bayesian computation (ABC). Methods Ecol Evol. 3(3):475–479. doi:10.1111/j.2041-210X.2011.00179.x.

[iyad207-B30] Danecek P, Auton A, Abecasis G, Albers CA, Banks E, DePristo MA., Handsaker RE, Lunter G, Marth GT, Sherry ST, et al 2011. The variant call format and VCFtools. Bioinformatics. 27(15):2156–2158. doi:10.1093/bioinformatics/btr330. 21653522 PMC3137218

[iyad207-B31] de Villemereuil P, Charmantier A, Arlt D, Bize P, Brekke P, Brouwer L, Cockburn A, Côté SD, Dobson FS, Evans SR, et al 2020. Fluctuating optimum and temporally variable selection on breeding date in birds and mammals. Proc Natl Acad Sci. 117(50):31969–31978. doi:10.1073/pnas.2009003117. 33257553 PMC7116484

[iyad207-B32] Dobzhansky T . 1943. Genetics of natural populations IX. Temporal changes in the composition of populations of *Drosophila pseudoobscura*. Genetics. 28(2):162–186. doi:10.1093/genetics/28.2.162. 17247077 PMC1209199

[iyad207-B33] Dobzhansky T . 1947. Genetics of natural populations. XIV. A response of certain gene arrangements in the third chromosome of *Drosophila pseudoobscura* to natural selection. Genetics. 32(2):142–160. doi:10.1093/genetics/32.2.142. 20292738 PMC1209369

[iyad207-B34] Dobzhansky T, Ayala FJ. 1973. Temporal frequency changes of enzyme and chromosomal polymorphisms in natural populations of *Drosophila*. Proc Natl Acad Sci U S A. 70(3):680–683. doi:10.1073/pnas.70.3.680. 4514981 PMC433334

[iyad207-B35] Dobzhansky T, Wright S. 1943. Genetics of natural populations. X. Dispersion rates in *Drosophila Pseudoobscura*. Genetics. 28(4):304–340. doi:10.1093/genetics/28.4.304. 17247091 PMC1209213

[iyad207-B36] Elhanany-Tamir H, Yu YV, Shnayder M, Jain A, Welte M, Volk T. 2012. Organelle positioning in muscles requires cooperation between two KASH proteins and microtubules. J. Cell Biol. 198(5):833–846. doi:10.1083/jcb.201204102. 22927463 PMC3432764

[iyad207-B37] Erickson PA, Weller CA, Song DY, Bangerter AS, Schmidt P, Bergland AO. 2020. Unique genetic signatures of local adaptation over space and time for diapause, an ecologically relevant complex trait, in Drosophila melanogaster. PLoS Genet. 16(11):e1009110. doi:10.1371/journal.pgen.1009110. 33216740 PMC7717581

[iyad207-B38] Feder AF, Petrov DA, Bergland AO. 2012. LDx: estimation of linkage disequilibrium from high-throughput pooled resequencing data. PLoS One. 7(11):e48588. doi:10.1371/journal.pone.0048588. 23152785 PMC3494690

[iyad207-B39] Forester BR, Lasky JR, Wagner HH, Urban DL. 2018. Comparing methods for detecting multilocus adaptation with multivariate genotype–environment associations. Mol Ecol. 27(9):2215–2233. doi:10.1111/mec.14584. 29633402

[iyad207-B40] Gao Z, Przeworski M, Sella G. 2015. Footprints of ancient-balanced polymorphisms in genetic variation data from closely related species. Evolution. 69(2):431–446. doi:10.1111/evo.12567. 25403856 PMC4335603

[iyad207-B41] Garrigan D, Kingan SB, Geneva AJ, Vedanayagam JP, Presgraves DC. 2014. Genome diversity and divergence in *Drosophila mauritiana*: multiple signatures of faster X evolution. Genome Biol. Evol. 6(9):2444–2458. doi:10.1093/gbe/evu198. 25193308 PMC4202334

[iyad207-B42] Garud NR, Messer PW, Buzbas EO, Petrov DA. 2015. Recent selective sweeps in North American *Drosophila melanogaster* show signatures of soft sweeps. PLoS Genet. 11(2):e1005004. doi:10.1371/journal.pgen.1005004. 25706129 PMC4338236

[iyad207-B43] Garud NR, Messer PW, Petrov DA. 2021. Detection of hard and soft selective sweeps from *Drosophila melanogaster* population genomic data. PLOS Genet. 17(2):e1009373. doi:10.1371/journal.pgen.1009373. 33635910 PMC7946363

[iyad207-B44] Garud NR, Petrov DA. 2016. Elevated linkage disequilibrium and signatures of soft sweeps are common in *Drosophila melanogaster*. Genetics. 203(2):863–880. doi:10.1534/genetics.115.184002. 27098909 PMC4896199

[iyad207-B45] Gautier M . 2015. Genome-Wide scan for adaptive divergence and association with population-specific covariates. Genetics. 201(4):1555–1579. doi:10.1534/genetics.115.181453. 26482796 PMC4676524

[iyad207-B46] Gautier M, Vitalis R, Flori L, Estoup A. 2022. *F* -statistics estimation and admixture graph construction with pool-seq or allele count data using the R package *poolfstat*. Mol Ecol Resour. 22(4):1394–1416. doi:10.1111/1755-0998.13557. 34837462

[iyad207-B47] Glaser-Schmitt A, Ramnarine TJS, Parsch J. 2023. Rapid evolutionary change, constraints and the maintenance of polymorphism in natural populations of *Drosophila melanogaster*. Mol Ecol. 1–14. doi:10.1111/mec.17024. 37222070

[iyad207-B48] Glaser-Schmitt A, Wittmann MJ, Ramnarine TJS, Parsch J. 2021. Sexual antagonism, temporally fluctuating selection, and Variable dominance affect a regulatory polymorphism in *Drosophila melanogaster*. Mol Biol Evol. 38(11):4891–4907. doi:10.1093/molbev/msab215. 34289067 PMC8557461

[iyad207-B49] Gleason JM, Roy PR, Everman ER, Gleason TC, Morgan TJ. 2019. Phenology of Drosophila species across a temperate growing season and implications for behavior. PLoS One. 14(5):e0216601. doi:10.1371/journal.pone.0216601. 31095588 PMC6521991

[iyad207-B50] Gramates LS, Agapite J, Attrill H, Calvi BR, Crosby MA, Santos Dos, Goodman G, Goutte-Gattat JL, Jenkins D, Kaufman VK, et al 2022. FlyBase: a guided tour of highlighted features. Genetics. 220(4):iyac035. 10.1093/genetics/iyac035. 35266522 PMC8982030

[iyad207-B51] Grant PR, Grant BR. 2002. Unpredictable evolution in a 30-year study of Darwin's finches. Science. 296(5568):707–711. doi:10.1126/science.1070315. 11976447

[iyad207-B52] Grenier JK, Arguello JR, Moreira MC, Gottipati S, Mohammed J, Hackett SR, Boughton R, Greenberg AJ, Clark AG. 2015. Global diversity lines–A five-continent reference panel of sequenced *Drosophila melanogaster* strains. G3 (Bethesda). 5(4):593–603. doi:10.1534/g3.114.015883. 25673134 PMC4390575

[iyad207-B53] Hager ER, Harringmeyer OS, Wooldridge TB, Theingi S, Gable JT, McFadden S, Neugeboren B, Turner KM, Jensen JD, Hoekstra HE. 2022. A chromosomal inversion contributes to divergence in multiple traits between deer mouse ecotypes. Science. 377(6604):399–405. doi:10.1126/science.abg0718. 35862520 PMC9571565

[iyad207-B54] Haller BC, Messer PW. 2019. SLim 3: forward genetic simulations beyond the wright–fisher model. Mol Biol Evol. 36(3):632–637. doi:10.1093/molbev/msy228. 30517680 PMC6389312

[iyad207-B55] Hedrick PW . 1976. Genetic variation in a heterogeneous environment. II. Temporal heterogeneity and directional selection. Genetics. 84(1):145–157. doi:10.1093/genetics/84.1.145. 992363 PMC1213561

[iyad207-B56] Hedrick PW . 2012. What is the evidence for heterozygote advantage selection? Trends Ecol. Evol. 27(12):698–704. doi:10.1016/j.tree.2012.08.012. 22975220

[iyad207-B57] Huang W, Massouras A, Inoue Y, Peiffer J, Ràmia M, Tarone AM, Turlapati L, Zichner T, Zhu D, Lyman RF, et al 2014. Natural variation in genome architecture among 205 *Drosophila melanogaster* genetic reference panel lines. Genome Res. 24(7):1193–1208. doi:10.1101/gr.171546.113. 24714809 PMC4079974

[iyad207-B58] Ives PT . 1970. Further genetic studies of the south amherst population of *Drosophila melanogaster*. Evolution. 24(3):507–518. doi:10.1111/j.1558-5646.1970.tb01785.x. 28562987

[iyad207-B59] Johnson OL, Tobler R, Schmidt JM, Huber CD. 2023. Fluctuating selection and the determinants of genetic variation. Trends Genet. 39(6):491–504. doi:10.1016/j.tig.2023.02.004. 36890036

[iyad207-B60] Jombart T, Devillard S, Balloux F. 2010. Discriminant analysis of principal components: a new method for the analysis of genetically structured populations. BMC Genet. 11(1):94. doi:10.1186/1471-2156-11-94. 20950446 PMC2973851

[iyad207-B61] Jordan KW, Craver KL, Magwire MM, Cubilla CE, Mackay TFC, Anholt RRH. 2012. Genome-Wide association for sensitivity to chronic oxidative stress in *Drosophila melanogaster*. PLoS One. 7(6):e38722. doi:10.1371/journal.pone.0038722. 22715409 PMC3371005

[iyad207-B62] Kamping, Delden WV. 1999. A long-term study on interactions between the adh and αGpdh allozyme polymorphisms and the chromosomal inversion In(2L)t in a seminatural population of *D. melanogaster*. J Evol Biol. 12(4):809–821. doi:10.1046/j.1420-9101.1999.00083.x.

[iyad207-B63] Kapopoulou A, Kapun M, Pieper B, Pavlidis P, Wilches R, Duchen P, Stephan W, Laurent S. 2020. Demographic analyses of a new sample of haploid genomes from a Swedish population of Drosophila melanogaster. Sci Rep. 10:22415. 10.1038/s41598-020-79720-1. 33376238 PMC7772335

[iyad207-B64] Kapun M, Barrón MG, Staubach F, Obbard DJ, Wiberg RAW, Vieira J, Goubert C, Rota-Stabelli O, Kankare M, Bogaerts-Márquez M, et al 2020. Genomic analysis of European *Drosophila melanogaster* populations reveals longitudinal structure, continent-wide selection, and previously unknown DNA viruses. Mol Biol Evol. 37(9):2661–2678. doi:10.1093/molbev/msaa120. 32413142 PMC7475034

[iyad207-B65] Kapun M, Fabian DK, Goudet J, Flatt T. 2016. Genomic evidence for adaptive inversion clines in *Drosophila melanogaster*. Mol Biol Evol. 33(5):1317–1336. doi:10.1093/molbev/msw016. 26796550

[iyad207-B66] Kapun M, Flatt T. 2019. The adaptive significance of chromosomal inversion polymorphisms in *Drosophila melanogaster*. Mol Ecol. 28(6):1263–1282. doi:10.1111/mec.14871. 30230076

[iyad207-B67] Kapun M, Mitchell ED, Kawecki TJ, Schmidt P, Flatt T. 2023. An ancestral balanced inversion polymorphism confers global adaptation. Mol Biol Evol. 40(6):msad118. doi:10.1093/molbev/msad118. 37220650 PMC10234209

[iyad207-B68] Kapun M, Nunez JCB, Bogaerts-Márquez M, Murga-Moreno J, Paris M, Outten J, Coronado-Zamora M, Tern C, Rota-Stabelli O, Guerreiro MPG, et al 2021. *Drosophila* evolution over space and time (DEST): a new population genomics resource. Mol Biol Evol. 38(12):5782–5805. doi:10.1093/molbev/msab259. 34469576 PMC8662648

[iyad207-B69] Karasov T, Messer PW, Petrov DA. 2010. Evidence that adaptation in *Drosophila* is not limited by mutation at single sites. PLoS Genet. 6(6):e1000924. doi:10.1371/journal.pgen.1000924. 20585551 PMC2887467

[iyad207-B70] Kirkpatrick M, Barton N. 2006. Chromosome inversions, local adaptation and speciation. Genetics. 173(1):419–434. doi:10.1534/genetics.105.047985. 16204214 PMC1461441

[iyad207-B71] Kirubakaran TG, Grove H, Kent MP, Sandve SR, Baranski M, Nome T, De Rosa MC, Righino B, Johansen T, Otterå H, et al 2016. Two adjacent inversions maintain genomic differentiation between migratory and stationary ecotypes of atlantic cod. Mol Ecol. 25(10):2130–2143. doi:10.1111/mec.13592. 26923504

[iyad207-B72] Kolaczkowski B, Kern AD, Holloway AK, Begun DJ. 2011. Genomic differentiation between temperate and tropical Australian populations of Drosophila melanogaster. Genetics. 187(1):245–260. doi:10.1534/genetics.110.123059. 21059887 PMC3018305

[iyad207-B73] Küpper C, Stocks M, Risse JE, dos Remedios N, Farrell LL, McRae SB, Morgan TC, Karlionova N, Pinchuk P, Verkuil YI, et al 2016. A supergene determines highly divergent male reproductive morphs in the ruff. Nat Genet. 48(1):79–83. doi:10.1038/ng.3443. 26569125 PMC5218575

[iyad207-B74] Lack JB, Cardeno CM, Crepeau MW, Taylor W, Corbett-Detig RB, Stevens KA, Langley CH, Pool JE. 2015. The *Drosophila* genome nexus: a population genomic resource of 623 *Drosophila melanogaster* genomes, including 197 from a single ancestral range population. Genetics. 199(4):1229–1241. doi:10.1534/genetics.115.174664. 25631317 PMC4391556

[iyad207-B76] Lange JD, Bastide H, Lack JB, Pool JE. 2022. A population genomic assessment of three decades of evolution in a natural *Drosophila* population. Mol Biol Evol. 39(2):msab368. doi:10.1093/molbev/msab368. 34971382 PMC8826484

[iyad207-B77] Lavington E, Kern AD. 2017. The effect of common inversion polymorphisms *In(2L)t* and *In(3R)Mo* on patterns of transcriptional variation in *Drosophila melanogaster*. G3 (Bethesda). 7(11):3659–3668. doi:10.1534/g3.117.1133. 28916647 PMC5677173

[iyad207-B78] Lê S, Josse J, Husson F. 2008. FactoMineR: an *R* package for multivariate analysis. J Stat Softw. 25(1):1–18. doi:10.18637/jss.v025.i01.

[iyad207-B79] Lemeunier F, Aulard S. 1992. Inversion polymorphism in Drosophila melanogaster. In: Krimbas CB, Powell JR, editors. Drosophila Inversion Polymorphism. Boca Raton (FL): CRC Press. p. 339–405.

[iyad207-B80] Li H, Handsaker B, Wysoker A, Fennell T, Ruan J, Homer N, Marth G. 2009. The sequence alignment/map format and SAMtools. Bioinformatics. 25(16):2078–2079. doi:10.1093/bioinformatics/btp352. 19505943 PMC2723002

[iyad207-B81] Lotterhos KE . 2023. The paradox of adaptive trait clines with nonclinal patterns in the underlying genes. Proc Natl Acad Sci U S A. 120(12):e2220313120. doi:10.1073/pnas.2220313120. PMC10041142 36917658

[iyad207-B82] Lotterhos KE, Card DC, Schaal SM, Wang L, Collins C, et al 2017. Composite measures of selection can improve the signal-to-noise ratio in genome scans. Methods Ecol Evol. 8(6):717–727. doi:10.1111/2041-210X.12774.

[iyad207-B83] Lotterhos KE, Whitlock MC. 2014. Evaluation of demographic history and neutral parameterization on the performance of *F*_ST_ outlier tests. Mol Ecol. 23(9):2178–2192. doi:10.1111/mec.12725. 24655127 PMC4228763

[iyad207-B84] Luo L, Tang Z, Schoville SD, Zhu J. 2021. A comprehensive analysis comparing linear and generalized linear models in detecting adaptive SNPs. Mol Ecol Resour. 21(3):733–744. doi:10.1111/1755-0998.13298. 33217107

[iyad207-B85] Lynch M, Ho W-C. 2020. The limits to estimating population-genetic parameters with temporal data. Genome Biol Evol. 12(4):443–455. doi:10.1093/gbe/evaa056. 32181820 PMC7197491

[iyad207-B86] Machado HE, Bergland AO, Taylor R, Tilk S, Behrman E, Dyer K, Fabian DK, Flatt T, González J, Karasov TL, et al 2021. Broad geographic sampling reveals the shared basis and environmental correlates of seasonal adaptation in *Drosophila*. eLife. 10:e67577. doi:10.7554/eLife.67577. 34155971 PMC8248982

[iyad207-B87] Mackay TFC, Richards S, Stone EA, Barbadilla A, Ayroles JF, Zhu D, Casillas S, Han Y, Magwire MM, Cridland JM, et al 2012. The *Drosophila melanogaster* genetic reference panel. Nature. 482(7384):173–178. doi:10.1038/nature10811. 22318601 PMC3683990

[iyad207-B88] Marschner IC . 2011. Glm2: fitting generalized linear models with convergence problems. R J. 3(2):12. doi:10.32614/RJ-2011-012.

[iyad207-B89] Meyer D, Dimitriadou E, Hornik K, Weingessel A, Leisch F, C-C Chang, C-C Lin. 2023. Misc Functions of the Department of Statistics, Probability Theory Group (Formerly: E1071). https://cran.rproject.org/web/packages/e1071/index.html.

[iyad207-B90] Morrissey MB, Hadfield JD. 2012. Directional selection in temporally replicated studies is remarkably consistent: consistency of selection. Evolution. 66(2):435–442. doi:10.1111/j.1558-5646.2011.01444.x. 22276539

[iyad207-B91] Nielsen R . 2001. Statistical tests of selective neutrality in the age of genomics. Heredity (Edinb). 86(6):641–647. doi:10.1046/j.1365-2540.2001.00895.x. 11595044

[iyad207-B92] Nosil P, Villoutreix R, De Carvalho CF, Farkas TE, Soria-Carrasco V, Feder JL, Crespi BJ, Gompert Z. 2018. Natural selection and the predictability of evolution in *Timema* stick insects. Science. 359(6377):765–770. doi:10.1126/science.aap9125. 29449486

[iyad207-B93] Ohta T . 1971. Associative overdominance caused by linked detrimental mutations. Genet Res. 18(3):277–286. doi:10.1017/S0016672300012684. 5158298

[iyad207-B94] Olazcuaga L, Foucaud J, Deschamps C, Loiseau A, Claret J-L, Vedovato R, Guilhot R, Sévely C, Gautier M, Hufbauer RA, et al 2022. Rapid and transient evolution of local adaptation to seasonal host fruits in an invasive pest fly. Evol Lett. 6(6):490–505. doi:10.1002/evl3.304. 36579160 PMC9783429

[iyad207-B95] Paaby AB, Bergland AO, Behrman EL, Schmidt PS. 2014. A highly pleiotropic amino acid polymorphism in the *Drosophila* insulin receptor contributes to life-history adaptation. Evolution. 68(12):3395–3409. doi:10.1111/evo.12546. 25319083 PMC5079517

[iyad207-B96] Pool JE . 2015. The mosaic ancestry of the *Drosophila* genetic reference panel and the *D. melanogaster* reference genome reveals a network of epistatic fitness interactions. Mol Biol Evol. 32(12):3236–3251. doi:10.1093/molbev/msv194. 26354524 PMC4652625

[iyad207-B97] Pool JE, Corbett-Detig RB, Sugino RP, Stevens KA, Cardeno CM, Crepeau MW, Duchen P, Emerson JJ, Saelao P, Begun DJ, et al 2012. Population genomics of sub-saharan *Drosophila melanogaster*: african diversity and non-African admixture. PLoS Genet. 8(12):e1003080. doi:10.1371/journal.pgen.1003080. 23284287 PMC3527209

[iyad207-B98] Purcell S, Neale B, Todd-Brown K, Thomas L, Ferreira MAR, Bender D, Maller J, Sklar P, de Bakker PIW, Daly MJ, et al 2007. PLINK: a tool set for whole-genome association and population-based linkage analyses. Am J Hum Genet. 81(3):559–575. doi:10.1086/519795. 17701901 PMC1950838

[iyad207-B99] Rajpurohit S, Gefen E, Bergland AO, Petrov DA, Gibbs AG, Schmidt PS. 2018. Spatiotemporal dynamics and genome-wide association analysis of desiccation tolerance in *Drosophila melanogaster*. Mol Ecol. 27(17):3525–3540. doi:10.1111/mec.14814. 30051644 PMC6129450

[iyad207-B100] Reimchen TE, Nosil P. 2002. Temporal variation in divergent selection on spine number in threespine stickleback. Evolution. 56(12):2472–2483. doi:10.1111/j.0014-3820.2002.tb00172.x. 12583587

[iyad207-B101] Rezende E, Balanyà J, Rodríguez-Trelles F, Rego C, Fragata I, Matos M, Serra L, Santos M. 2010. Climate change and chromosomal inversions in Drosophila subobscura. Clim. Res. 43(1):103–114. doi:10.3354/cr00869.

[iyad207-B102] Rodríguez-Trelles F, Alvarez G, Zapata C. 1996. Time-Series analysis of seasonal changes of the *O* inversion polymorphism of *Drosophila subobscura*. Genetics. 142(1):179–187. doi:10.1093/genetics/142.1.179. 8770595 PMC1206946

[iyad207-B103] Rodrigues MF, Vibranovski MD, Cogni R. 2021. Clinal and seasonal changes are correlated in *Drosophila melanogaster*. Evolution. 75(8):2042–2054. doi:10.1111/evo.14300. 34184262

[iyad207-B104] Rosenberg-Hasson Y, Renert-Pasca M, Volk T. 1996. A *Drosophila* dystrophin-related protein, MSP-300, is required for embryonic muscle morphogenesis. Mech Dev. 60(1):83–94. doi:10.1016/S0925-4773(96)00602-8. 9025063

[iyad207-B105] Rudman SM, Greenblum SI, Rajpurohit S, Betancourt NJ, Hanna J, Tilk S, Yokoyama T, Petrov DA, Schmidt P. 2022. Direct observation of adaptive tracking on ecological time scales in *Drosophila*. Science. 375(6586):eabj7484. doi:10.1126/science.abj7484. 35298245 PMC10684103

[iyad207-B106] Said I, Byrne A, Serrano V, Cardeno C, Vollmers C, Corbett-Detig R. 2018. Linked genetic variation and not genome structure causes widespread differential expression associated with chromosomal inversions. Proc Natl Acad Sci U S A. 115(21):5492–5497. doi:10.1073/pnas.1721275115. 29735663 PMC6003460

[iyad207-B107] Sanchez-Refusta F, Santiago E, Rubio J. 1990. Seasonal fluctuations of cosmopolitan inversion frequencies in a natural population of *Drosophila melanogaster*. Genet Sel Evol. 22(1):47–56. doi:10.1186/1297-9686-22-1-47.

[iyad207-B108] Schaal SM, Haller BC, Lotterhos KE. 2022. Inversion invasions: when the genetic basis of local adaptation is concentrated within inversions in the face of gene flow. Philos Trans R Soc B Biol Sci. 377(1856):20210200. doi:10.1098/rstb.2021.0200. PMC9189506 35694752

[iyad207-B109] Schlötterer C, Tobler R, Kofler R, Nolte V. 2014. Sequencing pools of individuals—mining genome-wide polymorphism data without big funding. Nat Rev Genet. 15(11):749–763. doi:10.1038/nrg3803. 25246196

[iyad207-B110] Schmidt PS, Conde DR. 2006. Environmental heterogeneity and the maintenance of genetic variation for reproductive diapause in *Drosophila melanogaster*. Evolution. 60(8):1602–1611. 10.1111/j.0014-3820.2006.tb00505.x. 17017061

[iyad207-B111] Schmidt PS, Zhu C-T, Das J, Batavia M, Yang L, Eanes WF. 2008. An amino acid polymorphism in the *couch potato* gene forms the basis for climatic adaptation in *Drosophila melanogaster*. Proc Natl Acad Sci U S A. 105(42):16207–16211. doi:10.1073/pnas.0805485105. 18852464 PMC2570987

[iyad207-B112] Schrider DR, Ayroles J, Matute DR, Kern AD. 2018. Supervised machine learning reveals introgressed loci in the genomes of *Drosophila simulans* and *D. sechellia*. PLOS Genet. 14(4):e1007341. doi:10.1371/journal.pgen.1007341. 29684059 PMC5933812

[iyad207-B113] Schwander T, Libbrecht R, Keller L. 2014. Supergenes and Complex phenotypes. Curr Biol. 24(7):R288–R294. doi:10.1016/j.cub.2014.01.056. 24698381

[iyad207-B114] Signor SA, New FN, Nuzhdin S. 2018. A large panel of *Drosophila simulans* reveals an abundance of common variants. Genome Biol Evol. 10(1):189–206. doi:10.1093/gbe/evx262. 29228179 PMC5767965

[iyad207-B115] Simonsen KL, Churchill GA, Aquadro CF. 1995. Properties of statistical tests of neutrality for DNA polymorphism data. Genetics. 141(1):413–429. doi:10.1093/genetics/141.1.413. 8536987 PMC1206737

[iyad207-B116] Slater G, Birney E. 2005. Automated generation of heuristics for biological sequence comparison. BMC Bioinformatics. 6(1):31. doi:10.1186/1471-2105-6-31. 15713233 PMC553969

[iyad207-B117] Sparks A . 2018. Nasapower: a NASA POWER global meteorology, surface solar energy and climatology data client for R. J. Open Source Softw. 3(30):1035. doi:10.21105/joss.01035.

[iyad207-B118] Stalker HD . 1980. Chromosome studies in wild populations of *Drosophila melanogaster*. II. Relationship of inversion frequencies to latitude, season, wing-loading and flight activity. Genetics. 95(1):211–223. doi:10.1093/genetics/95.1.211. 17249033 PMC1214217

[iyad207-B119] Stephan W, Li H. 2007. The recent demographic and adaptive history of *Drosophila melanogaster*. Heredity (Edinb). 98(2):65–68. doi:10.1038/sj.hdy.6800901. 17006533

[iyad207-B120] Storey J, Bass A, Dabney A, Robinson D. 2010. qvalue: Q-value estimation for false discovery rate control. https://www.bioconductor.org/packages/devel/bioc/manuals/qvalue/man/qvalue.pdf.

[iyad207-B121] Stouffer SA, Suchman EA, DeVinney LC, Star SA, Williams RM Jr. 1949. The American Soldier: Adjustment During Army Life. (Studies in Social Psychology in World War II), vol. 1. Princeton (NJ): Princeton University Press.

[iyad207-B122] Taus T, Futschik A, Schlötterer C. 2017. Quantifying selection with pool-seq time series data. Mol Biol Evol. 34(11):3023–3034. doi:10.1093/molbev/msx225. 28961717 PMC5850601

[iyad207-B123] Thompson MJ, Jiggins CD. 2014. Supergenes and their role in evolution. Heredity (Edinb). 113(1):1–8. doi:10.1038/hdy.2014.20. 24642887 PMC4815649

[iyad207-B124] Turissini DA, Matute DR. 2017. Fine scale mapping of genomic introgressions within the Drosophila yakuba clade. PLOS Genet. 13(9):e1006971. doi:10.1371/journal.pgen.1006971. 28873409 PMC5600410

[iyad207-B125] Unckless RL, Howick VM, Lazzaro BP. 2016. Convergent balancing selection on an antimicrobial peptide in *Drosophila*. Curr Biol. 26(2):257–262. doi:10.1016/j.cub.2015.11.063. 26776733 PMC4729654

[iyad207-B126] van Delden W, Kamping A. 1989. The association between the polymorphisms at the adh and αGpdh loci and the In(2L)t inversion in *Drosophila melanogaster* in relation to temperature. Evolution. 43(4):775–793. doi:10.1111/j.1558-5646.1989.tb05176.x. 28564191

[iyad207-B127] van Delden W, Kamping A. 1991. Changes in relative fitness with temperature among second chromosome arrangements in *Drosophila melanogaster*. Genetics. 127(3):507–514. doi:10.1093/genetics/127.3.507. 1901819 PMC1204378

[iyad207-B128] van Delden W, Kamping A. 1997. Worldwide latitudinal clines for the alcohol dehydrogenase polymorphism in Drosophila melanogaster: what is the unit of selection? In: Bijlsma R, Loeschcke V, editors. Environmental Stress, Adaptation and Evolution, Experientia Supplementum. Basel: Birkhäuser Basel. p. 97–115. 10.1007/978-3-0348-8882-0_6 9342845

[iyad207-B129] Van der Auwera GA, O’Connor BD. 2020. Genomics in the Cloud: Using Docker, GATK, and WDL in Terra (1st Edition). O'Reilly Media.

[iyad207-B141] van't Land J . 1997. Latitudinal Variation in Drosophila Melanogaster. Groningen, The Netherlands: University of Groningen.

[iyad207-B130] Wellenreuther M, Bernatchez L. 2018. Eco-Evolutionary genomics of chromosomal inversions. Trends Ecol Evol. 33(6):427–440. doi:10.1016/j.tree.2018.04.002. 29731154

[iyad207-B131] Whitlock MC, Lotterhos KE. 2015. Reliable detection of loci responsible for local adaptation: inference of a null model through trimming the distribution of *F*_ST_. Am Nat. 186(S1):S24–S26. 10.1086/682949. 26656214

[iyad207-B132] Wittmann MJ, Bergland AO, Feldman MW, Schmidt PS, Petrov DA. 2017. Seasonally fluctuating selection can maintain polymorphism at many loci via segregation lift. Proc Natl Acad Sci U S A. 114(46):E9932–E9941. doi:10.1073/pnas.1702994114. 29087300 PMC5699028

[iyad207-B133] Wittmann MJ, Mousset S, Hermisson J. 2023. Modeling the genetic footprint of fluctuating balancing selection: from the local to the genomic scale. Genetics. 223(4):iyad022. doi:10.1093/genetics/iyad022. 36790814

[iyad207-B134] Wright S . 1931. Evolution in Mendelian populations. Genetics. 16(2):97–159. doi:10.1093/genetics/16.2.97. 17246615 PMC1201091

[iyad207-B135] Xie X, Fischer JA. 2008. On the roles of the *Drosophila* KASH domain proteins msp-300 and klarsicht. Fly (Austin). 2(2):74–81. doi:10.4161/fly.6108. 18820482

[iyad207-B136] Xue A, Wang H, Zhu J. 2017. Dissecting genetic architecture of startle response in *Drosophila melanogaster* using multi-omics information. Sci Rep. 7(1):12367. doi:10.1038/s41598-017-11676-1. 28959013 PMC5620086

[iyad207-B137] Yang J, Lee SH, Goddard ME, Visscher PM. 2011. GCTA: a tool for genome-wide Complex trait analysis. Am J Hum Genet. 88(1):76–82. doi:10.1016/j.ajhg.2010.11.011. 21167468 PMC3014363

[iyad207-B138] Yu Y, Bergland AO. 2022. Distinct signals of clinal and seasonal allele frequency change at eQTLs in *Drosophila melanogaster*. Evolution. 76(11):2758–2768. doi:10.1111/evo.14617. 36097359 PMC9710195

[iyad207-B139] Yu J, Starr DA, Wu X, Parkhurst SM, Zhuang Y, Xu T, Xu R, Han M. 2006. The KASH domain protein MSP-300 plays an essential role in nuclear anchoring during *Drosophila* oogenesis. Dev Biol. 289(2):336–345. doi:10.1016/j.ydbio.2005.10.027. 16337624

[iyad207-B140] Zheng X, Levine D, Shen J, Gogarten SM, Laurie C, Weir BS. 2012. A high-performance computing toolset for relatedness and principal component analysis of SNP data. Bioinformatics. 28(24):3326–3328. doi:10.1093/bioinformatics/bts606. 23060615 PMC3519454

