# Supplementary material for: A cosmopolitan inversion facilitates seasonal adaptation in overwintering Drosophila
Source: Genetics. 2023 Dec 5;226(2):iyad207. doi: 10.1093/genetics/iyad207 (PMC10847723; doi:10.1093/genetics/iyad207)
Supplement: iyad207_Supplementary_Data [file iyad207_supplementary_data.zip › Supplementary_Material_GENETICS-2023-306673.pdf]

Supporting Information for

A cosmopolitan inversion facilitates seasonal adaptation in overwintering *Drosophila*

Joaquin C. B. Nunez[1,2]*, Benedict A. Lenhart[1], Alyssa Bangerter[1], Connor S. Murray[1], Giovanni R. Mazzeo[1], Yang Yu[1], Taylor L. Nystrom[1], Courtney Tern[1], Priscilla A. Erickson[1,3], Alan O. Bergland[1]*

Affiliations:
[1] Dept. of Biology, University of Virginia, 90 Geldard Drive, Charlottesville, VA 22901
[2] Dept. of Biology, University of Vermont, 109 Carrigan Drive, Burlington, VT 05405
[3] Dept. of Biology, University of Richmond, 138 UR Drive, Richmond, VA 23173

*Corresponding author(s): aob2x@virginia.edu, Joaquin.Nunez@uvm.edu

---

Supporting Materials include:

Supplemental Material

- Supplementary methods 1: *Bioinformatic details*
- Supplementary methods 2: *Details on startle response assay and analyses*
- Legends for Supplementary Tables
- Location of Supplementary Datasets

Supplemental Tables

- Table S1: Samples used in this analysis.
- Table S2: Correlation analysis between PC 1 projections for each city and the year of collection of the pool.
- Table S3: *P*-values for the population-level Kruskal–Wallis (Overwintering test).
- Table S4: Full results for the resampling analysis of correlations between PC projections and several variables across populations.
- Table S5: Metadata for additional single individuals
- Table S6: Tukey HSD analysis on the haplotype analysis of In(2L)t.
- Table S7: Output of the SVM prediction model. *Columns*: sample ID, quantitative prediction from the SVM, qualitative prediction from the SVM, population.
- Table S8: Phenotypes used in our meta-analysis including their references and DOIs.
- Table S9: Line averages for phenotypes in the DGRP.
- Table S10: Deficiency lines and DGRP crossing scheme.
- Table S11: Statistical output of complementation tests comparing different phenotypes

across the 5 different deficiencies backgrounds.

Supplemental Data

- Data S1: Data object including the SNP-wise GLM output.
- Data S2: Data object including the AIC models enrichment analysis.
- Data S3: Anchor loci.

Supplemental Figures

- Figure S1: Principal component analysis (PCs 1 and 2 shown), at the chromosome arm level, for each population.
- Figure S2: PCA and calculated correlations of PC 1, 2, and 3 with year of collection (r^2_{year}), frequency of inv(2L)t ($r^2_{inv(2L)t)}$, and effective coverage (r^2_{Neff}) using random SNP samples.
- Figure S3: Median correlation between a number of variables of interest (Year, Effective Coverage, Frequency of cosmopolitan inversions) relative to the PC projections (PCs 1, 2, and 3).
- Figure S4: Genetic summary statistics of boom-and-bust simulations.
- Figure S5: Using the DGRP to characterize mutations associated with the In(2L)t inversion.
- Figure S6: Data summarization scheme using the NASA power dataset for environmental variables.
- Figure S7: *BayPass* analyses
- Figure S8: Additional measurements of LD within In(2L)t
- Figure S9: Various metrics of genetic variation in our samples.
- Figure S10: Levels of haplotype diversity within inverted and standard classes in chromosome 2L. F_{ST} within In(2L)t in Africa
- Figure S11: Model search in EU-E, EU-W, and NoA-E.
- Figure S12: *P*-values of the rank normalization test for the best models in EUE and EUW
- Figure S13: Phenotype and GLM analyses
- Figure S14: Explanation of DGRP line choice and the complete observations of three major phenotypes from each of the different genetic backgrounds used in the deficiency line startle response study.
- Figure S15: Estimated frequencies of the inversion In(2L)t across a previous study and this one looking at changes in response to seasonality.

Supplementary Methods

SM1. ***Bioinformatic details***

Details for Pooled sequences**:** Quality control, mapping, SNP calling, and dataset merging were done using the DEST dataset mapping pipeline (https://github.com/DEST-bio/DEST_freeze1) using the optimized settings for the PoolSNP caller (Kapun *et al.* 2020) and enforcing a global average minimum allele frequency of 1%. The DEST mapping pipeline accounts for potential contamination with *D. simulans* in the pools using competitive mapping. We combined the Charlottesville pool-seq with the pool-seq samples from DEST to generate a new dataset that contains 283 pooled samples from 22 countries across 12 years 2003-2018. SNPs inside Repetitive elements, defined by the Interrupted Repeats, Microsatellite, RepeatMasker, SimpleRepeats, and WM_SDust tracks from UCSC Genome Browser (Morgulis *et al.* 2006) were removed from further analysis.

Details for Individual sequences**:** Prior to mapping all individual PE reads were merged into longer reads using *bbmerge* (Grigoriev *et al.* 2012). Merged reads were trimmed using *bbduk* v38.98 (Grigoriev *et al.* 2012), flags: *ftl*=15 *ftr*=285 *qtrim*=w *trimq*=20. Reads were mapped to the *Drosophila* genome (Release 6 plus ISO1 MT; https://www.ncbi.nlm.nih.gov/assembly/GCF_000001215.4/) using BWA-MEM v0.7.17 (Li 2013). Bam file sorting and read deduplication were done using Picard tools v2.27.4 (https://broadinstitute.github.io/picard/). Bam file quality was assessed with qualimap v2.2.1 (Okonechnikov *et al.* 2015). In the case of samples that were sequenced across multiple lanes, bam files were joined into a single bam library using samtools v1.9 merge (Li *et al.* 2009) prior to PCR duplicate removal. GVCFs were created using the HaplotypeCaller program of the GATK v4.2 pipeline (Van der Auwera and O'Connor 2020). SNP calling was done by first generating a GenomicsDBI object using GATK's GenomicsDBImport. SNP calling was done using the GenotypeGVCF program. Single nucleotide polymorphisms (SNPs) were calibrated using VariantRecalibrator using the DGRP as the training set. We used WhatsHap v1.7 (Patterson *et al.* 2015) to conduct read-based phasing, followed by population-based phasing using shapeit v4.2.2 (Delaneau *et al.* 2019). Repetitive elements were filtered using the same framework as the pooled samples.

SM2. ***Details on startle response assay***

To test startle response, we mounted Trikinetic monitors (DAM2 *Drosophila* Activity Monitor) to a vibrating pad (Best Choice Products #SKY3197) within a Percival incubator held at 20°C in constant light. On five consecutive days we assayed a new set of flies from each cross to avoid a day effect, and on each day randomly selected the location of each fly amongst the Trikinetic monitors and wells to avoid a monitor effect. We placed the flies in the monitors overnight with

DAM (v3.10.7) software set to record at 1-second intervals. At 10 AM the following morning, we set the vibrating pad to the lowest setting for 5 seconds, before letting the monitors continue data collection for at least 10 minutes. We repeated the whole experiment in three independent blocks. Prior to data analysis, we averaged the activity data for each fly as activity counts over a window size of 90 seconds, progressing with an interval of 30 seconds.

We estimated the startle response in two ways. First, we calculated startle duration as the time between a fly's peak activity post-stimulus and the return to that fly's basal activity level as defined as the average activity prior to startle. We tested for a failure to complement using a mixed effect model implemented in *lme4* version v1.1-30 (Bates *et al.* 2015), by performing a likelihood-ratio test between the full model to the additive one

$$\text{Full: Startle Duration} = \beta_0 + \beta_1(\text{genotype}) \times \beta_2(\text{background}) + \beta_3(\text{block}) \text{ (eq. 10)},$$

$$\text{Additive: Startle Duration} = \beta_0 + \beta_1(\text{genotype}) + \beta_2(\text{background}) + \beta_3(\text{block}) \text{ (eq. 11)},$$

where genotype is a fixed effect that includes both the inversion status and the ancestral/derived state of any haplotype in that window, the background is a fixed effect factor that considered if that fly has the balancer or deficiency background, and block is a random effect factor that considers which of the three experimental blocks the data was collected from.

The second way that we assessed startle response was by estimating the rate of change of startle-induced activity following stimulation. The startle-response decay rate is the slope of activity per unit of time following stimulation. We scaled activity by dividing the activity rate scores by the basal activity rate, defined as the average activity per minute over the hour prior to stimulus, for each individual fly before fitting models to these scaled activity scores over the span of time starting at peak activity post-stimulus. The rate of change of activity following a startle is the "startle response decay" and we estimated these decay rates using the Emmeans v1.8.1-1 package (Searle *et al.* 1980). To test for failure to complement using a mixed-effect model, we conducted a likelihood-ratio test of the full model to the additive one

$$\text{Full: Activity} = \beta_0 + \beta_1(\text{minute}) \times \beta_2(\text{genotype}) \times \beta_3(\text{background}) + \beta_4(\text{block}) + \beta_5(\text{fly}) \text{ (eq. 12)},$$

$$\text{Additive: Activity} = \beta_0 + \beta_1(\text{minute}) + \beta_2(\text{genotype}) + \beta_3(\text{background}) + \beta_4(\text{block}) + \beta_5(\text{fly}) \text{ (eq. 13)},$$

where β_1(minute) is a fixed effect that tracks the elapsed time in minutes since the peak of post-stimulus activity, β_2(genotype) is a fixed effect that includes both the inversion status and the ancestral/derived state of any haplotype in that window, β_3(background) is a fixed effect factor that considered if that fly has the balancer or deficiency background, β_4(block) is a random effect factor that considers which of the three experimental blocks the data was collected from, and β_5(fly) is the random effect of each individual fly.

Legends for Supplementary Tables

Table S1: Samples used in this analysis. The table has the following headings: sampleId: the name of the pooled or individual sample; country: country of provenance; city: city of provenance; locality: code indicating the country, state, and city where the sample was collected; collectionDate: collection date; nFlies: number of flies pooled, for individual samples this number is 1; SRA_accession; SRA_experiment; type: pooled or individual; PCA.set: TRUE/FALSE whether the sample was used in the PCA analysis; FST.set: TRUE/FALSE whether the sample was used in the F_{ST} analysis; GLM.set: TRUE/FALSE whether the sample was used in the GLM analysis; IND.set: TRUE/FALSE whether the sample was used in the individual analyses. in(2L)t_score: For pooled samples, this value is the frequency of the inversion In(2L)t in the pool. For individual samples, this is the SVM score for a sample having inverted (1), standard (0), or heterozygous (0.5). propSimNorm: The level, standardized, of *D. simulans* contamination in pooled data. Note that metadata for DEST samples can be found at: https://github.com/DEST-bio/DEST_freeze1/tree/main/populationInfo.

Table S2: Correlation analysis between PC 1 projections for each city and the year of collection of the pool. Headers: City: city of provenance; Country/State: country or state from which samples originated; PC: Principal component used to run the correlation; *r*: The correlation between the collection year and the PC projection; r^2: The coefficient of determination of the model; *P-value*: *P-value* of the model; Sig: whether the *P-value* is below 5%.

Table S3: Table showing the *P-values* for the population-level Kruskal–Wallis conducted as part of the Overwintering test.

Table S4: Full results for the resampling analysis of correlations between PC projections and several variables across populations. Columns shown are Population, chromosome, number of SNPs sampled, Principal Component, Median correlation, IQR25, IQR75, IQR05, IQR95, Mean, SD.

Table S5: Metadata for additional single individuals from North Carolina, Pennsylvania, Maine, France, and the Netherlands. Columns are Sample name, location, longitude, latitude, and Citation where the data can be found.

Table S6: Tukey HSD analysis on the haplotype analysis of In(2L)t. Columns shown are comparison (com), the difference in means (diff), lower 95% confidence interval (lwr), upper 95% confidence interval (upr), *P-value* adjusted (p.adj).

Table S7: Output of the SVM prediction model. Columns: sample Id, quantitative prediction from the SVM, qualitative prediction from the SVM, population.

Table S8: Phenotypes used in our meta analysis including their references and DOIs. Columns: id, phenotype name, doi of study, specific phenotype category, general phenotype category.

Table S9: Line averages for phenotypes in the DGRP.

Table S10: Deficiency lines and DGRP crossing scheme.

Table S11: Statistical output of complementation tests comparing different phenotypes across the 5 different deficiencies backgrounds.

Supplementary Datasets

Data S1: Data object including the SNP-wise GLM output.
File: *DataS1.Rdata*
https://zenodo.org/doi/10.5281/zenodo.7305042

Data S2: Data object including the AIC models enrichment analysis.
Files: *DataS2.Model.Selection.v2.Rdata* and *DataS2.top10models.Rdata*
https://zenodo.org/doi/10.5281/zenodo.7305042

Data S3: Anchor SNPs.
File: *haplotag_snps_AFS_pol.Rdata*
https://zenodo.org/doi/10.5281/zenodo.7305042

Supplementary Figures

Figure S1: Principal component analysis (PCs 1 and 2 shown), at the chromosome arm level, for each population. The color indicates the collection year. Shapes indicate the sample type, spring and fall collections (collected at the beginning and end of the growing season), frost (collected prior to a frost event), or time series (indicated bi-weekly collections). Samples are: Akaa (Finland), Broggingen (Germany), Charlottesville (VA), Cross Plains (WI), Linvilla (PA), Munich (Germany), Odessa (Ukraine), Yesiloz (Türkiye).

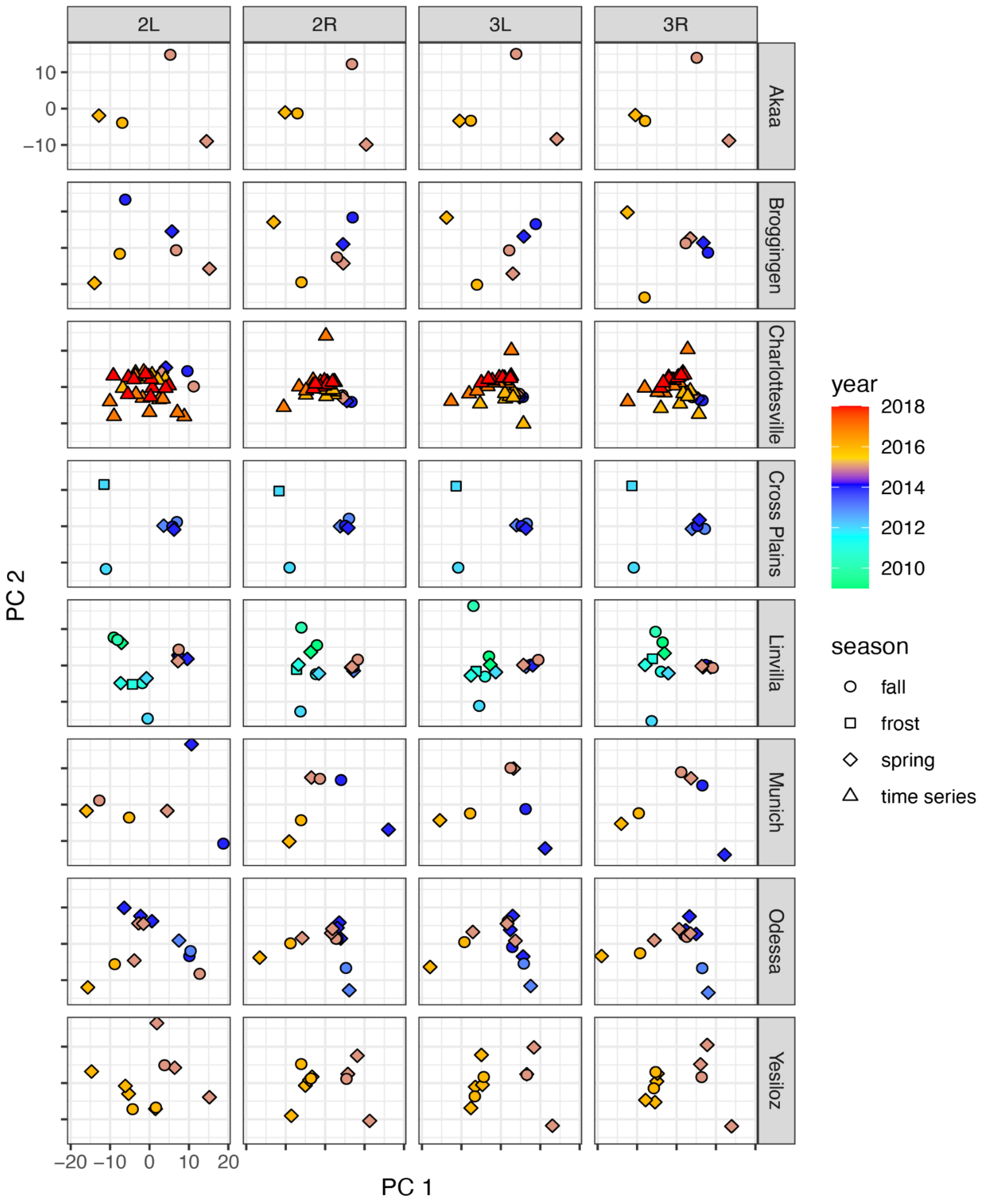

Figure S2: We randomly sampled SNPs in increments of 100, from 100 to 1000 SNPs, and in increments of 1000, from 1000 to 20000 SNPs and performed PCA, and calculated correlations (reported as r^2) of PC 1, 2, and 3 with year of collection (r^2_{year}), frequency of In(2L)t ($r^2_{In(2L)t}$). In parallel, we ran an identical analysis but with sample labels permuted. We repeated this process 500 times each and compared estimates of the real order of the data relative to permutations. Resampling analysis shows correlations between a number of variables of interest (e.g., Year, Frequency In(2L)t) to the PC projections (PCs 1, 2, and 3) in Charlottesville. Red, green, and blue colors represent PCs 1, 2, and 3 respectively. Permutations are shown in purple.

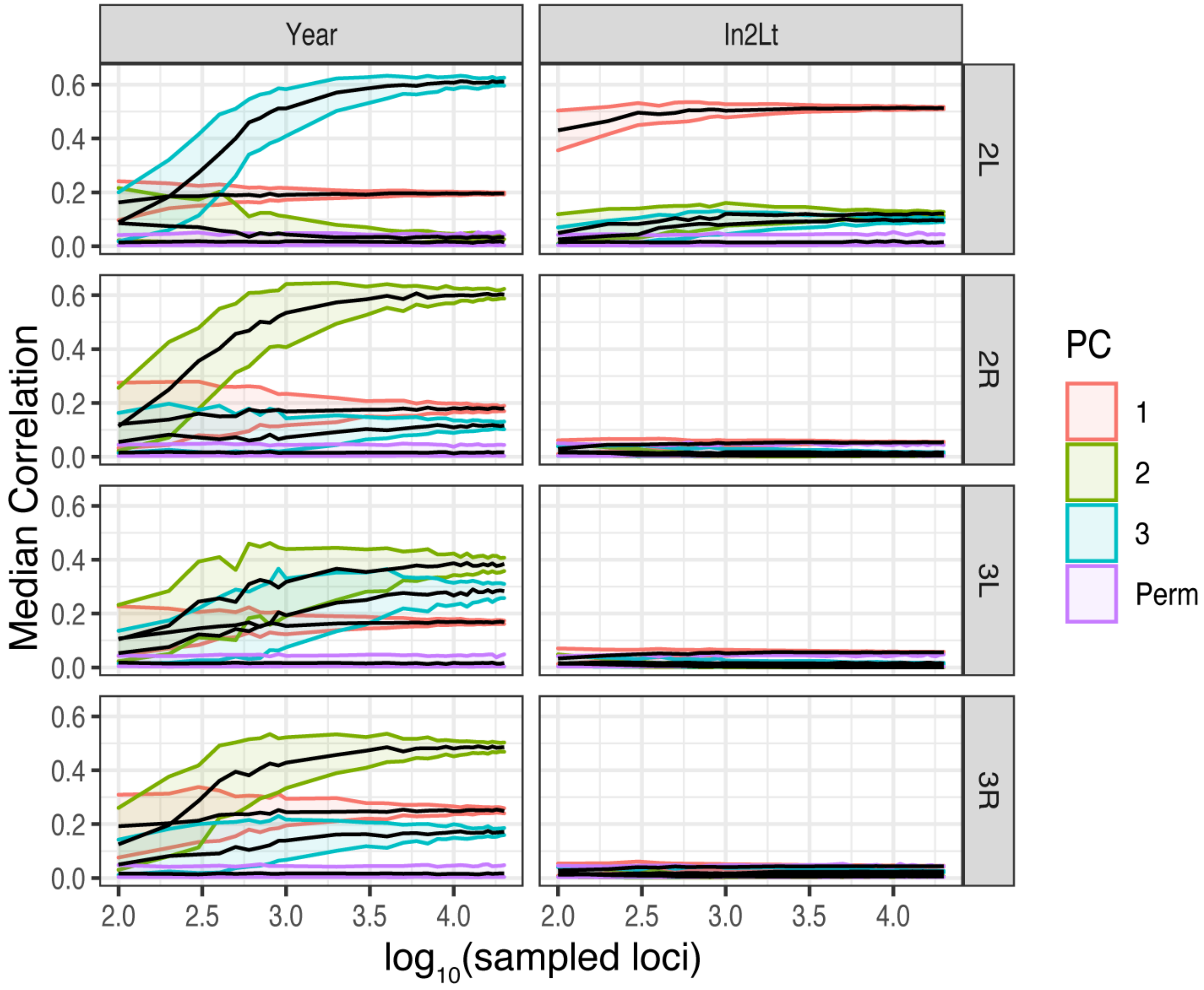

Figure S3: Median correlation between a number of variables of interest (year, effective Coverage - NC, frequency of cosmopolitan inversions) relative to the PC projections (PCs 1, 2, and 3). The confidence intervals represent the 5th and 95th interquartile ranges (IQR). We also show the results of correlations where sample identity has been permuted. Samples are: Aka=Akaa (Finland), Bro=Broggingen (Germany), Cha=Charlottesville (VA), Cp=Cross Plains (WI), Li=Linvilla (PA), Mun=Munich (Germany), Ode=Odessa (Ukraine), Yes=Yesiloz (Türkiye).

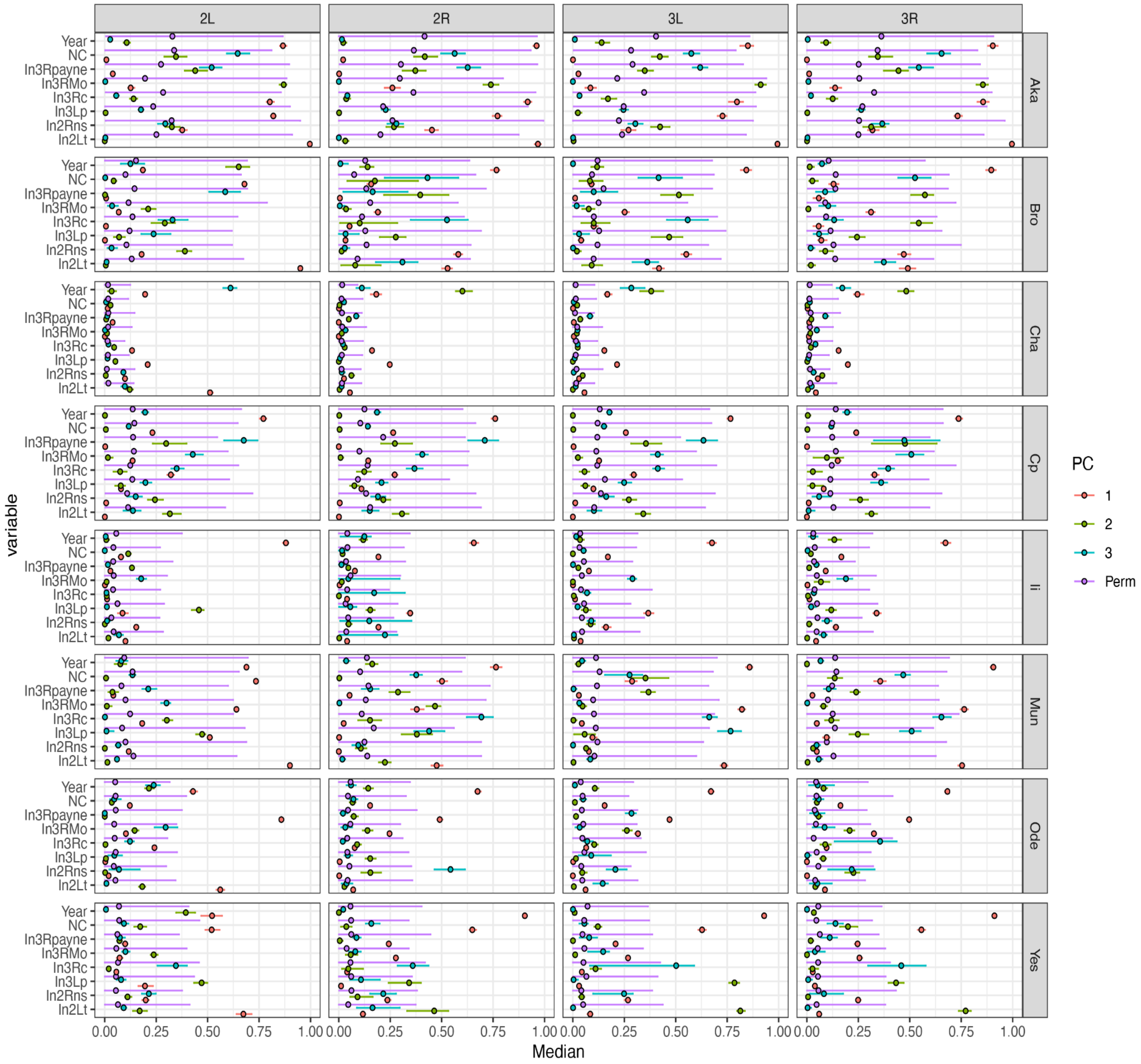

Figure S4: Genetic summary statistics of boom-and-bust simulations. A) Simulations were conducted with variable maximum (N_{Max}) and minimum (N_{Min}) population sizes. **B)** Cartoon model of the simulated overwintering demography, illustrating population dynamics during bottlenecks. **C)** The left facet displays the median pairwise-F_{st} within and between simulation years, while the right facet presents the median r^2 of principal component (PC) and linear discriminant (LD) axes across simulation year. The blue dot marks the location of observed values within the Charlottesville population. **D)** The *loclinear* model of ABC was employed for parameter estimation, and posterior probabilities for N_{Max} and N_{Min} parameters are shown on the left. On the right, Euclidean distances quantify the disparities between cumulative simulation results and observed Charlottesville population statistics. Simulations were only accepted by ABC if they were close enough to the observed data under a tolerance threshold of 5%. **E)** A leave-one-out analysis was performed, excluding each statistic individually (x-axis), with 95% confidence intervals (lines) and median values (points) illustrating the resulting parameter estimates for N_{Max} and N_{Min}. Shaded intervals represent the 95% confidence interval for the *loclinear* ABC output when all statistics were employed, and the dotted line signifies the median value. Deviations from the shaded region indicate the influence of individual statistics on estimated population sizes for N_{Max} and N_{Min}.

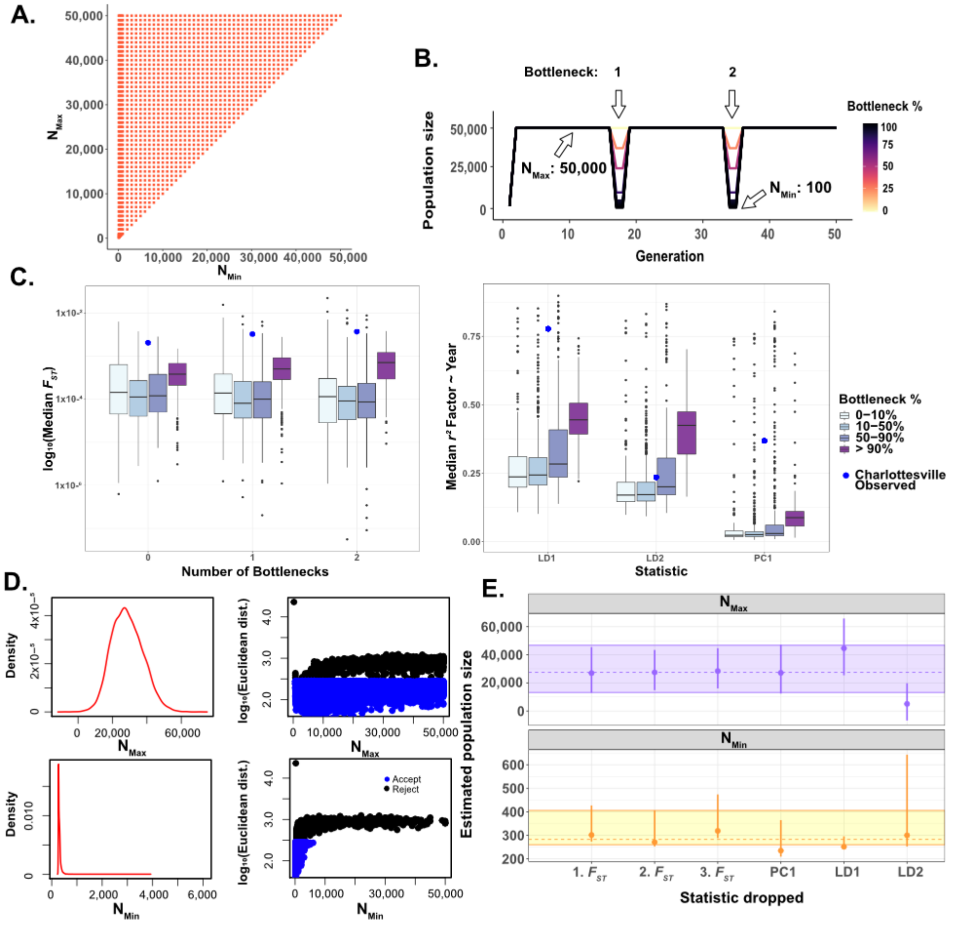

Figure S5: Using the DGRP to characterize mutations associated with the In(2L)t inversion. **A)** PCA of DGRP lines colored by known inversion status. Heterozygous individuals were excluded from the analysis. **B)** Correlation analysis of individual SNPs within 2L to PC 1 shown in panel A. The y-axis shows the P-value (Bonferroni corrected) of the SNP-level correlation to PC 1 shown in panel A. **C)** Levels of linkage disequilibrium among the inversion markers discovered in the DGRP estimated in our Charlottesville data. To determine inversion breakpoint markers, we only kept loci with median LD values among markers of 0.8 or greater. **D)** Results of a support vector machine (SVM) algorithm trained to determine the inversion status of unknown individual samples. The SVM algorithm was trained on the DGRP using the 47 marker loci highlighted on the right side of the dashed red line in C.

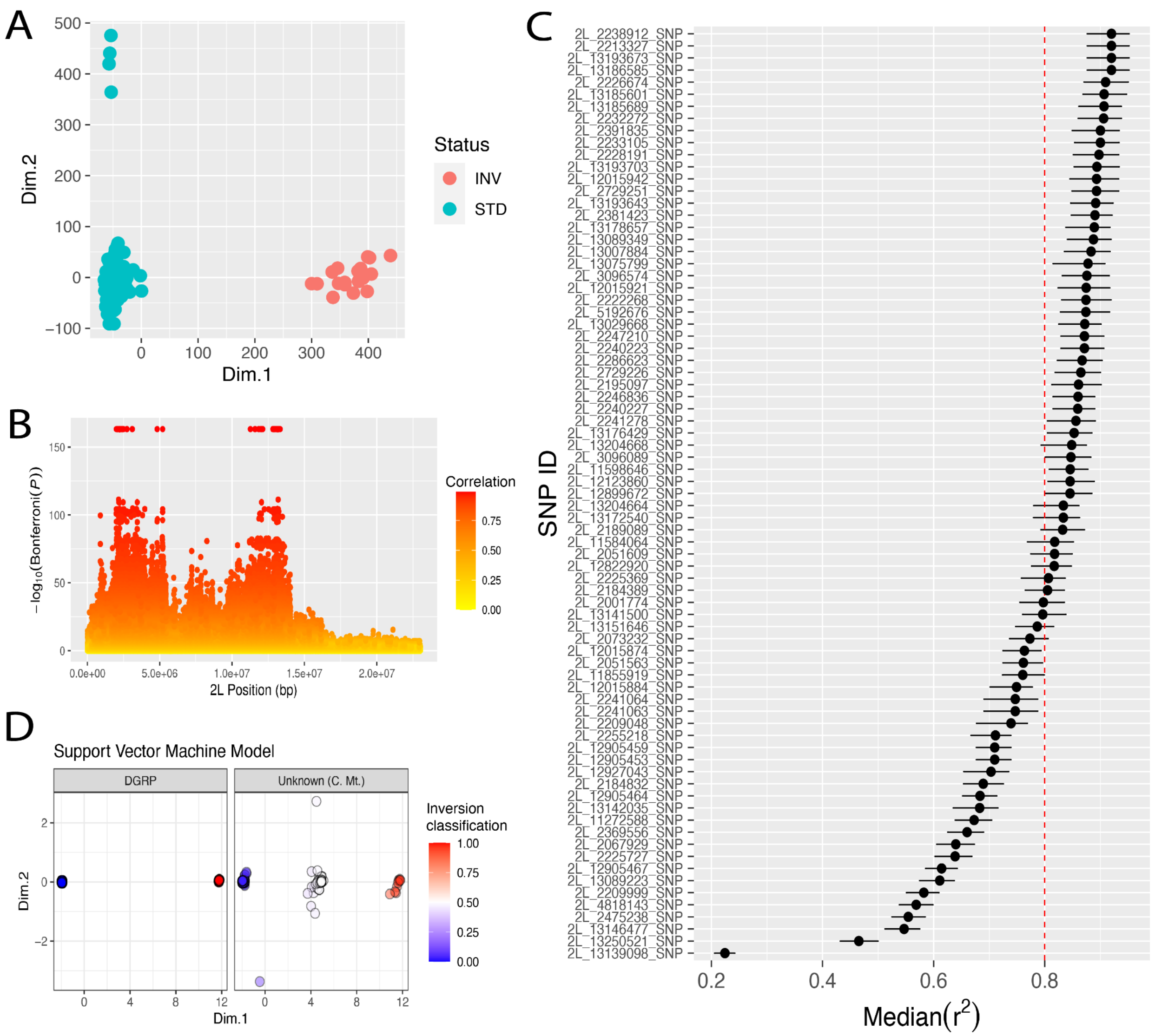

Figure S6: Data summarization scheme using the NASA power dataset for environmental variables. **A)** The x- axis shows the number of days prior to collection that environmental data was summarized. **B)** Green dots indicate that the metric that was used in the model. Including a null and year models, the total number of models tested is 11 x 6 (Temperature) + 11 x 2 (Humidity) + 11 x 2 (Precipitation) + 2 (Null and Year) = 112 models. **C)** Each cartoon represents a single SNP (Y) as a vector of allele frequencies. The X variables are the Year (or Year:Loc) term, and the environmental variables 1-110. Only one environmental term was used for any model (e.g., M1.p or M2.p). For the Year or Year:Loc term, depicted above, identical years are represented by the same color and identical localities by the dashed line. For instance in the DEST model panels, there are two localities depicted. One locality has two samples collected in one year and the other locality has four samples collected over two years. Permutations happen in two stages. In the first stage, the Year (or Year:Locality) term is permuted and tested against the null model. Populations can be assigned to any new Year or (Year:Locality) value. In the second stage, the Year (or Year:Locality) term is the real value for that sample, and only the environmental values are permuted. During the environmental permutation, a sample can take any environmental value.

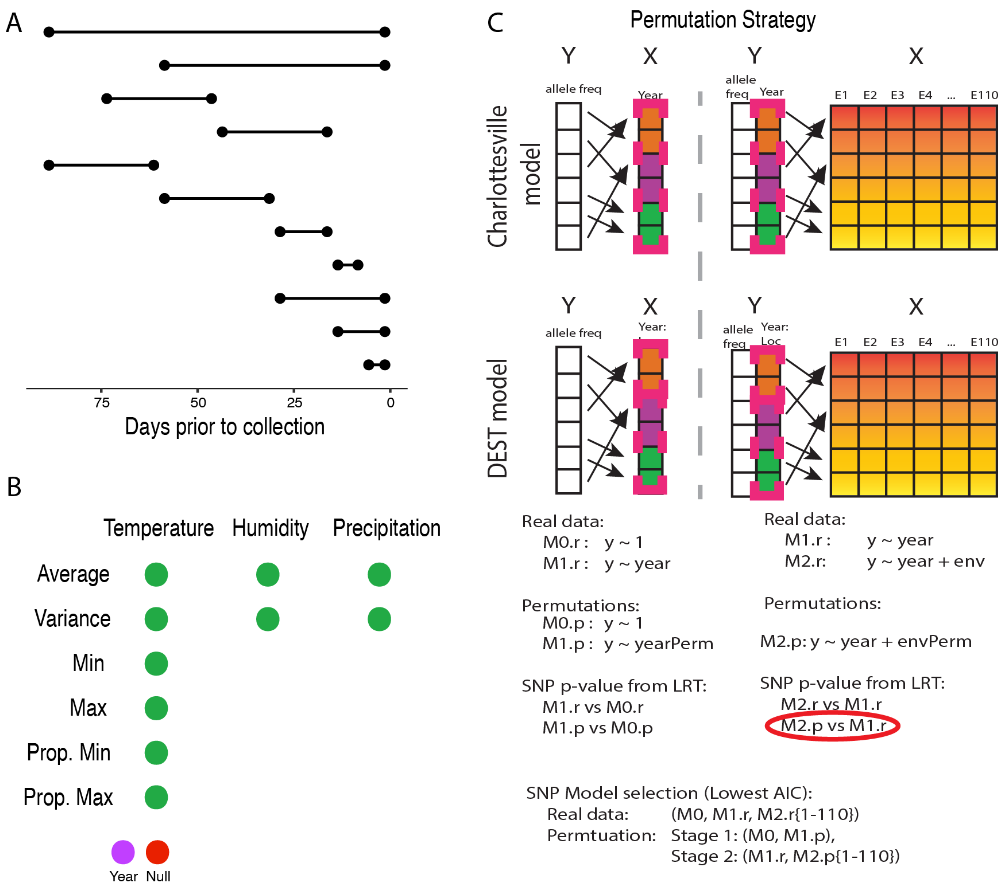

Figure S7: Summary results from *BayPass* analysis. Panels **A)** and **B)** are hexbin plots that show the density of SNPs (color scale). The x-axis for both is the rank-normalized p-value from the T_{max}0-15d GLM model. The y-axis for A) is the XtX_{ST} value and the y-axis for B) is the BF value on a dB scale. The vertical line for A) and B) is the 5% GLM threshold used throughout the study. The horizontal line in (A) is the XtXst value corresponding to the 5% FDR threshold. The horizontal line in B) is the BF value corresponding to the 0.001% FDR threshold from POD simulations. For A) and B), the numbers in each panel represent the proportion of XtX_{ST} of BF outliers in that region of the genome. The region of the genome inside In2Lt has the greatest proportion of both XtX_{ST} and BF outliers compared to the rest of the genome. Panels **C)** and **D)** show the odds ratio of enrichment between top 5% GLM sites and top XtX_{ST} or BF sites. The odds ratios were calculated by tabulating the number of SNPs in each quadrant as defined by the vertical and horizontal thresholds in A) and B). For C) and D) the grey lines represent the distribution of odds-ratios generated from the same enrichment test applied to the permuted GLM. The red line is the odds-ratio for the real GLM. For panel D, the red line is at infinity because all BF outliers are GLM outliers. For C) and D), the numbers in each panel represent the proportion times the odds ratio from the real GLM exceeds the odds ratio from the permuted GLMs.

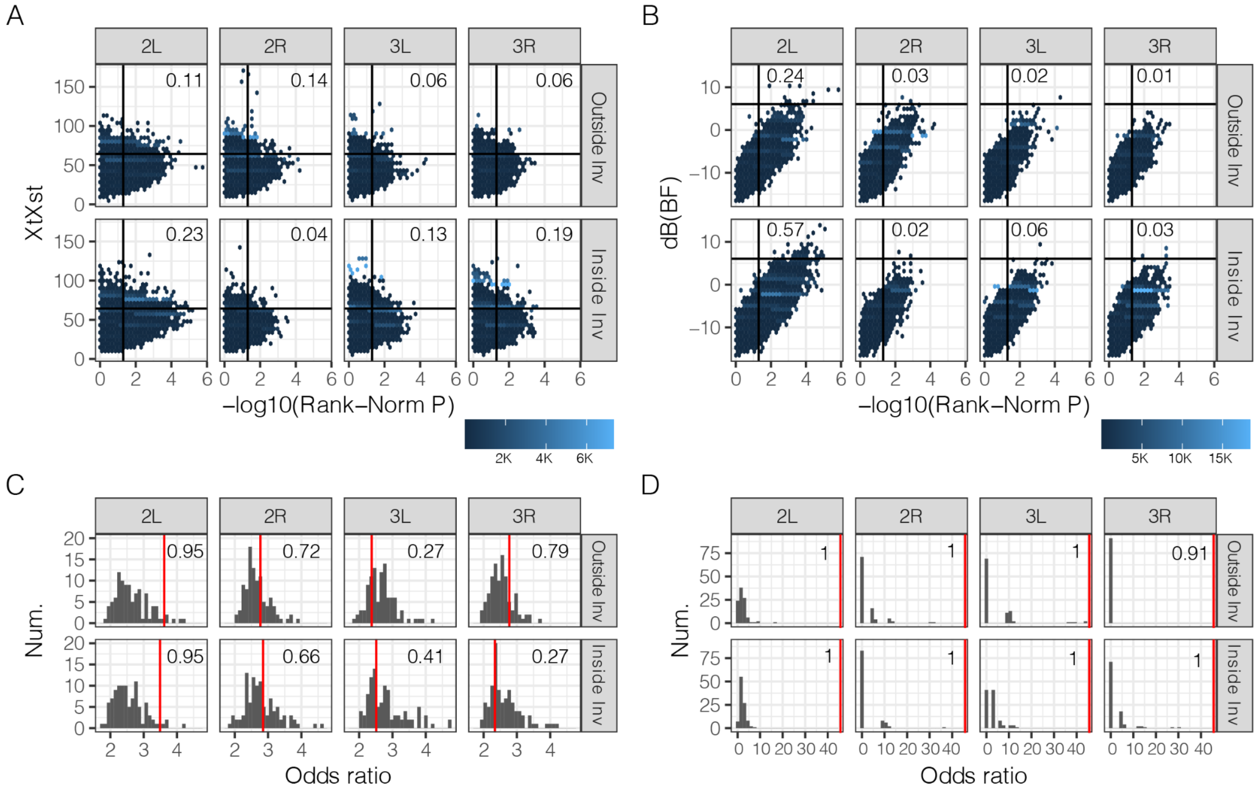

Figure S8: A) Proportion of SNPs, within 0.1 Mb windows, with $r^2 > 0.99$ to the inversion. **B)** Same as A, but $r^2 > 0.70$.

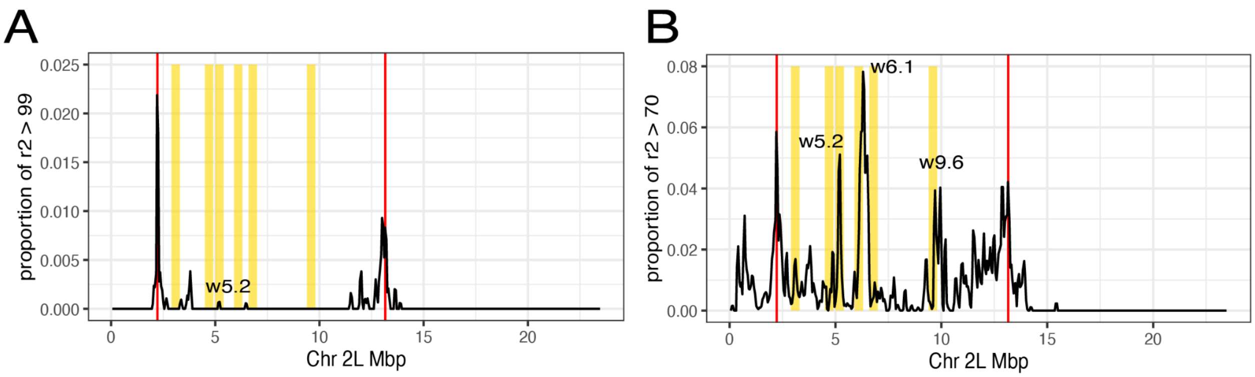

Figure S9: A) Levels of nucleotide diversity (π) within 2L. Blue lines represent standard karyotypes ("Std"). Red lines represent inverted karyotypes ("Inv"). **B)** Distributions of Tajima's D for standard and inverted karyotypes. The values for two windows of interest (6.1 and 9.6) are annotated as arrows, as well as the average value for the entire karyotype. **C)** Mean allele ages across windows of interest in 2L compared to other windows. All individuals (Std/Std, Inv/Inv, Std/Inv) were used in the TMRCA calculation. The estimated age of the inversion breakpoints, ~85,000 y, is marked as a dashed horizontal line. Areas outside of windows are indicated in the turquoise color.

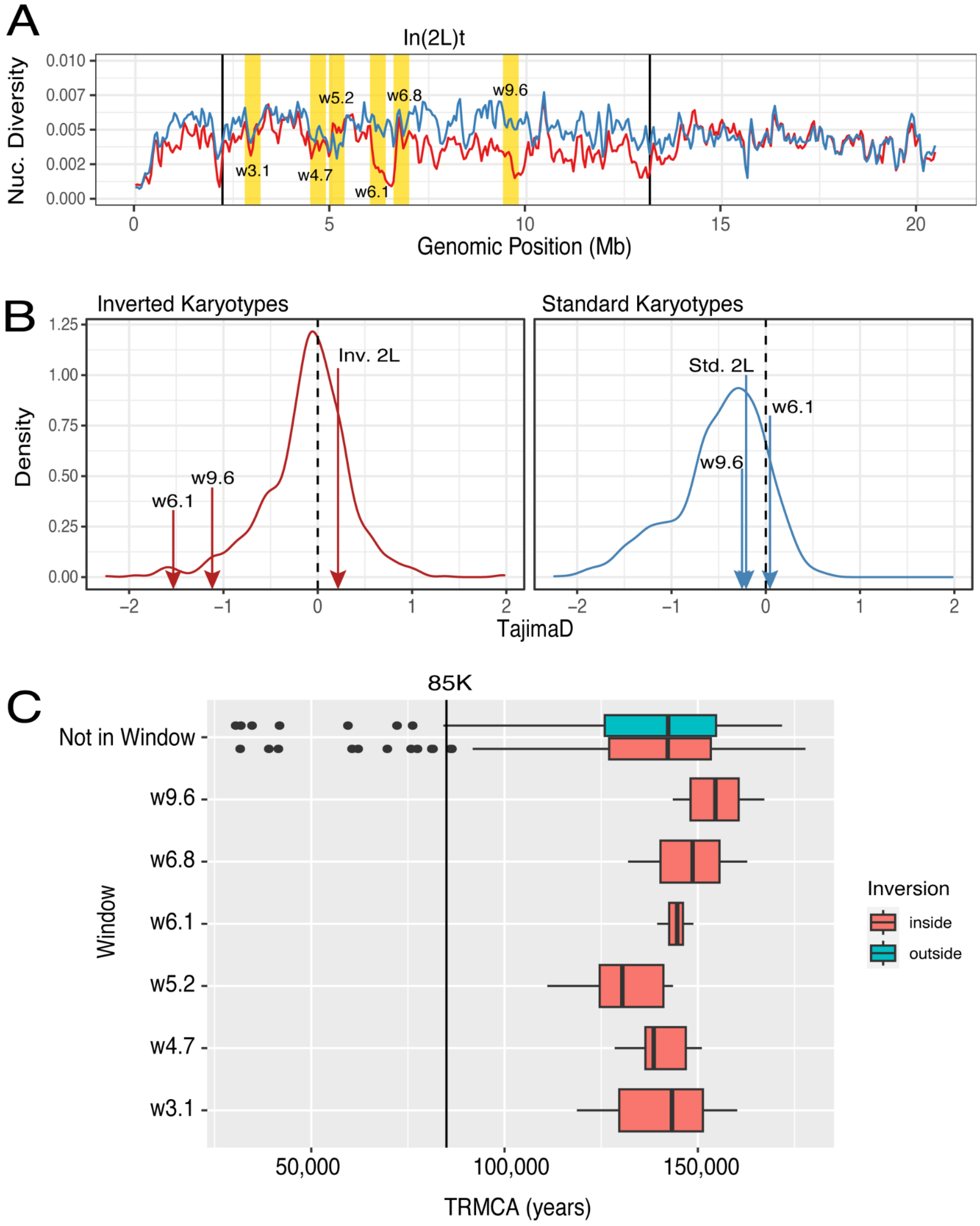

Figure S10: A) Levels of haplotype diversity within inverted and standard classes in chromosome 2L. The y-axis is the number of unique haplotypes across windows of 10 kbp. **B)** Boxplots showing the number of unique haplotypes. The y-axis shows the scaled number of haplotypes (scaled as $(x - \bar{x}) / s$; where x: number of haplotypes across individual 10k windows, $\bar{x}$: mean within the region among 10k windows of interest, s: SD within the region of interest). Summaries are shown in six regions of interest in In(2L)t, as well as regions inside and outside of the inversion. The annotation "Inv" and "Std" refer to the karyotypes In(2L)t and standard, respectively, in chromosome arm 2L. **C)** F_{ST} between the inverted and standard karyotypes in 2L in one DPGP African population (Zambia). Various window (W) and step sizes (S) are shown. The dashed vertical lines indicate the inversion breakpoints for In(2L)t. The blue line represents *Msp300* in w5.2.

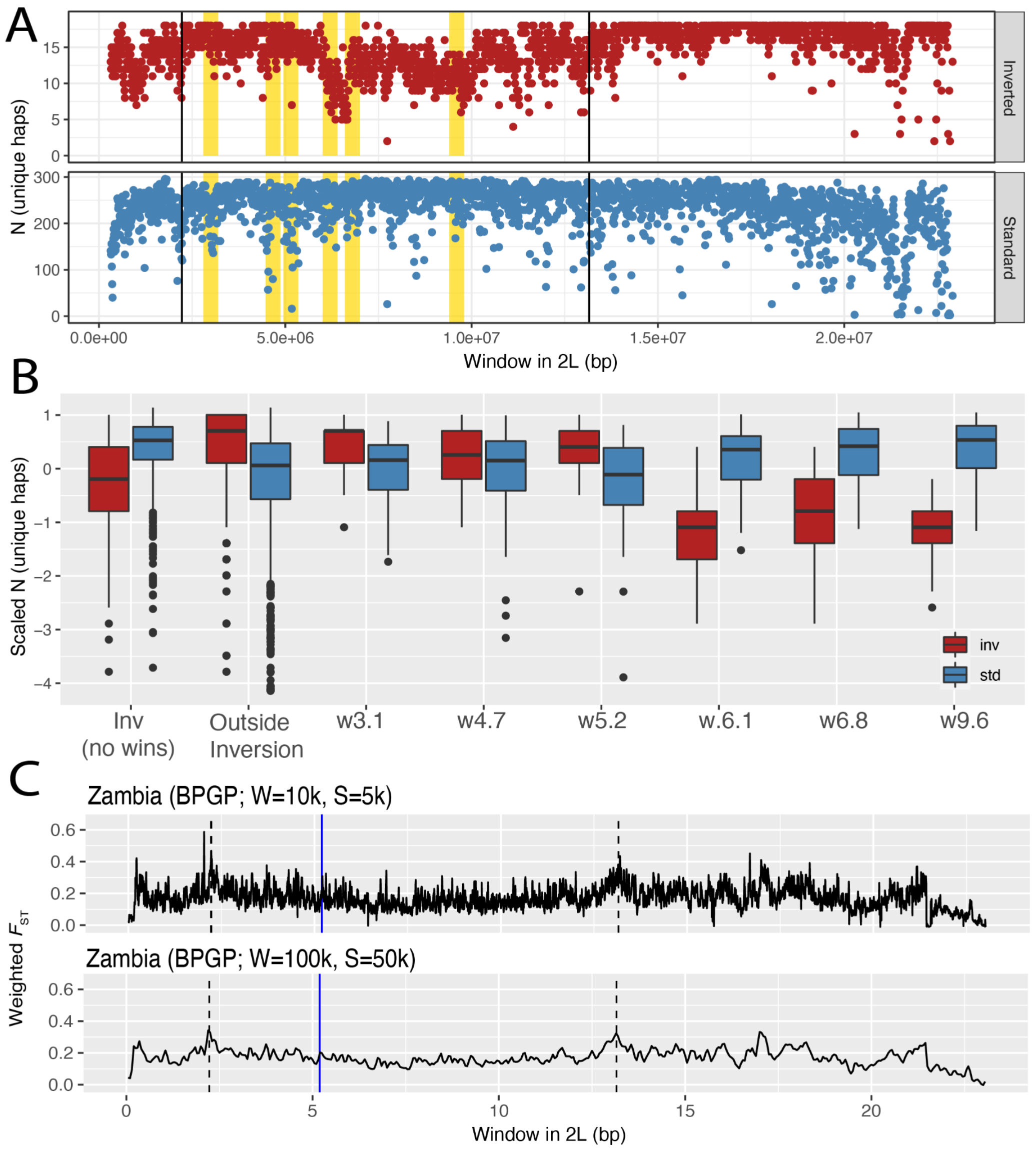

Figure S11: Model search across all population groups. The x-axis shows each model ranked according to the best model in In(2L)t in Virginia. The y-axis shows an enrichment score testing whether the model output was scored as the best model by AIC relative to permutations. Gray circles represent models that are not statistically significant. Circles with colors represent models that are statistically significant. Red circles indicate environmental models, blue squares indicate year x population model. Green triangles represent null models. The best model in In(2L)t in Virginia (see main text) is highlighted across the plot with a black arrow.

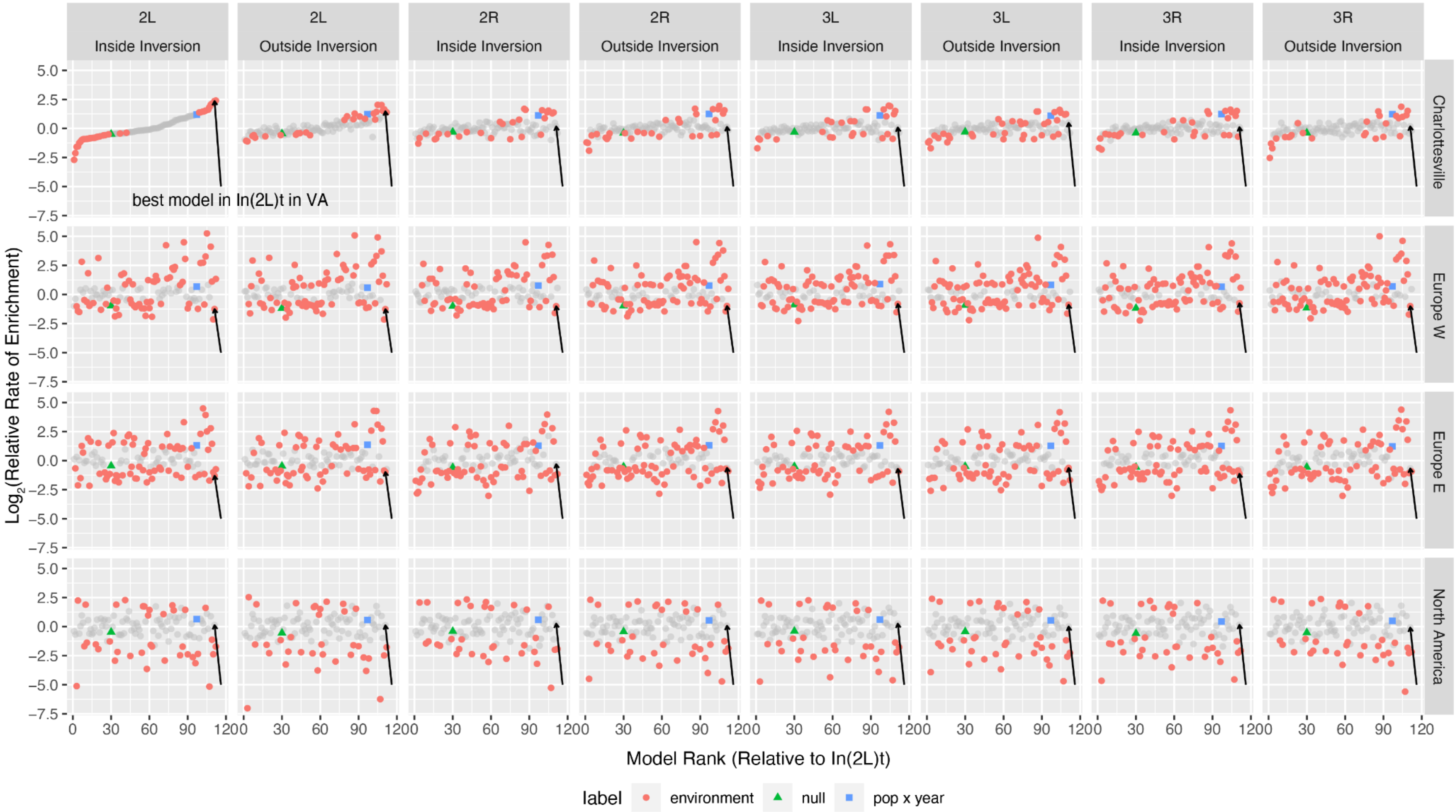

Figure S12: *P*-values of the enrichment test for the best models in EUE and EUW. In(2L)t is demarcated by the vertical lines. Windows of interest in the Charlottesville model are shown with yellow bands. Green lines represent the real data. Red lines represent the 99% quantile of the *P*-values of 100 random permutations.

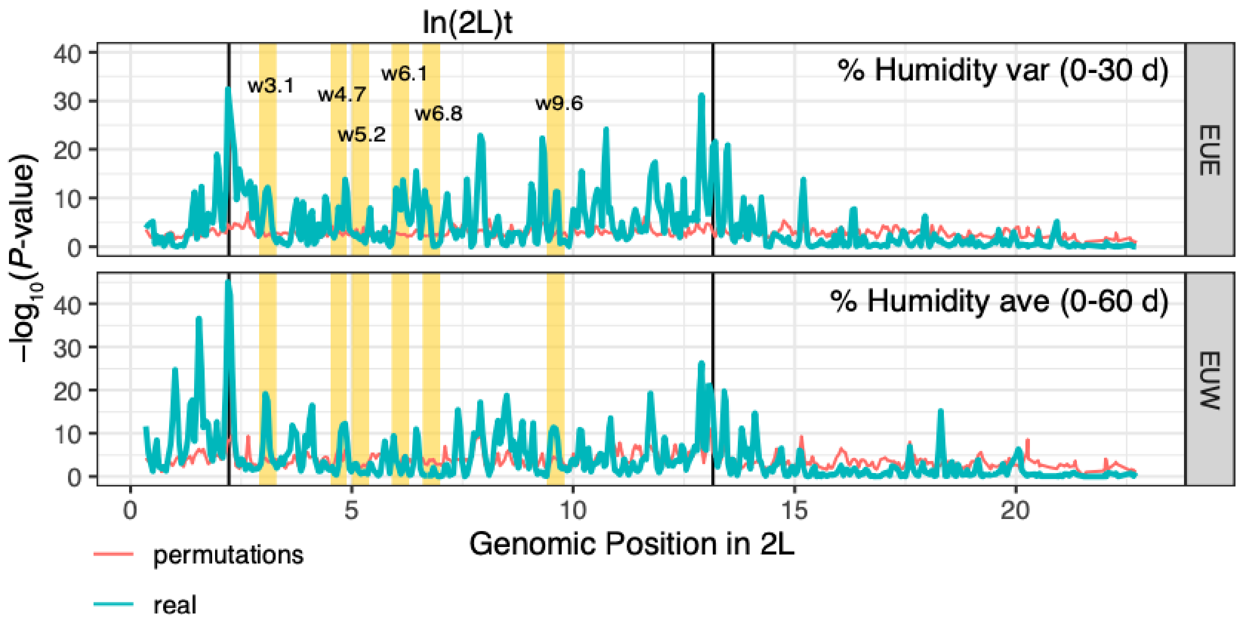

Figure S13: A) The y-axis shows the number of traits used in GWAS that are associated with inversion status in the DGRP for autosomes (For chromosome 3R the test considers whether a trait is associated with any inversion). Traits are divided into four phenotypic categories indicated by color. The real data is shown as diamonds, permutations are shown as black points and boxplots (asterisks indicate that the data outperform 95% of permutations). **B)** Directionality and enrichment analysis between the DGRP-GWAS and the best environmental model in Virginia for 2R, 3L, and 3R. Lines indicate the permutation-based 95% confidence intervals. **C)** Window-level enrichment analysis across the whole genome. Windows that beat permutation are shown in turquoise, otherwise in red. The y-axis shows the SNP-wise number of enriched phenotypes (i.e., significant in both the GLM and GWAS). Inversions are shown as color blocks.

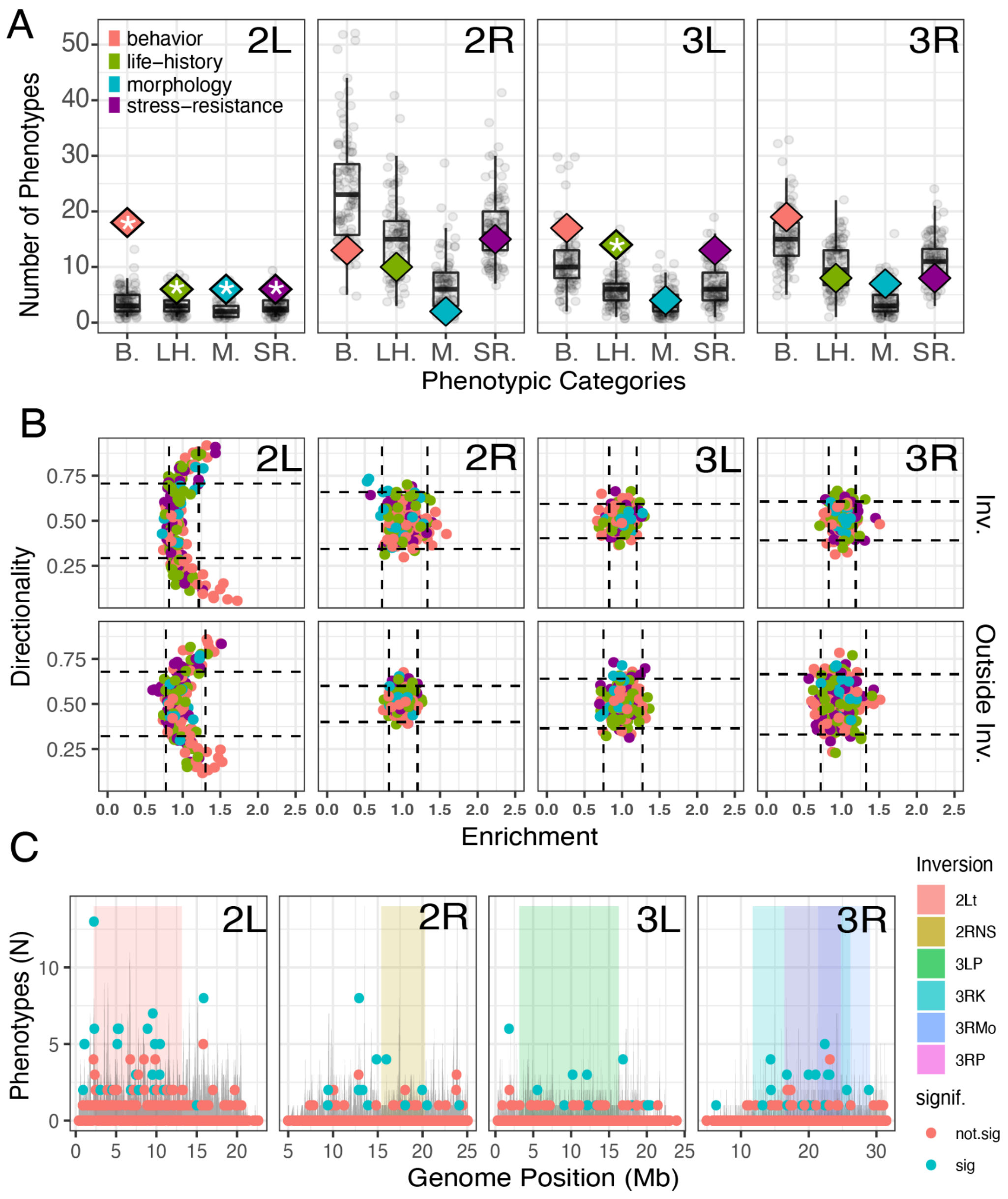

Figure S14: Explanation of DGRP line choice and the complete observations of three major phenotypes from each of the different genetic backgrounds used in the startle response study with the deficiency lines. **A)** The ancestral (red) or derived (blue) background of the 10 DGRP lines used in the study. Lines are shown to be both inverted or standard, and when considering the structure in their genetic backgrounds either in the "A" type or "D" type (i.e., Ancestral or Derived based on the proportion of sites shared or derived relative to *D. simulans*). For example, flies with DGRP 837 background are inverted/type A when considering w5.2, and type D when considering w9.6. **B)** The differences in the three observed phenotypes are shown across genetic groups. Phenotypes are faceted by each of the 5 major deficiencies (see **Table S10, S11**), grouped by the presence of deficiency or balancer in the F1 flies used, and colored by their inversion and type. The top row illustrates the activity slope, a measurement of how rapidly activity decays back to basal following the stimulus event. The second row illustrates the startle duration, a measurement of the period of time between the peak of activity following stimulus, and the return to basal activity. Third row indicates the level of basal activity per minute.

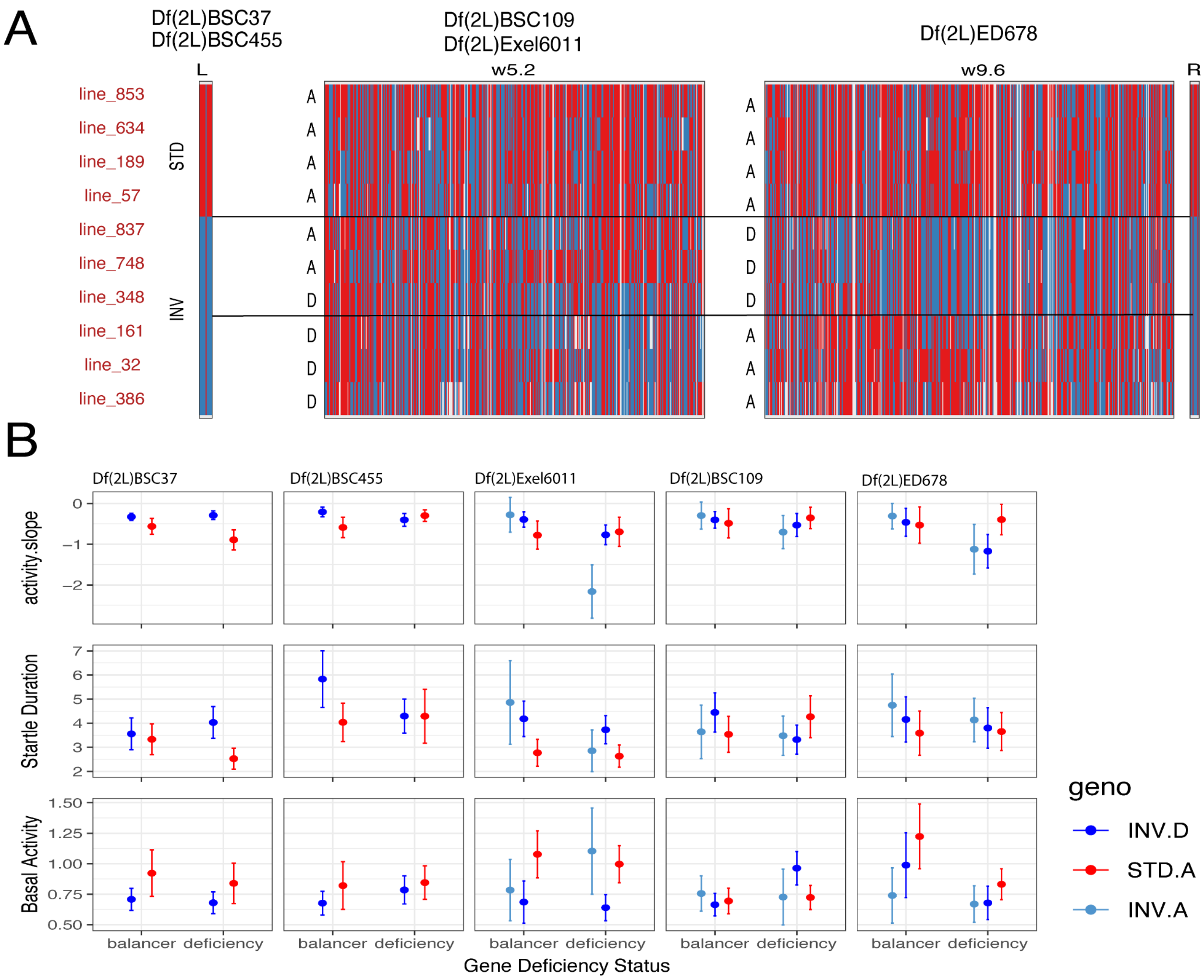

Figure S15: Estimated frequencies of the inversion In(2L)t in a previous study as well as this study, both as a function of month of collection (i.e., a proxy for seasonality). The month-to-month correlation between the patterns of allele frequency between this paper and our study is 0.76 ($P = 0.003$).

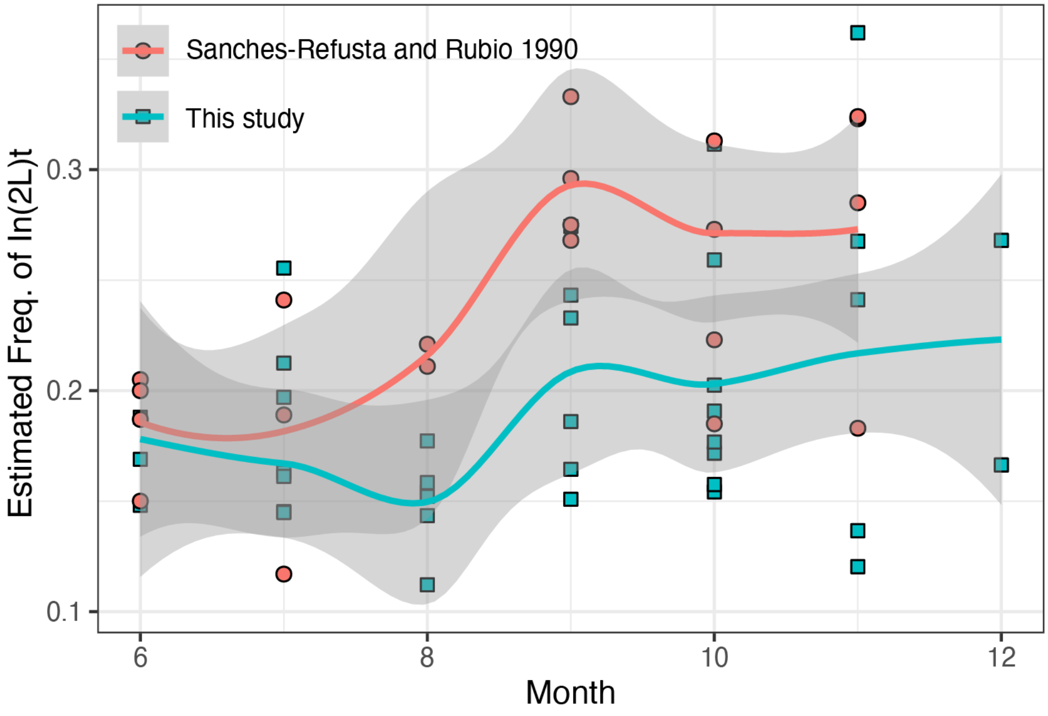

Supplementary References

Bates D., M. Mächler, B. Bolker, and S. Walker, 2015 Fitting Linear Mixed-Effects Models Using **lme4**. J. Stat. Softw. 67. https://doi.org/10.18637/jss.v067.i01

Delaneau O., J.-F. Zagury, M. R. Robinson, J. L. Marchini, and E. T. Dermitzakis, 2019 Accurate, scalable and integrative haplotype estimation. Nat. Commun. 10: 5436. https://doi.org/10.1038/s41467-019-13225-y

Grigoriev I. V., H. Nordberg, I. Shabalov, A. Aerts, M. Cantor, *et al.*, 2012 The Genome Portal of the Department of Energy Joint Genome Institute. Nucleic Acids Res. 40: D26–D32. https://doi.org/10.1093/nar/gkr947

Kapun M., M. G. Barrón, F. Staubach, D. J. Obbard, R. A. W. Wiberg, *et al.*, 2020 Genomic Analysis of European Drosophila melanogaster Populations Reveals Longitudinal Structure, Continent-Wide Selection, and Previously Unknown DNA Viruses, (D. Falush, Ed.). Mol. Biol. Evol. 37: 2661–2678. https://doi.org/10.1093/molbev/msaa120

Kapun M., J. C. B. Nunez, M. Bogaerts-Márquez, J. Murga-Moreno, M. Paris, *et al.*, 2021a Drosophila *Evolution over Space and Time (DEST) - A New Population Genomics Resource*. Genomics.

Kapun M., J. C. B. Nunez, M. Bogaerts-Márquez, J. Murga-Moreno, M. Paris, *et al.*, 2021b *Drosophila* Evolution over Space and Time (DEST): A New Population Genomics Resource, (R. Nielsen, Ed.). Mol. Biol. Evol. 38: 5782–5805. https://doi.org/10.1093/molbev/msab259

Li H., B. Handsaker, A. Wysoker, T. Fennell, J. Ruan, *et al.*, 2009 The Sequence Alignment/Map format and SAMtools. Bioinformatics 25: 2078–2079. https://doi.org/10.1093/bioinformatics/btp352

Li H., 2013 Aligning sequence reads, clone sequences and assembly contigs with BWA-MEM. https://doi.org/10.48550/ARXIV.1303.3997

Machado H. E., A. O. Bergland, R. Taylor, S. Tilk, E. Behrman, *et al.*, 2021 Broad geographic sampling reveals the shared basis and environmental correlates of seasonal adaptation in Drosophila, (M. Nordborg, P. J. Wittkopp, and M. Nordborg, Eds.). eLife 10: e67577. https://doi.org/10.7554/eLife.67577

Morgulis A., E. M. Gertz, A. A. Schaffer, and R. Agarwala, 2006 WindowMasker: window-based masker for sequenced genomes. Bioinformatics 22: 134–141. https://doi.org/10.1093/bioinformatics/bti774

Okonechnikov K., A. Conesa, and F. García-Alcalde, 2015 Qualimap 2: advanced multi-sample quality control for high-throughput sequencing data. Bioinformatics btv566. https://doi.org/10.1093/bioinformatics/btv566

Patterson M., T. Marschall, N. Pisanti, L. van Iersel, L. Stougie, *et al.*, 2015 WhatsHap: Weighted Haplotype Assembly for Future-Generation Sequencing Reads. J. Comput. Biol. J. Comput. Mol. Cell Biol. 22: 498–509. https://doi.org/10.1089/cmb.2014.0157

Searle S. R., F. M. Speed, and G. A. Milliken, 1980 Population Marginal Means in the Linear Model: An Alternative to Least Squares Means. Am. Stat. 34: 216–221. https://doi.org/10.1080/00031305.1980.10483031

Van der Auwera G., and B. O'Connor, 2020 *Genomics in the Cloud: Using Docker, GATK, and WDL in Terra*.
